# A mitotic recombination map proximal to the *APC *locus on chromosome 5q and assessment of influences on colorectal cancer risk

**DOI:** 10.1186/1471-2350-10-54

**Published:** 2009-06-10

**Authors:** Kimberley Howarth, Susanna Ranta, Eitan Winter, Ana Teixeira, Helmut Schaschl, John J Harvey, Andrew Rowan, Angela Jones, Sarah Spain, Susan Clark, Thomas Guenther, Aengus Stewart, Andrew Silver, Ian Tomlinson

**Affiliations:** 1 Molecular and Population Genetics Laboratory, Wellcome Trust Centre for Human Genetics, University of Oxford, Roosevelt Drive, Oxford, OX3 7BN, UK; 2 London Research Institute, Cancer Research UK, 44, Lincoln's Inn Fields, London, WC2A 3PX, UK; 3 Polyposis Registry St Mark's and Northwick Park Hospitals, Watford Road, Harrow, HA1 3UJ, UK; 4 Academic Histopathology, St Mark's and Northwick Park Hospitals, Watford Road, Harrow, HA1 3UJ, UK; 5 Colorectal Cancer Genetics Group, Institute of Cell and Molecular Sciences, Bart's and the London Medical School, Queen Mary College, University of London, London, UK

## Abstract

**Background:**

Mitotic recombination is important for inactivating tumour suppressor genes by copy-neutral loss of heterozygosity (LOH). Although meiotic recombination maps are plentiful, little is known about mitotic recombination. The *APC *gene (chr5q21) is mutated in most colorectal tumours and its usual mode of LOH is mitotic recombination.

**Methods:**

We mapped mitotic recombination boundaries ("breakpoints") between the centromere (~50 Mb) and *APC *(~112 Mb) in early colorectal tumours.

**Results:**

Breakpoints were non-random, with the highest frequency between 65 Mb and 75 Mb, close to a low copy number repeat region (68–71 Mb). There were, surprisingly, few breakpoints close to *APC*, contrary to expectations were there constraints on tumorigenesis caused by uncovering recessive lethal alleles or if mitotic recombination were mechanistically favoured by a longer residual chromosome arm. The locations of mitotic and meiotic recombination breakpoints were correlated, suggesting that the two types of recombination are influenced by similar processes, whether mutational or selective in origin. Breakpoints were also associated with higher local G+C content. The recombination and gain/deletion breakpoint maps on 5q were not, however, associated, perhaps owing to selective constraints on *APC *dosage in early colorectal tumours. Since polymorphisms within the region of frequent mitotic recombination on 5q might influence the frequency of LOH, we tested the 68–71 Mb low copy number repeat and nearby tagSNPs, but no associations with colorectal cancer risk were found.

**Conclusion:**

LOH on 5q is non-random, but local factors do not greatly influence the rate of LOH at *APC *or explain inter differential susceptibility to colorectal tumours.

## Background

Germline mutations in the *APC *gene (chromosome 5q21) result in familial adenomatous polyposis (FAP, OMIM 175100), a dominantly inherited colorectal tumour predisposition syndrome. Adenomas start to grow in the colorectum of patients with FAP when a cell acquires a somatic "second hit" at the *APC *locus, leading to loss of critical APC functions [1]. The position and type of the "second hit" in FAP are non-random and depend on the localization of the germline mutation. FAP patients with germline *APC *mutations around codon 1300 mainly acquire "second hits" by loss of heterozygosity in their colorectal tumours and these patients generally develop severe disease; by comparison, colorectal tumours in patients with germline mutations outside this region are associated with "second hits" in the form of protein-truncating mutations [2]. Somatic *APC *mutations also occur in up to 85% of sporadic colorectal tumours and a similar association between "first hit" and "second hit" is seen. In general, as a result of these selective constraints on *APC *in early tumours, LOH at *APC *occurs by mitotic recombination (break-induced replication), causing reduction to homozygosity but no copy number change [3].

LOH probably occurs most often as a result of repair of a DNA double-stand break (DSB). Unrepaired DSBs can cause LOH by deletion. However, the cell has several ways to repair DSBs, those most commonly used being non-homologous end-joining or homologous recombination repair [4]. DSBs are thus an important initiator of recombination events. Using the sister chromatid as a template for recombination is preferred, as this results in precise repair with no loss of sequence information (sister chromatid exchange, SCE). Such events are generally genetically undetectable. In contrast, if the non-sister chromatid of the homologous chromosome acts as a template for mitotic recombination, half of the daughter cells will have loss of heterozygosity (LOH) distal to the recombination breakpoint site after cell division. LOH by mitotic recombination can therefore be seen as a form of DNA repair, but it is only partially effective. Defects in mitotic recombination underlie rare conditions such as Bloom and Werner syndromes (OMIM 210900, OMIM 277700, ), and these syndromes are associated with an elevated cancer risk. In tumours, LOH by mitotic recombination often involves large chromosomal segments extending from several megabases to whole chromosome arms.

Meiotic recombination has been the subject of numerous studies, but little is understood about the underlying factors that lead to mitotic recombination. The studies of meiotic recombination have shown, for example, that the locations of crossovers cluster and vary according to local sequence context and that the probability of recombination can be associated with common germline sequence variants [5,6]. In one of the view studies to date, Hagstrom et al [7] mapped the locations of mitotic recombination 'breakpoints' in retinoblastomas proximal to the *RB1 *locus on chromosome 13q. Suggestive evidence of breakpoint clustering was found, although the map density was relatively low.

In this study, we set out to improve our understanding of mitotic recombination by characterising LOH breakpoints proximal to the *APC *locus in colorectal tumours. We examined the relationship of these breakpoints to sequence features. Furthermore, we hypothesised that if the likelihood of mitotic recombination at *APC *depended on local sequence variants, there would be clinical implications, because individuals with an inherited tendency to LOH at *APC *might have an increased risk of colorectal cancer. We sought out to test this hypothesis by relating sequence variants close to the recombination breakpoints to colorectal cancer susceptibility.

## Methods

### Samples

DNA was extracted from 54 CRC cell lines (C10, C32, C70, C75, C80, C99, C106, C125PM, C170, CACO2, CL11, COLO201, COLO205, COLO320, COLO678, COLO741, CX1, GP5D, H508, H716, HCA7, HCA46, HCT8, HCT116, HCT15/DLD1, HRA19, HT29, HT55, HT115, HUTU80, LOVO, LIM1863, LS1034, LS123, LS174T, LS411, MBDA8, NCI-H747, PC/JW, RKO, SKCO1, SNUC2B, SW1116, SW1222, SW1417, SW403, SW48, SW620, SW837, SW948, T84, VACO4S, VACO5, VACO10MS).

Research was undertaken in compliance with the Helsinki Declaration and with full ethical approval (Oxfordshire REC B, 05/Q1605/66). 268 anonymised, formalin-fixed, paraffin-embedded (FFPE) samples of colorectal adenomas from thirteen FAP patients with FAP were identified. In order to increase the frequency of tumours showing LOH, we selected cases with germline mutations around codon 1300. We also used larger lesions (> 0.5 cm diameter) to minimise problems of polyclonality in smaller colorectal adenomas [8]. For 10 of the larger polyps (diameter ≤ 0.9 cm), more than one sample was obtained from different parts of the same polyp in order to check for consistency of the molecular data, although in each case, complete concordance was observed. After enrichment for dysplastic epithelium using a fine gauge needle and the dissecting microscope, DNA was extracted from each adenoma using a standard proteinase K digestion and the Qiagen DNeasy kit, except that elution with water was undertaken twice to increase the yield. Normal tissue from the same block was extracted using the same method.

For analysis of associations between mitotic recombination breakpoints and colorectal cancer risk, two case-control series were analysed: (i) cases and controls from the UK CORGI study of familial colorectal tumour patients [9]; and (ii) cases from the VICTOR  and QUASAR 2  clinical trials and controls from the UK 1958 Birth Cohort . All cases and controls, comprising about 4,000 samples in total, were of white UK ethnic origin. Further details of ascertainment, inclusion criteria and exclusion criteria can be found in [9]. Genotyping data were derived from the Illumina Hap300, Hap370 or Hap550 arrays (see below). Each series was analysed separately for significant allele or genotype frequency differences between cases and controls, followed by a weighted meta-analysis using the Mantel-Haenszel method in STATA9.0.

### SNP microarray analyses

Patient and colorectal cancer cell line DNAs were prepared and hybridised to arrays (Affymetrix 10 K HuSNP and Illumina Hap300, Hap370 and Hap550) using the manufacturer's standard protocols. Genotyping calls were made using the manufacturer's software, resulting in call rates of over 98% and sample failure rates of < 2% using good quality DNA. LOH and copy number changes were scored using the manufacturer's software in each case, supplemented by visual inspection of allele frequency plots along each chromosome.

For the custom Goldengate arrays, designed to assess LOH on 5q in FFPE tumour samples, the Illumina custom probe design software was used to test the suitability of all SNPs from dbSNP126 that mapped between the chromosome 5 centromere and the *APC *locus (~112 Mb). After elimination of SNPs with low design scores, a panel of 360 remained. An additional 11 SNPs distal to the *APC *gene were chosen to provide evidence that LOH extended as far as the telomere; 7 were located on chromosome 5p in order to give evidence of whole-chromosome LOH; and 10 SNPs mapped elsewhere in the genome to give evidence of copy number changes. 200–300 ng of each DNA was hybridised to the arrays using the manufacturer's standard protocols. After excluding SNPs with Gentrain scores of less than 0.3, the Illumina Beadstudio software was used to indicate LOH and copy number change on 5q, either in unpaired sample mode (for CRC cell lines) or, after training on FFPE samples, in paired mode for the colorectal adenomas. We found, however, that visual inspection of allelic binning and manual corrections of clear errors by two or more independent observers (SR, KH, IT) improved data quality and allowed breakpoints to be mapped more closely.

### Microsatellite and analysis

Chromosome 5q microsatellite markers (D5S623, D5S664, D5S407, D5S398, D5S2107, D5S624, D5S1990, D5S2089, D5S647, D5S2019, D5S2003, D5S2041, D5S2029, D5S107, D5S644, D5S669, D5S346) and in-del polymorphisms (rs2067135, rs1305058, rs3087334, rs2307799, rs1610940) were chosen according to their location from the human genome March 2006 build . PCR genotyping used a single dye-labelled primer, the ABI 3730 sequencer and the Genescan/Genotyper software. For analysis of CRC cell lines, absence of one allele was required to score possible LOH. The fact that paired constitutional DNA was not available in most cases meant that this approach was more useful for indicating definite retention of homozygosity than mapping LOH.

### Pyrosequencing

According to the manufacturer's standard protocols, a Pyrosequencing assay was designed to discriminate between the *SMN1 *and *SMN2 *genes using a C>T substitution [10] corresponding to SNP rs4916 (location chr5:69,408,109 in the March 2006 Human Genome Build). Briefly, primers were designed using the proprietary Pyrosequencing assay design software, giving: (i) biotinylated forward PCR primer, TCCTTTATTTTCCTTACAGGGTTT; (ii) reverse PCR primer, ATGCTGGCAGACTTACTCCTTAAT; and sequencing primer, TCCTTCTTTTTGATTTTGT. Allelic intensities were derived from the standard Pyrosequencing software and used to derive genotypes (see below).

## Results

### Mitotic recombination breakpoint mapping in colorectal cancer cell lines

We initially screened 49 colorectal cancer cell lines for copy-neutral LOH at *APC *using the Affymetrix 10 K HuSNP array, alongside array-CGH analysis using a 1 Mb density BAC-based array. Of the 14 cases that had undergone copy-neutral LOH involving *APC*, 10 showed break-induced replication/mitotic recombination events, extending from a location between the centromere and the *APC *locus on chromosome 5q to the long-arm telomere. In 4 of these 10 lines (C10, VACO4S, CACO2 and C70), the LOH breakpoint was probably within or close to a small region, 67.5–72.0 Mb, the site of a complex low copy number repeat (LCR). Further genotyping using highly polymorphic microsatellite markers and in/del polymorphisms confirmed the locations of these breakpoints and showed that they were likely to lie within or just distal to the LCR (see Additional File 1). However, poor coverage of polymorphic markers within the LCR prevented finer scale mapping.

### Mitotic breakpoint mapping using a custom SNP array in colorectal tumours

The preliminary observation from the colorectal cancer cell lines that there might be a non-random distribution of mitotic recombination breakpoints between the chromosome 5 centromere (~50 Mb) and *APC *prompted us to examine this phenomenon in a larger set of tumours. In order to enrich for tumours with LOH [2] and to reduce the frequency of confounding background events – such as copy number changes related to chromosomal instability in late lesions – we analysed early colorectal adenomas from FAP patients who harboured germline mutations close to codon 1300 of *APC*. Since these lesions were generally available as FFPE specimens, we set up a custom SNP array (see Additional File 2) using the Illumina Goldengate platform that has previously proved suitable for LOH analysis [11].

**Figure 1:**
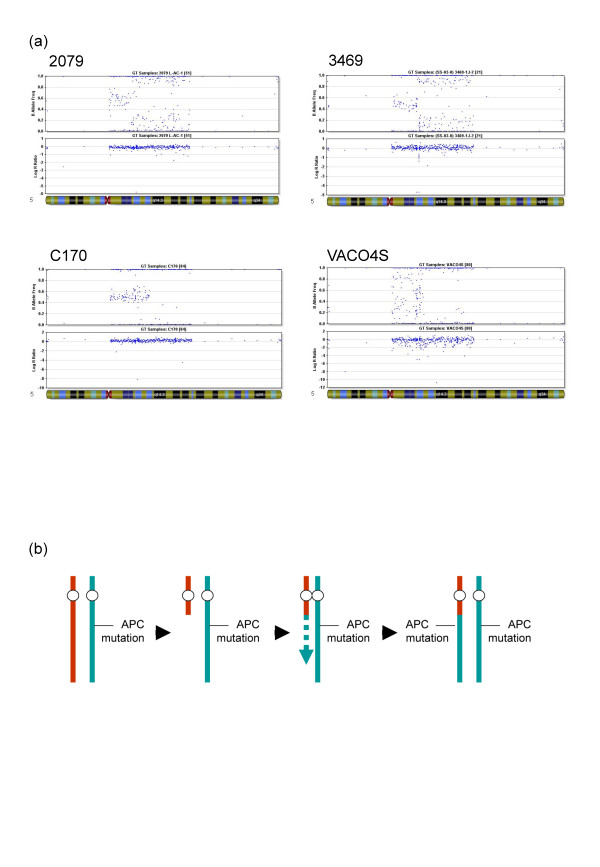
**(a) Examples and (b) probable mechanism of LOH by mitotic recombination involving APC**. (a) Illumina Beadstudio B allele frequency (upper) and Log R ratio plots from colorectal adenomas 3469-1-J-2 (breakpoint, 68–70 Mb) and 2079-L-AC-1 (66–69 Mb). CRC cell lines C170 (~79 Mb) and VACO4S (70–75 Mb) are shown for comparison. LOH is indicated by the splitting of the heterozygous calls around the B = 0.5 region. Note the copy number loss in 3469-1-J-2 at the mitotic recombination boundary, suggesting germline copy number variation or deletion of sequences as a result of recombination. (b) A highly simplified version of LOH by mitotic recombination. It is envisaged that a protein-truncating mutation in *APC *is present on one copy of chromosome 5. The other chromosome 5 then suffers a double strand break on its long arm proximal to *APC*. Rather than using a sister chromatid for repair (for example, if the break has not occurred during or after S phase), repair is effected (dashed line) using the other copy of chromosome 5 as a template. This causes the distal long arms of the two chromosome 5 s, including the *APC *mutation, to become genetically identical and hence LOH at *APC *occurs.

After verification using the colorectal cancer cell lines and having excluded a small number of samples with 5q copy number changes, 75/268 adenomas were both successfully analysed and showed LOH involving *APC *(Figure 1a). Apart from one loss of the whole chromosome, all LOH events began at a breakpoint between the centromere and *APC *and extended as far as juxta-telomeric SNPs, consistent with a mechanism of mitotic recombination through break-induced replication (Figure 1b). No copy-neutral LOH involving an interstitial region of the chromosome arm was detected. Moving along the chromosome arm from the centromere, we mapped each breakpoint to a region between the last constitutionally heterozygous SNP showing no LOH to the first heterozygous SNP with unequivocal LOH. In some cases, localisation to a few kb was possible, but in other cases, such as regions where SNP density was low owing to repeated sequences, where SNPs were poorly informative, or where some SNPs had ambiguous or discordant allelic calls, breakpoints could only be mapped to a few megabases. Breakpoint positions were therefore placed into 'bins' of 5 Mb regions.

**Figure 2:**
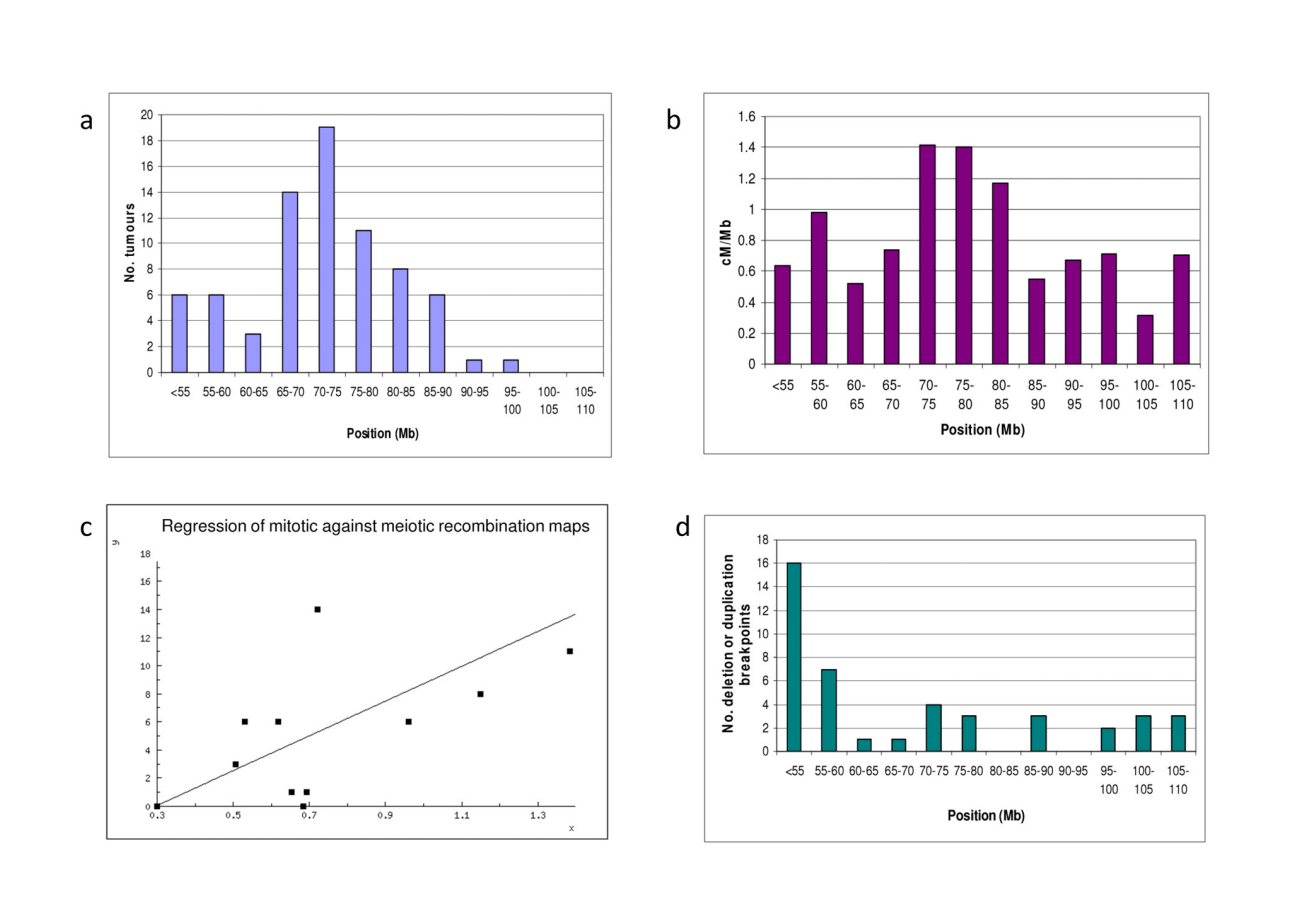
**Locations of mitotic recombination breakpoints and comparison with meiotic recombination and deletion breakpoints**. (a) Frequencies and positions (in 5 Mb bins along chromosome 5) of mitotic recombination breakpoints in colorectal adenomas are shown. There is evident deviation from the flat distribution expected if cross-overs were random. (b) A plot of meiotic recombination breakpoints, expressed as cM/Mb, is shown, derived from the deCODE genetic map. (c) Linear regression of mitotic recombination frequency (y-axis) against meiotic recombination frequency (x-axis) is shown. (d) Frequencies and positions (in 5 Mb bins along chromosome 5) of deletion/duplication breakpoints in colorectal carcinomas are shown; copy number changes on 5q are very rare in adenomas. There is evident deviation from the flat distribution expected, largely as a result of clustering at the centromere.

Examination of the locations of the mitotic recombination breakpoints (Figure 2a) showed them to be non-random compared with the expected flat distribution (χ^2 ^= 33.8, 11 df, p < 0.001). There was a maximum frequency of breakpoints between 65 Mb and 75 Mb, with an elevated frequency extending as far as 80 Mb. There was actually a deficiency of breakpoints close to the *APC *locus. These findings were consistent with our colorectal cancer cell line data. Owing to poor SNP density within the 68–71 Mb LCR itself – because of the presence of multiple repeated sequences and the inability to distinguish true SNPs from variants occurring in paralogous sequences *in cis *– we were unable to fine-map our mitotic recombination breakpoints to determine whether they lay within the LCR or just outside it.

### Comparison between the mitotic and meiotic recombination maps

We compared the locations of the mitotic recombination breakpoints with the meiotic recombination rate reported by deCODE . We calculated the mean meiotic recombination rate within 5 Mb blocks between 50 Mb and 110 Mb on chromosome 5 (Figure 2b) and compared it with the frequencies of mitotic recombination breakpoints that we had found. Although the meiotic recombination map was more uniform than the mitotic map along the chromosome arm, linear regression analysis (Figure 2c) showed that there was a strong association between the locations of the breakpoints of the two forms of recombination (r = 0.729368, t = 3.449, d.f. = 10, p = 0.0062, 2-tailed).

### Comparison between mitotic recombination and gain/deletion of chromosome 5q in colorectal tumours

Although mitotic recombination is the foremost mechanism of loss of heterozygosity in colorectal tumours, some colorectal carcinomas acquire deletions or gains of 5q around *APC *as an additional, later event during tumorigenesis [12]. Were the mitotic recombination map to be influenced primarily by chromosome breakage, we would expect the copy number and mitotic recombination breakpoint maps to be similar. Using array-CGH data from the panel of 54 CRC cell lines and from 99 fresh-frozen cancers (most samples taken from [12]), we determined the positions of breakpoints associated with regions of copy number change between the chromosome 5 centromere and *APC *(Figure 2d). There was no correlation between the locations of mitotic recombination and copy number breakpoints (r = 0.73, t = 3.459, d.f. = 10, p = 0.0062, 2-tailed), primarily because many copy number breakpoints were close to the 5 centromere (~50–56 Mb). Apart from the peri-centromeric region, the site with the highest frequency of copy number breakpoints was 70–75 Mb, as it was for the mitotic and meiotic recombinbation maps, but only a small proportion of all copy number breakpoints was at this site.

### Local sequence context and mitotic recombination breakpoints

We tested whether local G+C content in 5 Mb regions between the chromosome 5 centromere and *APC *was associated with the frequency of mitotic recombination breakpoints. A mean of 38.0% G+C content was found, with a maximum of 40.7% at 75–80 Mb and a minimum of 35.5% at 100–105 Mb. There was a strong correlation between the location of mitotic recombination breakpoints and G+C content (r = 2.85, t = 3.67, d.f. = 10, p = 0.004, 2-tailed).

### A genotyping assay to distinguish between the two major haplotypes within the polymorphic chr5:68–71 Mb LCR region

**Figure 3:**
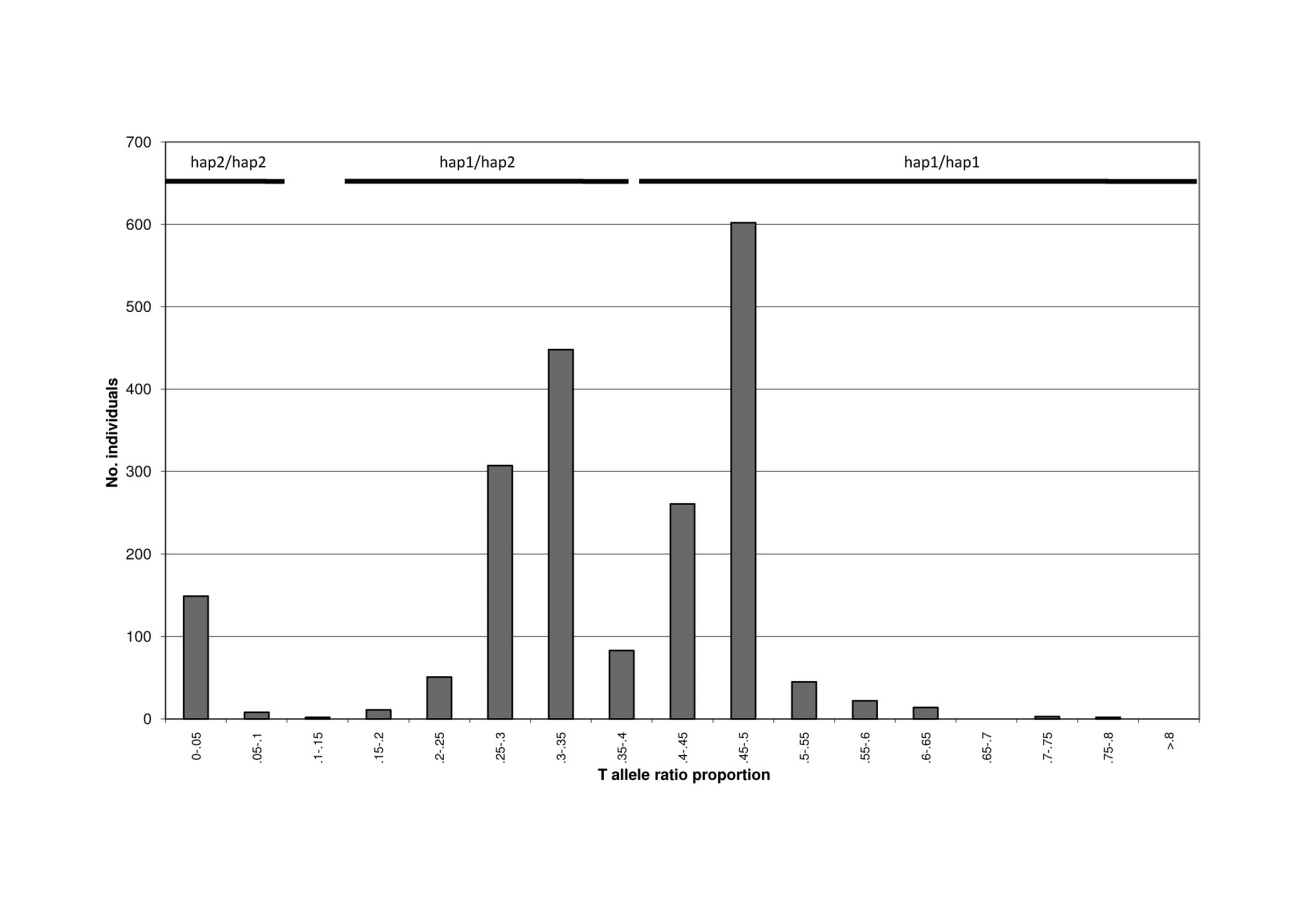
**Typing hap1 and hap2 allelotypes using the Pyrosequencing assay**. The plots show the bins used to call hap1/hap1, hap1/hap2 and hap2/hap2 allelotypes based on C:T ratio frequencies in our whole case and control group. The trimodal distribution corresponding to the three genotypes was evident with ratio bins of 0.05, but finer demarcation between the ratios, especially the hap1/hap2 *versus *hap1/hap1 groups, was possible using bins of 0.01 which showed a minimum frequency of calls at a T allele proportion of 0.38–0.39. The T allele proportion bins used to score allelotypes were therefore: hap2/hap2, 0.0–0.1; hap1/hap2, 0.15–0.37; hap1/hap1, 0.39–1.0. The remaining samples (< 1%) were excluded. There was no significant departure of genotype frequencies from Hardy-Weinberg equilibrium (χ^2^_1 _= 3.55, P = 0.07).

The 68–71 Mb LCR appeared to be one site with a high frequency of mitotic recombination breakpoints in colorectal tumours. However, SNP density is very poor in this region, owing to the exceptionally large number of highly similar paralogous sequences. We therefore developed a new genotyping assay to distinguish between the two major haplotypes that have been described at this site, hap1 of length 2.2 Mb and hap2 of length 1.6 Mb. The *SMN1 *gene is present on both haplotypes as a single copy and *SMN2 *is present as a single copy on haplotype 1 only. Thus, hap1 is *SMN1–SMN2 *and hap2, *SMN1 *only. (Although there have been reports of *SMN1 *and *SMN2 *alleles that have copy numbers different from those found on haplotypes hap1 and hap2 [13], these occur at a very low frequency (< 1%) and their relationships to haplotypes hap1 and hap2 are uncertain; we therefore did not consider the rare *SMN1 *and *SMN2 *copy number alleles further.) We developed a high-throughput genotyping assay based on the C>T difference (rs4916, chr5:69,408,109) in exon 7 that differentiates the *SMN1 *gene from the *SMN2 *gene. In theory, allelotype hap1/hap1 corresponds to an equal dosage of C and T alleles, allelotype hap1/hap2 to a 2:1 C:T ratio and allelotype hap2/hap2 to the presence of the C allele only. Using Pyrosequencing, we initially genotyped a test series of 188 individuals for the two haplotypes. As controls, we used colorectal cancer cell lines, that had known 5q copy number and were homozygous for the 70 Mb repeat region owing to somatic loss of heterozygosity. Although the C:T allele ratios deviated slightly from those expected (with the C allele dosage typically being over-estimated by about 5%), the C:T ratios allowed us to distinguish three genotype groups corresponding to hap1/hap1, hap1/hap2 and hap2/hap2 (Figure 3).

**Figure 4:**
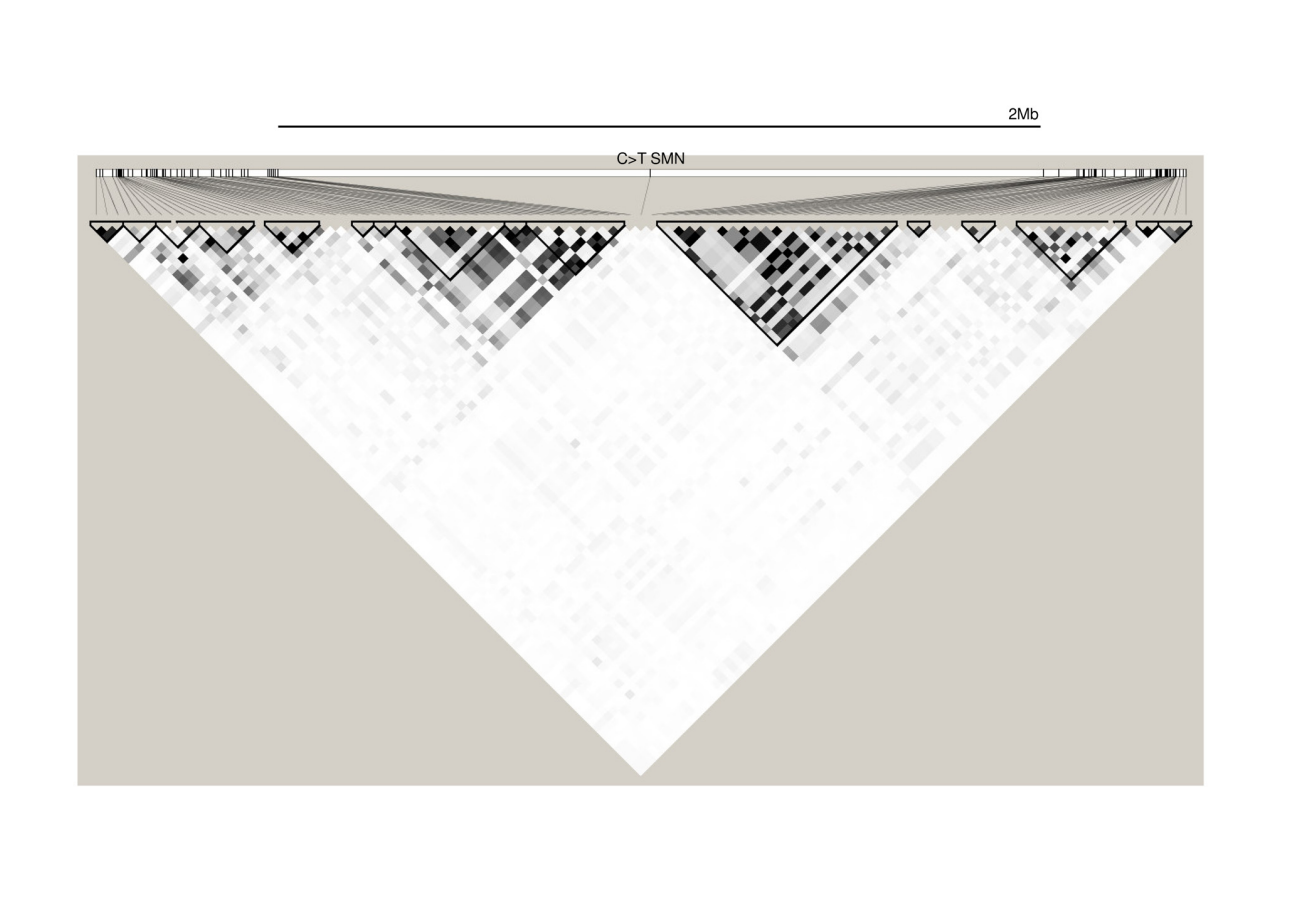
**Haplotype block structure and LD relationships around the 68–71 Mb repeat region**. "CNV" represents the hap1/hap2 polymorphism; other SNPs are from the Illumina Hap550 arrays. The pairwise r^2 ^values are shown, with standard Haploview  shading to represent strength of correlation (darker = stronger).

We initially determined LD between SNPs present on chromosome 5q on the Illumina Hap550 arrays and rs4916 (Figure 4). We found that there was no significant LD between rs4916 and any other SNP in the region from the Hap550 panel. Even the two SNPs on the Hap550 arrays that flanked rs4916 – rs11955686 at chr5: 68,776,409 and rs13168712 at chr5:70,715,406 – were isolated from rs4916 and other SNPs around. Although the absence of LD around the LCR may reflect hyper-recombination within the region or simply normal rates of crossover, it was possible to conclude that the LCR does not grossly suppress *meiotic *recombination.

### Testing regions with high frequencies of mitotic recombination breakpoints for associations with colorectal cancer risk

If mitotic (or meiotic) recombination breakpoints tend to cluster in certain regions, one possible explanation is that the locations of recombination crossovers are selected in normal cells to occur at sites that have no effects on gene function. However, an alternative explanation for recombination breakpoint clustering in mitotic recombination is that the chromosome tends to have an intrinsic fragility at that site. If this latter model were correct, inherited variants near the most common breakpoints might modulate LOH frequencies and hence be associated with differential CRC susceptibility. There is evidence that such variation can modulate meiotic recombination [14,15].

**Figure 5:**
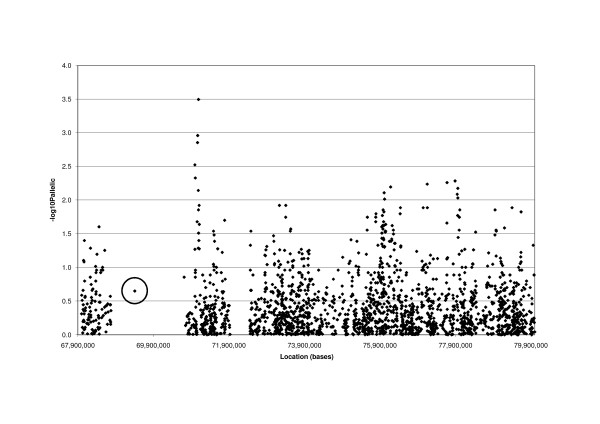
**Associations between SNPs and the LCR polymorphism and colorectal cancer risk**. -log_10_(P) values from the allelic association test (χ^2 ^or Fisher's exact) for the CORGI colorectal tumour cases and controls are shown for each SNP by location, with the LCR copy number polymorphism, rs4916, circled.

We tested whether inherited variation within close to mitotic recombination breakpoints was associated with colorectal cancer risk. Specifically, we determined SNP genotype frequencies from the 65–80 Mb region of chromosome 5 in our primary series of about 1,000 colorectal tumour cases and 1,000 controls. We included all SNPs on the Hap550 arrays (see Additional File 3), plus the following 4 additional SNPs: rs4916; rs2561182, flanking the LCR on the centromeric side; rs28409706, in a small region of sequence with no paralogues within the LCR; and rs7447545, flanking the LCR on the telomeric side. We excluded all SNPs that failed Hardy-Weinberg equilibrium testing at P < 0.01. We then compared genotype frequencies in cases and controls at the remaining 3,843 SNPs using the allelic, recessive and Cochran-Armitage tests. The strongest evidence of association (P_allele test _= 3.2 × 10^-4^, Figure 5) was found at rs2056169 (chr5:71,095,468). This association was suggestive, but did not meet requirements for significance at P = 0.05 after correction for multiple testing. Subsequent analysis in the VICTOR/QUASAR2/1958 series failed to confirm any association (P_allele test _= 0.86). None of the 4 additional SNPs showed any association with colorectal cancer risk under any of the tests of association, with P_allele test _> 0.18 in all cases.

## Discussion

Although maps of meiotic recombination are manifold, very few maps of mitotic recombination exist. We reasoned that mitotic maps might provide not only basic information about a process that is very important in disease, but also data that have more direct clinical benefits. Tumours provide the most convenient means of mapping mitotic recombination events, because the clonal expansion that has occurred means that isolating rare recombinants is not required. It is important, however, to bear in mind the caveats that any findings are derived from a specific cell type and that data can only be gathered from a restricted genomic region, usually between a chromosome centromere and a tumour suppressor gene. The *APC *tumour suppressor provides a particularly good model for study, because multiple tumours are available from FAP patients, LOH generally occurs only in a restricted set of cases, and LOH usually takes the form of mitotic recombination. Although some FAP polyps are polyclonal, we focussed on larger tumours to minimise this problem and found no evidence of 'two-level' LOH to suggest that polyclonality affected our results.

We found that mitotic recombination breakpoints between the centromere (~50 Mb) and the *APC *locus (~112 Mb) were non-random. There was no tendency for breakpoints to occur closer to *APC*, as might be expected were there constraints caused by uncovering recessive lethal alleles or if mitotic recombination were favoured by a longer length of residual chromosome arm that allowed pairing with its homologue for recombination. An increased frequency of breaks close and just distal to the 68–71 Mb LCR region was, however, notable, although the region that showed a greater than expected breakpoint frequency extended as far as about 85 Mb. Does the LCR predispose to chromosome breakage, perhaps as a result of replication fork stalling, and hence to mitotic recombination? Certainly, this is a plausible mechanism, although the maximum breakpoint frequency is actually distal to the LCR and long-range effects must be postulated to explain this finding.

Although very fine mapping of mitotic recombination breakpoints was not possible, it was intriguing to note that the mitotic and meiotic maps were strongly correlated. This finding is consistent with the model in which break induced replication and meiotic recombination are primarily influenced by similar processes, whether mutational or selective in origin. In the past, other groups have failed to find an association between the locations of meiotic and mitotic breakpoints in other genomic regions [7], but recent advances in genomics may have provided better baseline data on which to build our study.

In contrast to the mitotic and meiotic recombination breakpoints, breakpoints involved in copy number changes in colorectal tumours strongly clustered near the chromosome 5 centromere. We suggest that this is the region of greatest chromosomal fragility and that, since most copy number changes occur in aneuploid/polylploid colorectal tumours [12], there is little selective disadvantage in aneusomy for the 5q arm. It is important to bear in mind that chromosome 5q copy number changes generally occur as additional genetic events in malignant tumours, whereas LOH by mitotic recombination is an early event that initiates tumorigenesis. We speculate that breakage through the centromere cannot readily be repaired by mitotic recombination, because the exposure of peri-centromeric repeats produces a chromosome that is prone to non-specific pairing and recombination. Since the selective constraints on *APC *require two mutant copies in the early tumour cell, chromosome breaks must occur between the centromere and *APC*, and hence be repaired by break-induced replication.

LD between the two major CNV alleles in the 70 Mb repeat region and flanking SNPs was extremely low, showing that tagSNPs cannot always be used reliably as proxies for copy number variants [16,17]. Neither the CNV genotype nor any other SNP in the region of maximal mitotic recombination was associated with an increased risk of colorectal cancer. This may be a true reflection of a lack of genetic variation that influences LOH breakpoints, but it must be borne in mind that LOH at *APC *only affects a minority of colorectal tumours and that LOH acts on adenoma formation rather than progression, the consequences being that any effect of differential LOH frequencies on cancer risk may be weak overall.

## Conclusion

In summary, mitotic recombination is an important mutational process that occurs through cross-over repair initiated at non-random 'breakpoints'. The influences on meiotic and mitotic recombination rates seem similar, although they have not yet been elucidated. There is a possible role for LCRs in promoting mitotic recombination, but other selective or mutational factors must also be influential, in some cases perhaps suppressing cross-overs, resulting in few breakpoints close to *APC*. The absence of an association between polymorphisms in regions of frequent mitotic recombination on 5q and colorectal cancer risk suggests that local influences over the rate of loss of heterozygosity at *APC *are not factors that explain inter-individual differences in susceptibility to colorectal cancer.

## Abbreviations

APC: adenomatous polyposis coli; LOH: loss of heterozygosity; CGH: comparative genomic hybridisation; SNP: single nucleotide polymorphism; CRC: colorectal cancer; LCR: low copy number repeat.

## Competing interests

The authors declare that they have no competing interests.

## Authors' contributions

KH, SR, AT, HS, JH, AR, AJ, SS undertook laboratory work. EW and AS undertook bioinformatic and statistical analysis. SS undertook sample preparation and database analysis. SC and TG supplied samples. AS and IT supervised the work. IT planned the experiments, analysed the data and wrote the manuscript. All authors have read and approved the final manuscript.

## Pre-publication history

The pre-publication history for this paper can be accessed here:



## Supplementary Material

Additional file 1 **Fine mapping of mitotic recombination breakpoints close to chr5:68–71 Mb in selected colorectal cancer cell lines**. The table shows the allele sizes (bp) at a number of polymorphic markers on proximal chromosome 5q in 6 colorectal cancer cell lines. Click here for file

Additional file 2 **Chromosome 5 SNPs used to assess LOH on the custom Illumina Goldengate arrays**. SNP IDs and location are shown. Click here for file

Additional file 3 **Chromosome 5 SNPs between 68 Mb and 80 Mb tested for association with colorectal cancer risk**. The table shows genotype counts (nominally AA, AB, BB) in cases and controls, followed by allelic odds ratio, and P value under the allelic χ^2 ^test (or Fisher's exact test for low cell counts). Click here for file
