# Supplementary material for: A mitotic recombination map proximal to the APC locus on chromosome 5q and assessment of influences on colorectal cancer risk
Source: BMC Med Genet. 2009 Jun 10;10:54. doi: 10.1186/1471-2350-10-54 (PMC2705358; doi:10.1186/1471-2350-10-54)
Supplement: Additional file 1 — Fine mapping of mitotic recombination breakpoints close to chr5:68–71 Mb in selected colorectal cancer cell lines. The table shows the allele sizes (bp) at a number of polymorphic markers on proximal chromosome 5q in 6 colorectal cancer cell lines. [file 1471-2350-10-54-S1.pdf]

Additional file 1. Fine mapping of mitotic recombination breakpoints close to chr5:68-71Mb in selected colorectal cancer cell lines.
The Table shows the allele sizes (bp) at a number of polymorphic markers on proximal chromosome 5q in 6 colorectal cancer cell lines.
Alleles of different sizes indicate heterozygosity.
C32 and SW837 are controls without LOH.
Grey shading indicates the distal limit of the region that has retention of heterozygosity.
The actual mitotic recombination breakpoint lies distal to the shaded region, but in the absence of paired constitutional DNA, the breakpoint location remains imprecise.

Position	Polymorphism ID 1	Polymorphism ID 2	VACO4S		C10		CAC02		C70		C32		SW837		Allele sizes (bp)	Heterozygosity (where known)
52,321,211	AFM207YG11	D5S623	145	147	136	136	147	147	139	143	147	147	147	153	143-159	
55,009,278	AFM311YD1	D5S664	127	129	131	133	120	131	129	135	112	128	116	127	119-143	0.84
56,030,500	AFM163XA11	D5S407	101	111	103	109	113	119	115	117			101	115	107-127	0.87
57,604,744	AFM095ZB7	D5S398	109	114	109	111	111	111	103	107			103	107	109-121	
58,185,562	AFMA081TG1	D5S2107	168	172	176	180	172	174	174	174	174	174	170	174	166-188	0.85
60,485,524	AFM207YH2	D5S624	140	154	144	144	140	154	144	152	152	158	156	158	146-166	0.84
61,010,830	AFMA247YH9	D5S1990	216	240	216	244	216	260	222	238	236	242	234	236	204-248	0.86
65,979,461	AFMA052TB9	D5S2089	177	181	163	171	163	163	163	177	173	177	163	163	163-185	
66,282,906	AFM292VE1	D5S647	130	132	146	150	136	152	130	150	134	134	130	132	126-156	0.82
67,548,348	rs2067135		158	186	160	186	158	160	158	188	186	186	158	186	ins/del 28bp	0.50
67,839,171	AFMB201YB9	D5S2019	110	110	106	110	112	114	108	108					98-120	
67,774,622	rs1305058		124	143	124	143	124	124	143	143	145	145	124	143	ins/del 18bp	0.49
68,506,356	rs3087334		162	165	166	166	166	166	162	162	162	165	162	162	ins/del 4bp	0.43
70,864,185	rs2307799		117	121	121	121	121	121	117	117	121	121	117	121	ins/del 4bp	0.49
71,865,418	rs1610940		173	178	178	178	178	178	178	178	174	178	174	178	ins/del 4bp	0.43
74,563,430	AFMA348WD1	D5S2003	185	185	173	173	183	183	183	183	173	187	171	187	157-193	0.81
76,709,492	AFMB330YD9	D5S2041	230	230	218	218	228	228	219	219	224	226	224	224	206-234	0.81
81,493,027	AFMB307YD5	D5S2029	130	130	128	128	140	140	122	122	120	128	120	138	122-148	0.88
83,635,324	MFD27A	D5S107	141	141	141	141	141	141	143	143	139	141	131	139	133-155	0.81
95,838,450	AFM288VA9	D5S644	89	89	87	87	91	91	77	77	85	91	85	87	81-101	0.85
104,002,962	AFM321YB5	D5S669	202	202	198	198	202	202	196	196	202	202	196	198	190-210	0.81
112,250,000	D5S346		119	119	111	111			117	117	111	117	107	109	96-122	0.82
