# Supplementary material for: A mitotic recombination map proximal to the APC locus on chromosome 5q and assessment of influences on colorectal cancer risk
Source: BMC Med Genet. 2009 Jun 10;10:54. doi: 10.1186/1471-2350-10-54 (PMC2705358; doi:10.1186/1471-2350-10-54)
Supplement: Additional file 2 — Chromosome 5 SNPs used to assess LOH on the custom Illumina Goldengate arrays. SNP IDs and location are shown. [file 1471-2350-10-54-S2.pdf]

Additional file 2. Chromosome 5 SNPs used to assess LOH on the custom Illumina Goldengate arrays.
SNP IDs and location are shown.

Name	Chr	Position (bases)
rs3828570	5	654,647
rs413666	5	726,408
rs465498	5	1,378,803
rs27070	5	1,399,303
rs904759	5	1,789,837
rs1373942	5	13,709,297
rs2034586	5	30,082,244
rs1328254	5	49,596,616
rs496230	5	49,606,770
rs1033109	5	49,659,296
rs2102454	5	49,784,037
rs1423644	5	49,867,027
rs204915	5	50,062,685
rs27863	5	50,201,246
rs4866023	5	50,381,012
rs1501976	5	50,540,380
rs6867206	5	50,688,451
rs6880356	5	50,839,649
rs3857260	5	51,052,663
rs9291922	5	51,266,644
rs7341064	5	51,469,038
rs1424903	5	51,669,550
rs1834261	5	51,788,163
rs11958309	5	51,930,917
rs1820167	5	52,155,113
rs2974987	5	52,386,999
rs977576	5	52,592,967
rs1363969	5	52,807,274
rs256094	5	53,008,681
rs7713737	5	53,241,460
rs700915	5	53,456,391
rs10075673	5	53,657,681
rs2059216	5	53,854,270
rs895329	5	54,068,584
rs952382	5	54,263,361
rs336081	5	54,469,150
rs2290597	5	54,685,878
rs7721054	5	54,926,559
rs2619046	5	55,133,291
rs6870870	5	55,330,085
rs191205	5	55,437,355
rs1020388	5	55,595,784
rs157843	5	55,834,941
rs10060816	5	56,016,407

rs832569	5	56,190,341
rs831818	5	56,393,904
rs7168	5	56,594,995
rs6450425	5	56,785,344
rs2109479	5	56,979,996
rs1020661	5	57,215,261
rs7731718	5	57,409,658
rs40207	5	57,611,462
rs697141	5	57,791,740
rs158958	5	57,992,021
rs12655408	5	58,214,110
rs1391651	5	58,412,212
rs256752	5	58,633,646
rs40215	5	58,877,103
rs2164661	5	59,085,334
rs7715428	5	59,279,140
rs1981848	5	59,478,693
rs12054887	5	59,627,881
rs153031	5	59,823,048
rs3857236	5	59,945,286
rs6874699	5	60,109,757
rs34638	5	60,386,244
rs34609	5	60,517,165
rs6449531	5	60,747,969
rs1501841	5	60,983,240
rs1374065	5	61,099,359
rs2032876	5	61,344,489
rs13359224	5	61,518,850
rs959899	5	61,687,005
rs26642	5	61,820,146
rs2112984	5	61,975,517
rs2363295	5	62,168,416
rs2221988	5	62,372,925
rs346416	5	62,577,650
rs10038844	5	62,745,709
rs1559090	5	62,910,088
rs13173277	5	63,073,293
rs358524	5	63,258,537
rs356578	5	63,477,556
rs10064715	5	63,701,617
rs10067169	5	63,906,866
rs2034995	5	64,123,568
rs2163955	5	64,277,699
rs264739	5	64,401,809
rs16893588	5	64,583,755
rs1493451	5	64,762,196
rs27141	5	64,917,692
rs1301475	5	65,093,399
rs251608	5	65,283,649
rs153389	5	65,458,485
rs1896654	5	65,623,540
rs10514995	5	65,775,195

rs4128347	5	65,943,943
rs33724	5	66,134,163
rs27217	5	66,254,873
rs2545387	5	66,424,155
rs1428478	5	66,573,467
rs32015	5	66,733,395
rs4267824	5	66,914,243
rs6449915	5	67,079,671
rs9291908	5	67,275,992
rs993863	5	67,459,612
rs3756668	5	67,631,844
rs6897790	5	67,838,687
rs724006	5	67,878,862
rs33294	5	68,106,034
rs249233	5	68,125,520
rs7720417	5	68,194,356
rs6449986	5	68,270,525
rs6898541	5	68,285,570
rs7733467	5	68,300,509
rs4976058	5	68,326,778
rs4976067	5	68,352,124
rs164561	5	68,376,084
rs163192	5	68,415,592
rs100192	5	68,523,930
rs2930946	5	68,548,760
rs2972381	5	68,573,998
rs10940209	5	68,594,306
rs4421064	5	68,614,184
rs4976189	5	68,634,595
rs2010352	5	68,692,083
rs1168402	5	68,743,128
rs2561182	5	68,769,401
rs28489109	5	68,844,257
rs4081993	5	68,891,872
rs28665832	5	69,274,432
rs2591321	5	69,321,642
rs2630908	5	69,734,045
rs575909	5	70,309,633
rs28751879	5	70,341,309
rs28538463	5	70,341,452
rs28447466	5	70,342,434
rs36065930	5	70,343,142
rs4976210	5	70,343,220
rs28409706	5	70,344,007
rs6453529	5	70,739,833
rs7449303	5	70,745,215
rs7443752	5	70,752,311
rs12520305	5	70,763,700
rs986217	5	70,780,414
rs7448990	5	70,789,690
rs4337836	5	70,817,174
rs1961760	5	70,842,405

rs182190	5	70,875,989
rs277966	5	70,915,766
rs277984	5	70,966,722
rs3763154	5	71,049,233
rs1798575	5	71,141,418
rs10058537	5	71,209,059
rs7719321	5	71,309,216
rs1217791	5	71,406,940
rs6874186	5	71,512,871
rs11749445	5	71,603,661
rs10069831	5	71,780,124
rs33419	5	71,882,477
rs3846640	5	72,055,722
rs198214	5	72,255,507
rs1200485	5	72,420,814
rs9293560	5	72,637,254
rs7703551	5	72,842,274
rs6890324	5	73,065,896
rs283596	5	73,278,467
rs158812	5	73,526,030
rs586853	5	73,714,140
rs2927633	5	73,816,540
rs379146	5	73,968,290
rs1993370	5	74,194,893
rs4492095	5	74,353,035
rs1363577	5	74,498,941
rs3843480	5	74,660,238
rs4704223	5	74,809,176
rs1863934	5	74,952,628
rs39656	5	75,084,221
rs11746298	5	75,290,322
rs7713316	5	75,491,776
rs890708	5	75,719,916
rs1047530	5	75,947,155
rs2242995	5	76,151,421
rs1500	5	76,312,594
rs6864250	5	76,485,946
rs1531615	5	76,654,613
rs10076883	5	76,871,991
rs456187	5	77,087,709
rs837030	5	77,269,764
rs1363235	5	77,456,412
rs10805919	5	77,628,680
rs1050674	5	77,818,845
rs340085	5	78,034,542
rs337887	5	78,264,792
rs649514	5	78,445,289
rs2220839	5	78,639,266
rs736201	5	78,873,312
rs6870619	5	79,031,806
rs265002	5	79,194,908
rs2434291	5	79,433,983

rs10063054	5	79,680,444
rs6883095	5	79,926,803
rs6151850	5	80,124,816
rs34999	5	80,312,801
rs1020720	5	80,508,524
rs4703517	5	80,691,022
rs7702047	5	80,890,151
rs417304	5	81,068,575
rs1566629	5	81,243,028
rs6891113	5	81,428,874
rs2215128	5	81,630,840
rs1501656	5	81,822,452
rs11948759	5	82,078,918
rs27942	5	82,330,448
rs1120476	5	82,458,399
rs10805813	5	82,683,802
rs173686	5	82,847,256
rs336965	5	83,004,341
rs4563604	5	83,178,877
rs958643	5	83,343,343
rs164438	5	83,477,060
rs1445748	5	83,616,059
rs16901127	5	83,836,023
rs10076429	5	84,039,445
rs7719527	5	84,245,129
rs4565249	5	84,439,840
rs733481	5	84,646,369
rs413927	5	84,848,924
rs7718884	5	85,051,201
rs893554	5	85,267,959
rs1006163	5	85,517,198
rs7735255	5	85,682,856
rs6452716	5	85,875,334
rs187609	5	86,049,616
rs4920774	5	86,229,784
rs2112168	5	86,440,646
rs13362486	5	86,639,581
rs878196	5	86,830,251
rs840818	5	87,012,486
rs710363	5	87,176,203
rs6879443	5	87,357,550
rs357509	5	87,523,698
rs247913	5	87,654,484
rs4352629	5	87,792,577
rs616391	5	88,074,601
rs1455098	5	88,289,093
rs10474292	5	88,516,971
rs383242	5	88,764,803
rs10057743	5	88,977,154
rs10063565	5	89,168,121
rs411198	5	89,378,930
rs4916794	5	89,609,684

rs2935530	5	89,721,016
rs37238	5	89,860,732
rs10070074	5	90,058,163
rs1976566	5	90,253,603
rs4458614	5	90,476,902
rs6452949	5	90,680,893
rs1505851	5	90,929,710
rs1366133	5	91,150,340
rs825395	5	91,284,916
rs10056713	5	91,438,628
rs4395616	5	91,720,842
rs12234024	5	91,938,709
rs1429056	5	92,174,756
rs1390614	5	92,420,334
rs7443364	5	92,680,888
rs2029401	5	92,916,785
rs11135392	5	93,159,478
rs6860390	5	93,520,487
rs29963	5	93,723,401
rs11949638	5	93,933,645
rs4869118	5	94,114,614
rs293033	5	94,307,424
rs251080	5	94,537,060
rs2431503	5	94,746,042
rs2560258	5	94,869,243
rs440342	5	95,003,264
rs34896	5	95,109,887
rs10515232	5	95,336,741
rs10476546	5	95,578,352
rs4869282	5	95,695,921
rs269853	5	95,832,735
rs26515	5	96,080,593
rs1981846	5	96,294,618
rs444243	5	96,493,512
rs1919247	5	96,681,502
rs1843331	5	96,873,334
rs1560327	5	97,053,020
rs6557008	5	97,192,546
rs380331	5	97,371,076
rs11951090	5	97,569,655
rs725763	5	97,720,491
rs27022	5	97,968,940
rs1508792	5	98,137,048
rs162149	5	98,277,455
rs7717324	5	98,426,572
rs1073601	5	98,594,496
rs2030605	5	98,741,476
rs2511961	5	98,906,572
rs13354756	5	99,057,674
rs1462405	5	99,102,213
rs1622879	5	99,138,247
rs6594769	5	99,176,463

rs7448852	5	99,214,153
rs2927424	5	99,286,029
rs1542520	5	99,376,285
rs7730463	5	99,406,397
rs9790951	5	99,437,789
rs4317298	5	99,493,434
rs6894070	5	99,553,754
rs1504524	5	99,579,920
rs445427	5	99,617,942
rs1833843	5	99,655,735
rs1880076	5	99,711,180
rs4703100	5	99,812,015
rs27458	5	99,908,670
rs157178	5	100,099,693
rs3756355	5	100,267,900
rs6595881	5	100,701,152
rs4703156	5	100,935,754
rs10463906	5	101,174,950
rs1477616	5	101,423,290
rs841921	5	101,658,829
rs7735587	5	101,906,139
rs461605	5	102,148,168
rs17154821	5	102,269,240
rs154355	5	102,526,780
rs372564	5	102,678,423
rs10066920	5	102,794,751
rs414926	5	102,943,933
rs9327892	5	103,121,479
rs10051527	5	103,311,866
rs1922495	5	103,506,417
rs6596572	5	103,752,969
rs254023	5	103,983,260
rs1217470	5	104,237,855
rs294148	5	104,489,057
rs249557	5	104,710,326
rs12518325	5	104,979,858
rs10447297	5	105,246,505
rs1435199	5	105,506,390
rs4279324	5	105,748,916
rs6596684	5	105,972,832
rs1898625	5	106,171,984
rs4395620	5	106,356,225
rs7735512	5	106,551,753
rs252816	5	106,752,310
rs4090594	5	106,984,342
rs1368441	5	107,223,156
rs286821	5	107,457,409
rs2966833	5	107,699,831
rs7719573	5	107,960,054
rs6880284	5	108,196,054
rs17161607	5	108,470,727
rs1045706	5	108,742,197

rs77013	5	108,990,985
rs1807912	5	109,245,567
rs6883748	5	109,441,142
rs6895504	5	109,640,667
rs6892291	5	109,866,948
rs244431	5	110,133,062
rs6879236	5	110,361,050
rs7714962	5	110,587,453
rs2300782	5	110,816,684
rs1154308	5	111,049,878
rs1507844	5	111,275,761
rs27342	5	111,493,331
rs1464765	5	111,639,361
rs341328	5	111,855,488
rs1968557	5	112,056,943
rs1460039	5	122,078,905
rs708459	5	132,236,067
rs33388	5	142,677,488
rs1438952	5	152,448,638
rs1299048	5	162,160,289
rs1445795	5	172,772,594
rs1544926	5	177,597,948
rs584609	5	178,774,015
rs1982301	5	179,482,982
rs1467	5	180,418,326
rs1053110	5	180,420,866
