# Supplementary material for: A mitotic recombination map proximal to the APC locus on chromosome 5q and assessment of influences on colorectal cancer risk
Source: BMC Med Genet. 2009 Jun 10;10:54. doi: 10.1186/1471-2350-10-54 (PMC2705358; doi:10.1186/1471-2350-10-54)
Supplement: Additional file 3 — Chromosome 5 SNPs between 68 Mb and 80 Mb tested for association with colorectal cancer risk. The table shows genotype counts (nominally AA, AB, BB) in cases and controls, followed by allelic odds ratio, and P value under the allelic χ2 test (or Fisher's exact test for low cell counts). [file 1471-2350-10-54-S3.pdf]

Additional file 3. Chromosome 5 SNPs between 68Mb and 80Mb tested for association with colorectal cancer risk.

The table shows genotype counts (nominally AA, AB, BB) in cases and controls, followed by allelic odds ratio, and P value under the allelic χ2 test (or Fisher's exact test for low cell counts).

SNP	chr	loc	AAcases	ABcases	BBcases	AActrls	ABctrls	BBctrls	all_OR	all_pval	$-\log_{10}P_{allelic}$
rs13171512	chr5	68,000,787	617	277	28	615	280	37	1.06	4.90E-01	3.10E-01
rs10940172	chr5	68,002,670	584	304	34	576	307	49	1.1	2.60E-01	5.85E-01
rs2607378	chr5	68,015,556	1	61	853	2	72	843	0.825	3.10E-01	5.09E-01
rs10070379	chr5	68,022,050	607	288	26	629	268	34	0.973	7.80E-01	1.08E-01
rs1286371	chr5	68,029,954	517	351	54	526	345	61	1.01	9.40E-01	2.69E-02
rs12658051	chr5	68,039,213	407	408	106	437	399	96	0.915	2.20E-01	6.58E-01
rs1687503	chr5	68,039,523	27	214	681	14	232	686	1.05	6.40E-01	1.94E-01
rs246978	chr5	68,041,802	474	364	80	461	385	85	1.06	4.20E-01	3.77E-01
rs246973	chr5	68,043,559	78	369	475	85	386	459	0.934	3.60E-01	4.44E-01
rs3852133	chr5	68,052,116	28	218	668	18	204	706	1.19	7.90E-02	1.10E+00
rs35243	chr5	68,054,018	63	356	503	69	364	499	0.96	6.10E-01	2.15E-01
rs35240	chr5	68,055,530	99	411	412	110	419	403	0.945	4.40E-01	3.57E-01
rs35239	chr5	68,064,834	43	281	598	32	313	587	0.98	8.40E-01	7.57E-02
rs10515095	chr5	68,065,227	670	223	29	704	210	18	0.846	8.30E-02	1.08E+00
rs33278	chr5	68,065,763	582	294	45	575	314	40	1.02	8.20E-01	8.62E-02
rs27687	chr5	68,068,178	499	353	70	492	362	78	1.05	4.90E-01	3.10E-01
rs26685	chr5	68,068,766	212	446	264	206	452	274	1.03	6.40E-01	1.94E-01
rs12516844	chr5	68,073,833	770	144	8	740	184	8	1.27	4.00E-02	1.40E+00
rs7703932	chr5	68,080,287	214	428	275	226	457	244	0.91	1.60E-01	7.96E-01
rs13177007	chr5	68,080,610	763	155	4	777	150	5	0.968	8.30E-01	8.09E-02
rs26337	chr5	68,090,330	156	408	358	137	421	374	1.08	2.80E-01	5.53E-01
rs13175238	chr5	68,094,416	5	151	766	5	147	780	1.04	7.80E-01	1.08E-01
rs28289	chr5	68,096,315	247	453	222	276	440	216	0.928	2.70E-01	5.69E-01
rs2451819	chr5	68,100,520	323	422	175	330	446	156	0.948	4.40E-01	3.57E-01
rs33294	chr5	68,106,034	308	430	184	321	442	169	0.943	4.00E-01	3.98E-01
rs2913277	chr5	68,138,961	184	423	315	181	451	300	0.971	6.80E-01	1.67E-01
rs2913280	chr5	68,149,933	85	358	479	85	391	456	0.932	3.50E-01	4.56E-01
rs11959994	chr5	68,156,251	17	197	694	14	206	701	1	9.70E-01	1.32E-02
rs2972426	chr5	68,166,067	448	380	94	444	386	102	1.04	6.00E-01	2.22E-01
rs1427906	chr5	68,166,377	93	380	449	101	383	448	0.968	6.80E-01	1.67E-01
rs10039512	chr5	68,178,949	446	383	93	446	385	101	1.03	7.00E-01	1.55E-01
rs7710526	chr5	68,192,781	921	1	0	932	0	0	0	5.00E-01	3.01E-01
rs7720417	chr5	68,194,356	91	383	448	100	387	445	0.961	6.00E-01	2.22E-01
rs12153013	chr5	68,194,574	93	382	447	101	390	441	0.956	5.50E-01	2.60E-01
rs4246760	chr5	68,214,163	192	454	276	177	466	289	1.06	3.90E-01	4.09E-01
rs4131295	chr5	68,230,400	796	118	8	822	108	2	0.816	1.40E-01	8.54E-01
rs7713600	chr5	68,235,434	29	259	634	39	261	632	0.934	4.50E-01	3.47E-01
rs7718291	chr5	68,236,398	601	285	36	640	270	22	0.844	5.20E-02	1.28E+00
rs10038257	chr5	68,242,153	30	258	634	39	260	633	0.941	5.10E-01	2.92E-01
rs10940184	chr5	68,245,085	736	174	12	751	174	7	0.932	5.50E-01	2.60E-01
rs11747940	chr5	68,245,160	385	416	121	408	397	127	0.967	6.50E-01	1.87E-01
rs13436110	chr5	68,253,745	624	265	33	604	287	40	1.12	1.80E-01	7.45E-01
rs11951039	chr5	68,257,343	637	249	30	656	255	18	0.915	3.40E-01	4.69E-01
rs4976054	chr5	68,260,759	438	412	72	464	405	63	0.923	2.80E-01	5.53E-01
rs6449986	chr5	68,270,525	298	458	166	311	446	175	0.994	9.60E-01	1.77E-02
rs4505911	chr5	68,277,393	8	130	784	4	119	809	1.18	2.20E-01	6.58E-01
rs10064315	chr5	68,281,215	196	459	267	183	487	262	1.02	8.40E-01	7.57E-02
rs10071585	chr5	68,281,676	343	439	132	334	442	151	1.07	3.10E-01	5.09E-01
rs11951431	chr5	68,289,708	504	340	78	488	385	59	1	9.80E-01	8.77E-03
rs6414814	chr5	68,299,358	135	448	338	159	447	326	0.918	2.10E-01	6.78E-01
rs4246761	chr5	68,315,467	469	370	82	464	381	87	1.04	6.30E-01	2.01E-01
rs4976058	chr5	68,326,778	34	294	594	32	298	602	1.01	9.00E-01	4.58E-02
rs3922937	chr5	68,333,747	612	278	32	616	287	28	0.992	9.60E-01	1.77E-02
rs4976075	chr5	68,357,003	305	448	169	288	461	182	1.07	3.20E-01	4.95E-01
rs11741847	chr5	68,367,828	84	352	486	69	393	469	0.984	8.60E-01	6.55E-02
rs52131	chr5	68,371,497	669	235	18	704	218	10	0.85	9.70E-02	1.01E+00
rs1982773	chr5	68,371,892	693	198	31	690	225	17	0.983	8.90E-01	5.06E-02
rs11738928	chr5	68,374,875	0	33	889	1	45	886	0.704	1.60E-01	7.96E-01
rs164561	chr5	68,376,084	505	373	44	494	388	50	1.06	4.30E-01	3.67E-01
rs4976178	chr5	68,377,991	424	400	98	412	403	117	1.09	2.50E-01	6.02E-01
rs149292	chr5	68,381,404	567	310	45	554	336	41	1.04	6.10E-01	2.15E-01
rs164562	chr5	68,381,681	18	235	669	11	218	702	1.16	1.20E-01	9.21E-01
rs6897246	chr5	68,385,673	98	401	422	117	406	409	0.917	2.30E-01	6.38E-01

	rs2637130	chr5	68,397,214	270	457	195	306	446	180	0.897	1.10E-01	9.59E-01		
	rs2434349	chr5	68,407,652	243	463	214	285	449	198	0.883	6.40E-02	1.19E+00		
	rs12522805	chr5	68,432,336	58	324	535	56	326	548	1.02	7.80E-01	1.08E-01		
	rs164396	chr5	68,443,472	177	415	327	168	461	299	0.956	5.20E-01	2.84E-01		
	rs164392	chr5	68,445,370	560	307	55	554	341	37	0.98	8.30E-01	8.09E-02		
	rs164578	chr5	68,453,399	179	402	326	168	458	299	0.959	5.50E-01	2.60E-01		
	rs6880864	chr5	68,458,441	601	244	25	626	251	19	0.946	5.70E-01	2.44E-01		
	rs337253	chr5	68,458,825	326	416	180	300	466	166	1.03	6.80E-01	1.67E-01		
	rs435395	chr5	68,462,731	257	430	198	235	464	184	1.02	8.20E-01	8.62E-02		
	rs11749991	chr5	68,462,828	26	236	660	11	220	701	1.24	2.50E-02	1.60E+00		
	rs11951414	chr5	68,467,628	46	341	535	47	344	541	0.999	9.80E-01	8.77E-03		
	rs338685	chr5	68,470,725	84	375	461	87	404	440	0.93	3.30E-01	4.81E-01		
	rs4976175	chr5	68,484,450	18	159	745	12	196	724	0.884	2.60E-01	5.85E-01		
	rs2450246	chr5	68,486,504	238	460	224	232	473	227	1.02	7.90E-01	1.02E-01		
	rs389686	chr5	68,490,922	123	409	390	126	440	366	0.933	3.30E-01	4.81E-01		
	rs3846484	chr5	68,504,814	704	199	19	736	184	12	0.852	1.20E-01	9.21E-01		
	rs7736312	chr5	68,521,747	704	199	19	737	183	12	0.847	1.10E-01	9.59E-01		
	rs1976101	chr5	68,532,208	440	389	93	421	423	88	1.05	5.60E-01	2.52E-01		
	rs676012	chr5	68,540,723	238	475	209	255	477	200	0.946	4.20E-01	3.77E-01		
	rs11744596	chr5	68,555,047	345	436	138	314	464	154	1.12	1.00E-01	1.00E+00		
	rs2932777	chr5	68,560,783	138	437	347	152	466	314	0.896	1.10E-01	9.59E-01		
	rs2972381	chr5	68,573,998	210	466	236	202	476	249	1.05	5.20E-01	2.84E-01		
	rs4077460	chr5	68,608,424	251	473	198	272	469	191	0.943	3.90E-01	4.09E-01		
	rs12651858	chr5	68,613,223	791	124	7	768	155	9	1.26	5.60E-02	1.25E+00		
	rs6450029	chr5	68,632,059	212	464	246	200	474	257	1.05	4.80E-01	3.19E-01		
	rs11749723	chr5	68,645,641	176	442	304	170	467	295	0.99	9.10E-01	4.10E-02		
	rs10471774	chr5	68,652,087	208	462	252	198	463	271	1.06	3.70E-01	4.32E-01		
	rs12187268	chr5	68,661,542	261	456	204	277	464	191	0.941	3.70E-01	4.32E-01		
	rs9291949	chr5	68,684,526	204	442	275	205	463	264	0.973	7.00E-01	1.55E-01		
	rs6450035	chr5	68,691,891	3	103	816	5	115	812	0.874	3.50E-01	4.56E-01		
	rs3756399	chr5	68,701,565	2	86	834	2	98	832	0.886	4.60E-01	3.37E-01		
	rs1185246	chr5	68,751,066	204	444	274	206	466	260	0.965	6.10E-01	2.15E-01		
	rs299098	chr5	68,755,507	230	446	199	247	459	187	0.938	3.60E-01	4.44E-01		
	rs6450041	chr5	68,760,674	1	84	836	2	99	831	0.837	2.70E-01	5.69E-01		
	rs299075	chr5	68,764,300	221	457	241	203	475	252	1.06	3.60E-01	4.44E-01		
	rs2561182	chr5	68,769,401	202	444	271	193	474	247		6.48E-01	1.88E-01		
	rs2561183	chr5	68,769,454	206	443	273	205	466	260	0.973	7.00E-01	1.55E-01		
	rs11955686	chr5	68,776,409	1	53	868	1	63	868	0.851	4.40E-01	3.57E-01		
	rs4916 (CNV)	chr5	69,408,109	560	526	90	870	708	134		2.25E-01	6.48E-01		
	rs13168712	chr5	70,715,382	388	418	112	361	443	126	1.11	1.40E-01	8.54E-01		
	rs7443752	chr5	70,752,311	429	395	92	417	419	88	1.03	7.30E-01	1.37E-01		
	rs7447545	chr5	70,783,326	251	470	195	265	459	195		6.72E-01	1.73E-01		
	rs6452722	chr5	70,798,815	657	239	26	651	253	28	1.06	5.40E-01	2.68E-01		
	rs7728577	chr5	70,803,025	26	237	658	27	254	651	0.94	5.20E-01	2.84E-01		
	rs6452796	chr5	70,816,345	705	203	11	721	190	18	0.993	9.80E-01	8.77E-03		
	rs7444322	chr5	70,817,510	26	237	659	28	251	652	0.941	5.30E-01	2.76E-01		
	rs4704050	chr5	70,829,327	397	419	106	399	422	109	1.01	9.30E-01	3.15E-02		
	rs3761966	chr5	70,836,231	105	419	398	109	423	399	0.986	8.70E-01	6.05E-02		
	rs3761967	chr5	70,836,294	227	470	225	228	456	248	1.05	4.90E-01	3.10E-01		
	rs715748	chr5	70,842,213	227	470	224	228	455	247	1.05	4.90E-01	3.10E-01		
	rs421128	chr5	70,842,829	105	416	397	109	421	398	0.985	8.60E-01	6.55E-02		
	rs3748041	chr5	70,844,925	253	471	198	274	454	204	0.969	6.60E-01	1.80E-01		
	rs279322	chr5	70,847,577	252	472	198	274	456	202	0.963	5.90E-01	2.29E-01		
	rs277970	chr5	70,863,731	7	138	777	9	135	788	1	9.80E-01	8.77E-03		
	rs276590	chr5	70,870,589	228	470	224	228	457	247	1.05	4.70E-01	3.28E-01		
	rs277941	chr5	70,893,950	105	418	399	109	423	397	0.981	8.00E-01	9.69E-02		
	rs464155	chr5	70,920,791	223	471	228	246	457	229	0.954	4.90E-01	3.10E-01		
	rs12153571	chr5	70,921,729	611	277	34	610	286	36	1.03	7.30E-01	1.37E-01		
	rs277952	chr5	70,948,437	130	431	361	130	437	365	1	9.90E-01	4.36E-03		
	rs12516456	chr5	70,958,372	664	234	24	660	250	22	1.04	7.20E-01	1.43E-01		
	rs277980	chr5	70,963,612	199	467	256	205	452	275	1.03	7.10E-01	1.49E-01		
	rs7444180	chr5	70,967,758	576	271	29	571	288	30	1.05	5.80E-01	2.37E-01		
	rs2242372	chr5	70,984,410	685	222	15	700	216	16	0.97	7.80E-01	1.08E-01		
	rs17356585	chr5	70,999,567	772	145	5	826	103	3	0.677	3.00E-03	2.52E+00		
	rs277921	chr5	71,000,819	425	411	86	470	387	73	0.869	5.40E-02	1.27E+00		
	rs539449	chr5	71,002,933	137	449	335	127	444	361	1.08	2.70E-01	5.69E-01		
	rs281134	chr5	71,008,018	145	417	360	131	437	364	1.04	6.20E-01	2.08E-01		
	rs11949150	chr5	71,010,229	4	141	777	6	145	781	0.956	7.50E-01	1.25E-01		
	rs281126	chr5	71,011,623	9	208	705	10	179	743	1.17	1.40E-01	8.54E-01		
	rs17356884	chr5	71,011,814	701	199	22	676	232	24	1.16	1.20E-01	9.21E-01		

	rs16870827	chr5	71,012,259	630	260	32	581	305	46	1.27	4.70E-03	2.33E+00		
	rs281124	chr5	71,013,753	200	472	250	228	476	228	0.897	1.10E-01	9.59E-01		
	rs281121	chr5	71,021,778	843	77	2	840	90	2	1.16	3.90E-01	4.09E-01		
	rs2115031	chr5	71,022,231	171	430	321	160	451	321	1.02	7.80E-01	1.08E-01		
	rs172182	chr5	71,024,374	320	447	154	342	427	163	0.976	7.40E-01	1.31E-01		
	rs7703100	chr5	71,026,670	11	172	739	12	169	751	1.02	9.10E-01	4.10E-02		
	rs2560665	chr5	71,029,670	155	447	320	161	428	343	1.03	6.40E-01	1.94E-01		
	rs11741424	chr5	71,033,221	314	452	156	334	432	166	0.982	8.10E-01	9.15E-02		
	rs6872120	chr5	71,033,905	272	464	186	285	447	200	1	9.80E-01	8.77E-03		
	rs6453124	chr5	71,036,822	691	215	15	697	213	22	1.04	7.00E-01	1.55E-01		
	rs4704168	chr5	71,042,463	312	441	151	332	427	162	0.987	8.70E-01	6.05E-02		
	rs10515116	chr5	71,046,420	11	174	737	12	171	749	1.02	9.10E-01	4.10E-02		
	rs6859438	chr5	71,049,222	1	49	872	2	59	871	0.813	3.20E-01	4.95E-01		
	rs3763154	chr5	71,049,233	297	454	171	308	445	179	0.996	9.80E-01	8.77E-03		
	rs3763153	chr5	71,049,403	443	375	103	443	390	98	0.997	9.90E-01	4.36E-03		
	rs3857384	chr5	71,049,720	13	188	721	16	187	729	0.986	9.30E-01	3.15E-02		
	rs6894758	chr5	71,050,468	720	188	14	729	187	16	1	9.90E-01	4.36E-03		
	rs7707837	chr5	71,059,828	19	239	664	25	283	623	0.812	2.10E-02	1.68E+00		
	rs13362349	chr5	71,069,348	535	334	53	482	368	82	1.27	1.40E-03	2.85E+00		
	rs6453135	chr5	71,073,992	589	286	27	542	319	54	1.31	1.10E-03	2.96E+00		
	rs16876022	chr5	71,087,987	668	233	16	637	268	22	1.2	5.20E-02	1.28E+00		
	rs6891713	chr5	71,090,060	1	52	869	2	83	846	0.615	7.20E-03	2.14E+00		
	rs16872075	chr5	71,094,436	870	50	2	850	80	2	1.56	1.40E-02	1.85E+00		
	rs2056169	chr5	71,095,468	605	283	31	548	327	57	1.34	3.20E-04	3.49E+00		
	rs9293660	chr5	71,097,489	16	233	673	22	273	637	0.819	3.10E-02	1.51E+00		
	rs4057815	chr5	71,103,912	8	157	757	4	131	797	1.28	4.00E-02	1.40E+00		
	rs13158073	chr5	71,112,634	209	461	252	249	468	215	0.847	1.20E-02	1.92E+00		
	rs7706490	chr5	71,113,290	97	421	403	113	423	394	0.935	3.50E-01	4.56E-01		
	rs17359958	chr5	71,115,774	23	222	677	23	269	640	0.836	5.30E-02	1.28E+00		
	rs6862707	chr5	71,116,907	207	462	252	244	469	219	0.859	2.30E-02	1.64E+00		
	rs1798575	chr5	71,141,418	70	375	477	76	401	455	0.919	2.60E-01	5.85E-01		
	rs2676252	chr5	71,147,098	102	417	401	102	416	411	1.02	8.30E-01	8.09E-02		
	rs2721750	chr5	71,150,434	87	388	447	80	385	467	1.06	4.30E-01	3.67E-01		
	rs10942773	chr5	71,155,842	447	388	87	467	385	80	0.943	4.30E-01	3.67E-01		
	rs1105330	chr5	71,163,553	446	390	86	467	385	80	0.943	4.30E-01	3.67E-01		
	rs2721775	chr5	71,168,107	385	416	119	372	435	123	1.05	5.20E-01	2.84E-01		
	rs4354016	chr5	71,170,863	612	282	22	648	248	34	0.945	5.50E-01	2.60E-01		
	rs12659543	chr5	71,173,487	5	146	771	6	153	773	0.952	7.10E-01	1.49E-01		
	rs4305629	chr5	71,187,581	32	300	588	36	303	592	0.978	8.20E-01	8.62E-02		
	rs10062158	chr5	71,190,550	3	92	823	1	94	837	1.04	8.60E-01	6.55E-02		
	rs1911923	chr5	71,191,363	406	430	86	433	401	98	0.972	7.10E-01	1.49E-01		
	rs12055280	chr5	71,192,651	122	458	342	137	441	354	0.988	8.80E-01	5.55E-02		
	rs16872647	chr5	71,198,943	140	459	320	155	441	333	0.991	9.20E-01	3.62E-02		
	rs4057254	chr5	71,203,197	382	439	101	404	419	109	0.974	7.30E-01	1.37E-01		
	rs6890936	chr5	71,205,261	120	436	366	123	415	394	1.05	4.70E-01	3.28E-01		
	rs4057256	chr5	71,206,370	2	84	835	4	103	825	0.792	1.30E-01	8.86E-01		
	rs10058537	chr5	71,209,059	594	296	32	587	318	27	1.03	7.70E-01	1.14E-01		
	rs4704363	chr5	71,216,205	756	160	6	774	150	8	0.95	7.00E-01	1.55E-01		
	rs6453274	chr5	71,220,674	499	361	62	501	352	79	1.06	4.90E-01	3.10E-01		
	rs12332541	chr5	71,221,485	565	313	44	576	313	43	0.98	8.30E-01	8.09E-02		
	rs6453280	chr5	71,225,029	8	169	737	8	156	761	1.1	4.30E-01	3.67E-01		
	rs10942814	chr5	71,226,779	345	447	130	359	428	145	1.01	9.40E-01	2.69E-02		
	rs4602602	chr5	71,236,426	647	243	32	644	266	22	0.999	9.80E-01	8.77E-03		
	rs10515110	chr5	71,236,666	831	88	3	843	89	0	0.933	7.10E-01	1.49E-01		
	rs2879027	chr5	71,240,017	352	454	114	377	416	137	1	9.90E-01	4.36E-03		
	rs3857394	chr5	71,243,984	8	191	723	18	195	719	0.894	2.90E-01	5.38E-01		
	rs1692383	chr5	71,255,981	70	348	504	79	363	490	0.928	3.30E-01	4.81E-01		
	rs16873241	chr5	71,256,017	818	102	1	811	115	6	1.22	1.60E-01	7.96E-01		
	rs1026507	chr5	71,258,957	24	242	656	26	252	654	0.958	6.60E-01	1.80E-01		
	rs1692418	chr5	71,261,369	45	297	580	44	306	582	0.991	9.40E-01	2.69E-02		
	rs10071437	chr5	71,265,778	121	447	354	137	427	367	0.988	8.90E-01	5.06E-02		
	rs1692420	chr5	71,266,499	10	178	734	11	186	735	0.958	7.20E-01	1.43E-01		
	rs3857397	chr5	71,269,359	130	475	317	146	453	333	0.995	9.70E-01	1.32E-02		
	rs1629473	chr5	71,272,167	29	230	663	20	240	672	1.05	6.50E-01	1.87E-01		
	rs898219	chr5	71,275,894	353	446	123	356	446	130	1.01	8.50E-01	7.06E-02		
	rs2125627	chr5	71,277,269	27	254	641	33	266	633	0.925	4.00E-01	3.98E-01		
	rs1217744	chr5	71,281,566	2	95	819	4	106	817	0.872	3.70E-01	4.32E-01		
	rs1217745	chr5	71,281,720	27	247	648	26	239	667	1.05	5.80E-01	2.37E-01		
	rs1217750	chr5	71,285,196	47	332	543	44	309	579	1.11	2.00E-01	6.99E-01		
	rs2662334	chr5	71,285,696	85	397	440	93	403	436	0.961	6.00E-01	2.22E-01		

	rs2610424	chr5	71,292,415	290	460	172	303	479	150	0.929	2.80E-01	5.53E-01		
	rs2250816	chr5	71,293,761	391	414	104	387	399	119	1.04	5.60E-01	2.52E-01		
	rs1217824	chr5	71,296,429	334	429	159	325	455	152	1.01	9.20E-01	3.62E-02		
	rs2662317	chr5	71,302,469	32	262	628	36	279	617	0.926	3.90E-01	4.09E-01		
	rs1217736	chr5	71,304,705	110	400	412	106	396	430	1.05	5.40E-01	2.68E-01		
	rs6453365	chr5	71,308,844	33	265	624	38	279	615	0.93	4.10E-01	3.87E-01		
	rs962512	chr5	71,321,316	921	0	1	930	2	0	0.989	1.00E+00	0.00E+00		
	rs1344024	chr5	71,326,135	0	3	918	1	0	930	1.52	6.90E-01	1.61E-01		
	rs13187253	chr5	71,330,913	33	263	626	37	279	616	0.93	4.10E-01	3.87E-01		
	rs7704592	chr5	71,331,087	624	266	32	615	279	38	1.08	3.90E-01	4.09E-01		
	rs1217770	chr5	71,343,136	453	393	76	473	382	77	0.962	6.20E-01	2.08E-01		
	rs1692423	chr5	71,355,018	30	246	646	21	246	665	1.09	3.70E-01	4.32E-01		
	rs1217760	chr5	71,365,042	422	402	98	424	412	96	0.999	9.90E-01	4.36E-03		
	rs1692356	chr5	71,383,640	682	221	19	704	211	17	0.926	4.50E-01	3.47E-01		
	rs1692395	chr5	71,389,241	19	220	683	17	212	703	1.07	5.10E-01	2.92E-01		
	rs1692404	chr5	71,395,533	639	252	28	651	256	25	0.976	8.10E-01	9.15E-02		
	rs1026508	chr5	71,400,037	64	376	482	74	400	458	0.903	1.70E-01	7.70E-01		
	rs1217778	chr5	71,402,652	20	205	696	16	219	697	0.986	9.20E-01	3.62E-02		
	rs1217785	chr5	71,404,593	242	465	215	248	442	242	1.05	5.10E-01	2.92E-01		
	rs16874468	chr5	71,405,201	13	194	715	19	208	705	0.891	2.70E-01	5.69E-01		
	rs1217791	chr5	71,406,940	63	375	483	74	401	456	0.894	1.30E-01	8.86E-01		
	rs1217793	chr5	71,408,637	16	185	721	13	197	722	0.981	8.90E-01	5.06E-02		
	rs1217795	chr5	71,411,270	16	186	720	13	197	722	0.987	9.30E-01	3.15E-02		
	rs17300302	chr5	71,411,721	42	310	570	41	295	596	1.07	4.10E-01	3.87E-01		
	rs1217800	chr5	71,412,888	147	444	331	156	423	352	1.02	7.60E-01	1.19E-01		
	rs17300532	chr5	71,416,111	61	359	502	55	359	518	1.05	5.40E-01	2.68E-01		
	rs6897294	chr5	71,416,670	488	361	64	507	354	63	0.959	6.10E-01	2.15E-01		
	rs1217806	chr5	71,417,118	350	444	128	369	436	127	0.961	5.80E-01	2.37E-01		
	rs1217816	chr5	71,424,094	299	453	170	320	427	185	0.99	9.10E-01	4.10E-02		
	rs1217823	chr5	71,429,871	121	418	383	133	428	370	0.938	3.70E-01	4.32E-01		
	rs3112400	chr5	71,430,625	611	276	35	603	297	32	1.04	6.70E-01	1.74E-01		
	rs6880685	chr5	71,433,805	25	250	647	35	240	657	0.974	8.00E-01	9.69E-02		
	rs2337390	chr5	71,444,623	79	359	484	65	386	481	1.02	8.40E-01	7.57E-02		
	rs7732739	chr5	71,445,139	579	298	44	578	299	54	1.06	5.30E-01	2.76E-01		
	rs3101186	chr5	71,445,325	25	265	631	19	276	637	1.02	8.70E-01	6.05E-02		
	rs1531312	chr5	71,447,474	82	368	471	75	393	463	0.986	8.80E-01	5.55E-02		
	rs1466344	chr5	71,449,384	12	177	722	14	176	738	1	9.90E-01	4.36E-03		
	rs1459215	chr5	71,450,356	230	460	229	230	450	251	1.05	4.90E-01	3.10E-01		
	rs3101187	chr5	71,452,383	708	198	16	715	198	19	1.02	9.00E-01	4.58E-02		
	rs10474585	chr5	71,457,764	146	450	325	151	457	323	0.98	7.90E-01	1.02E-01		
	rs4704553	chr5	71,466,651	147	476	299	159	448	325	1.03	7.10E-01	1.49E-01		
	rs4704554	chr5	71,467,472	147	476	299	159	447	326	1.03	6.80E-01	1.67E-01		
	rs4703774	chr5	71,473,018	78	406	437	108	378	443	0.934	3.60E-01	4.44E-01		
	rs4569852	chr5	71,477,345	425	401	96	438	395	99	0.984	8.50E-01	7.06E-02		
	rs12655013	chr5	71,486,711	730	177	15	758	155	19	0.913	4.20E-01	3.77E-01		
	rs6872830	chr5	71,489,931	14	232	676	24	245	663	0.88	1.80E-01	7.45E-01		
	rs2337692	chr5	71,491,374	57	351	514	61	357	514	0.975	7.70E-01	1.14E-01		
	rs10063919	chr5	71,491,849	2	96	824	3	121	808	0.784	9.00E-02	1.05E+00		
	rs1545170	chr5	71,492,764	702	199	8	665	226	17	1.25	2.90E-02	1.54E+00		
	rs17301591	chr5	71,497,821	41	310	571	30	315	587	1.07	4.10E-01	3.87E-01		
	rs3098378	chr5	71,503,426	329	450	143	367	434	129	0.892	9.70E-02	1.01E+00		
	rs2118695	chr5	71,507,085	111	394	417	135	416	381	0.861	3.30E-02	1.48E+00		
	rs12186804	chr5	71,507,469	587	305	29	625	283	22	0.869	1.10E-01	9.59E-01		
	rs10055619	chr5	71,510,030	9	195	718	13	190	729	0.996	9.90E-01	4.36E-03		
	rs2196760	chr5	71,510,043	118	424	380	120	423	389	1.01	9.10E-01	4.10E-02		
	rs2337694	chr5	71,512,661	23	248	650	27	261	644	0.934	4.70E-01	3.28E-01		
	rs2028260	chr5	71,512,713	391	441	89	387	445	100	1.05	5.40E-01	2.68E-01		
	rs6874186	chr5	71,512,871	402	415	105	416	409	107	0.979	7.90E-01	1.02E-01		
	rs3792806	chr5	71,517,643	601	287	34	604	299	29	0.994	9.70E-01	1.32E-02		
	rs16875959	chr5	71,519,497	11	205	705	10	169	752	1.24	4.10E-02	1.39E+00		
	rs1561398	chr5	71,522,541	49	353	519	48	337	547	1.07	3.90E-01	4.09E-01		
	rs7736611	chr5	71,524,660	125	437	360	126	435	371	1.02	8.30E-01	8.09E-02		
	rs1866374	chr5	71,526,718	30	244	648	23	273	636	0.956	6.40E-01	1.94E-01		
	rs13153166	chr5	71,530,687	822	95	5	844	86	2	0.84	2.70E-01	5.69E-01		
	rs3805452	chr5	71,530,797	529	333	60	513	364	55	1.05	5.70E-01	2.44E-01		
	rs3828616	chr5	71,532,175	418	410	94	413	426	93	1.02	8.20E-01	8.62E-02		
	rs930391	chr5	71,539,443	700	201	12	687	226	11	1.1	3.50E-01	4.56E-01		
	rs10039706	chr5	71,542,190	157	458	307	181	440	311	0.954	5.00E-01	3.01E-01		
	rs17302844	chr5	71,546,745	108	416	398	111	433	388	0.962	6.00E-01	2.22E-01		
	rs2289281	chr5	71,557,438	718	194	10	743	182	7	0.895	3.10E-01	5.09E-01		

	rs3213894	chr5	71,563,866	405	403	114	390	428	114	1.04	5.60E-01	2.52E-01		
	rs10073728	chr5	71,567,938	698	208	16	715	200	16	0.951	6.50E-01	1.87E-01		
	rs12697910	chr5	71,570,309	2	87	833	2	89	841	0.989	1.00E+00	0.00E+00		
	rs11956209	chr5	71,570,610	749	166	7	727	196	9	1.2	1.00E-01	1.00E+00		
	rs4434362	chr5	71,575,797	918	3	1	931	1	0	0.197	1.20E-01	9.21E-01		
	rs2289279	chr5	71,577,959	681	223	18	687	227	18	1.01	9.90E-01	4.36E-03		
	rs10454918	chr5	71,578,770	41	357	524	59	358	513	0.908	2.20E-01	6.58E-01		
	rs7710169	chr5	71,584,804	1	53	867	2	52	878	0.994	9.50E-01	2.23E-02		
	rs4703813	chr5	71,587,943	11	183	728	9	190	733	0.996	9.90E-01	4.36E-03		
	rs11749445	chr5	71,603,661	469	379	59	523	338	56	0.862	5.30E-02	1.28E+00		
	rs2047588	chr5	71,614,658	380	437	103	373	431	126	1.08	2.80E-01	5.53E-01		
	rs9293273	chr5	71,657,184	29	286	604	33	263	635	1.07	4.30E-01	3.67E-01		
	rs10942271	chr5	71,670,468	725	182	11	732	186	9	0.989	9.60E-01	1.77E-02		
	rs7734699	chr5	71,690,514	871	51	0	879	51	0	0.991	9.60E-01	1.77E-02		
	rs10223066	chr5	71,697,344	475	387	60	521	352	59	0.889	1.20E-01	9.21E-01		
	rs4507472	chr5	71,702,830	112	407	403	110	427	395	0.978	7.80E-01	1.08E-01		
	rs9293282	chr5	71,703,434	12	194	716	13	180	739	1.08	4.90E-01	3.10E-01		
	rs9293289	chr5	71,719,641	72	380	469	89	387	456	0.914	2.30E-01	6.38E-01		
	rs13158670	chr5	71,723,979	186	497	239	186	448	298	1.13	6.00E-02	1.22E+00		
	rs11750874	chr5	71,726,098	10	169	743	10	188	734	0.909	4.00E-01	3.98E-01		
	rs4703854	chr5	71,729,219	548	307	50	539	325	48	1.03	7.00E-01	1.55E-01		
	rs4703855	chr5	71,729,655	79	395	446	97	407	428	0.903	1.60E-01	7.96E-01		
	rs10040192	chr5	71,731,636	239	442	241	216	468	248	1.07	3.40E-01	4.69E-01		
	rs7445013	chr5	71,733,733	262	459	201	240	480	212	1.08	2.90E-01	5.38E-01		
	rs2338216	chr5	71,737,570	198	460	264	211	480	241	0.924	2.40E-01	6.20E-01		
	rs16878312	chr5	71,741,140	72	374	473	82	386	464	0.938	3.90E-01	4.09E-01		
	rs6452453	chr5	71,748,955	557	313	52	549	333	50	1.04	6.80E-01	1.67E-01		
	rs7723734	chr5	71,753,361	394	413	115	387	430	115	1.02	7.60E-01	1.19E-01		
	rs13180645	chr5	71,755,940	6	135	781	4	133	795	1.06	6.90E-01	1.61E-01		
	rs4703872	chr5	71,759,400	197	460	264	212	478	242	0.922	2.30E-01	6.38E-01		
	rs6452473	chr5	71,776,850	14	186	720	17	201	714	0.912	3.90E-01	4.09E-01		
	rs17378840	chr5	71,777,912	55	369	498	59	340	533	1.08	3.40E-01	4.69E-01		
	rs13177417	chr5	71,779,710	136	441	345	137	450	345	0.993	9.40E-01	2.69E-02		
	rs10068109	chr5	71,779,955	14	181	726	16	198	718	0.909	3.80E-01	4.20E-01		
	rs10070001	chr5	71,780,420	61	412	449	84	398	449	0.933	3.60E-01	4.44E-01		
	rs16878635	chr5	71,783,204	31	299	592	37	294	601	0.99	9.30E-01	3.15E-02		
	rs3112482	chr5	71,786,656	3	82	837	10	103	819	0.709	2.00E-02	1.70E+00		
	rs6862221	chr5	71,790,718	602	290	29	600	296	35	1.05	5.80E-01	2.37E-01		
	rs7721922	chr5	71,791,858	96	423	403	106	425	401	0.964	6.20E-01	2.08E-01		
	rs2278600	chr5	71,792,426	22	222	678	21	232	679	0.978	8.50E-01	7.06E-02		
	rs12054660	chr5	71,796,031	761	156	2	785	137	10	0.965	8.00E-01	9.69E-02		
	rs1019457	chr5	71,798,910	48	361	513	53	347	532	1.03	7.60E-01	1.19E-01		
	rs6874676	chr5	71,799,471	509	339	74	503	372	57	0.983	8.40E-01	7.57E-02		
	rs1421968	chr5	71,802,075	801	118	3	833	95	4	0.811	1.50E-01	8.24E-01		
	rs4703910	chr5	71,807,866	580	313	29	585	308	39	1.04	6.90E-01	1.61E-01		
	rs10043659	chr5	71,817,595	580	310	32	579	311	42	1.06	5.20E-01	2.84E-01		
	rs246565	chr5	71,845,003	25	282	615	32	286	614	0.95	5.70E-01	2.44E-01		
	rs246580	chr5	71,851,370	25	278	619	32	284	616	0.943	5.10E-01	2.92E-01		
	rs165986	chr5	71,857,770	28	287	607	34	298	600	0.935	4.50E-01	3.47E-01		
	rs381734	chr5	71,879,511	35	326	559	46	328	554	0.938	4.40E-01	3.57E-01		
	rs16899650	chr5	71,883,978	0	2	920	0	1	931	2.02	6.20E-01	2.08E-01		
	rs1379263	chr5	71,885,325	9	149	764	8	170	754	0.898	3.70E-01	4.32E-01		
	rs16899839	chr5	71,907,547	919	3	0	931	1	0	0.329	3.70E-01	4.32E-01		
	rs42775	chr5	71,916,270	81	409	432	77	412	443	1.03	7.20E-01	1.43E-01		
	rs33424	chr5	71,918,365	434	405	81	441	411	76	0.978	7.80E-01	1.08E-01		
	rs33425	chr5	71,928,668	327	454	139	335	451	146	1	9.90E-01	4.36E-03		
	rs636926	chr5	72,455,373	61	359	502	54	363	514	1.04	6.10E-01	2.15E-01		
	rs570888	chr5	72,457,774	61	358	503	54	363	515	1.04	6.20E-01	2.08E-01		
	rs703871	chr5	72,461,417	522	341	58	530	332	70	1.03	7.50E-01	1.25E-01		
	rs1083428	chr5	72,462,531	549	315	52	562	306	62	1.01	8.90E-01	5.06E-02		
	rs488174	chr5	72,465,102	1	55	866	2	50	880	1.07	8.00E-01	9.69E-02		
	rs549579	chr5	72,467,213	225	428	269	201	436	295	1.11	1.10E-01	9.59E-01		
	rs13160226	chr5	72,469,980	539	315	68	538	334	60	0.995	9.70E-01	1.32E-02		
	rs471507	chr5	72,470,057	156	389	377	183	403	346	0.873	4.70E-02	1.33E+00		
	rs484573	chr5	72,476,863	9	154	759	16	165	749	0.868	2.20E-01	6.58E-01		
	rs648441	chr5	72,482,521	157	389	376	184	409	339	0.862	2.90E-02	1.54E+00		
	rs71542	chr5	72,487,477	669	231	22	686	223	23	0.962	7.10E-01	1.49E-01		
	rs7720612	chr5	72,490,276	788	127	7	799	130	3	0.951	7.30E-01	1.37E-01		
	rs9293528	chr5	72,492,023	694	213	15	727	190	15	0.882	2.20E-01	6.58E-01		
	rs17731153	chr5	72,492,177	66	312	537	58	312	557	1.07	4.20E-01	3.77E-01		

	rs1457139	chr5	72,492,876	344	425	141	357	423	144	0.984	8.40E-01	7.57E-02		
	rs575415	chr5	72,495,882	733	181	8	735	186	11	1.05	6.80E-01	1.67E-01		
	rs576243	chr5	72,495,951	16	243	663	30	227	675	0.963	7.20E-01	1.43E-01		
	rs6879012	chr5	72,498,637	99	346	477	101	376	455	0.931	3.40E-01	4.69E-01		
	rs3733859	chr5	72,508,520	435	392	95	450	400	82	0.941	4.10E-01	3.87E-01		
	rs6893265	chr5	72,509,655	681	220	21	677	226	29	1.09	4.00E-01	3.98E-01		
	rs7702108	chr5	72,529,022	263	465	194	279	443	210	1	9.90E-01	4.36E-03		
	rs4416586	chr5	72,530,549	186	459	277	194	447	291	1.01	9.00E-01	4.58E-02		
	rs4703597	chr5	72,531,935	7	144	771	2	158	772	0.985	9.40E-01	2.69E-02		
	rs3935430	chr5	72,536,852	289	460	173	273	456	203	1.11	1.30E-01	8.86E-01		
	rs4457052	chr5	72,564,678	420	406	96	426	395	111	1.03	6.90E-01	1.61E-01		
	rs4540159	chr5	72,582,234	486	348	86	500	349	83	0.969	7.00E-01	1.55E-01		
	rs7702331	chr5	72,586,890	349	422	151	374	405	153	0.954	5.10E-01	2.92E-01		
	rs13155213	chr5	72,587,093	490	346	86	498	350	84	0.985	8.70E-01	6.05E-02		
	rs4703599	chr5	72,597,052	42	251	629	19	277	636	1.09	3.30E-01	4.81E-01		
	rs4704066	chr5	72,601,244	184	475	263	201	435	295	1.03	6.60E-01	1.80E-01		
	rs7727448	chr5	72,605,701	312	443	164	311	437	182	1.05	5.10E-01	2.92E-01		
	rs13165516	chr5	72,612,771	488	371	63	500	367	65	0.985	8.70E-01	6.05E-02		
	rs4235676	chr5	72,614,799	57	345	520	51	340	541	1.07	4.20E-01	3.77E-01		
	rs4422515	chr5	72,623,211	325	455	142	343	415	174	1.04	6.20E-01	2.08E-01		
	rs4703603	chr5	72,628,339	474	373	74	469	372	91	1.07	3.50E-01	4.56E-01		
	rs4704077	chr5	72,632,403	148	408	366	138	432	362	1.01	9.30E-01	3.15E-02		
	rs9293560	chr5	72,637,254	242	466	214	265	447	220	0.965	6.10E-01	2.15E-01		
	rs9293561	chr5	72,639,843	55	308	559	50	309	573	1.04	6.20E-01	2.08E-01		
	rs4389670	chr5	72,646,286	407	414	101	420	409	103	0.982	8.20E-01	8.62E-02		
	rs4582254	chr5	72,646,950	263	465	194	294	441	197	0.943	3.90E-01	4.09E-01		
	rs3922417	chr5	72,655,258	739	174	9	748	171	13	1.02	9.20E-01	3.62E-02		
	rs2972195	chr5	72,656,100	579	313	23	604	280	41	0.997	1.00E+00	0.00E+00		
	rs6895622	chr5	72,660,292	11	228	683	20	210	702	1.01	9.30E-01	3.15E-02		
	rs4704082	chr5	72,685,941	394	423	104	381	432	119	1.08	3.00E-01	5.23E-01		
	rs4443403	chr5	72,690,060	722	191	9	741	182	9	0.94	5.90E-01	2.29E-01		
	rs4515275	chr5	72,690,217	842	78	2	856	74	2	0.938	7.50E-01	1.25E-01		
	rs6452972	chr5	72,695,929	2	121	799	3	115	814	1.05	7.80E-01	1.08E-01		
	rs2972212	chr5	72,696,541	289	459	174	273	476	182	1.06	4.30E-01	3.67E-01		
	rs6875658	chr5	72,706,878	766	147	9	777	147	8	0.975	8.70E-01	6.05E-02		
	rs4639198	chr5	72,710,994	793	125	4	815	109	8	0.925	5.90E-01	2.29E-01		
	rs4418079	chr5	72,726,876	19	223	679	18	210	703	1.08	4.20E-01	3.77E-01		
	rs6863278	chr5	72,727,144	3	124	795	4	115	813	1.07	6.30E-01	2.01E-01		
	rs958583	chr5	72,758,558	97	381	442	93	423	409	0.926	2.90E-01	5.38E-01		
	rs874973	chr5	72,773,651	788	129	5	811	112	9	0.92	5.50E-01	2.60E-01		
	rs1449272	chr5	72,785,919	787	130	5	811	112	9	0.913	5.10E-01	2.92E-01		
	rs968660	chr5	72,803,922	6	94	822	5	107	820	0.911	5.40E-01	2.68E-01		
	rs347251	chr5	72,807,818	88	408	426	92	408	431	0.994	9.60E-01	1.77E-02		
	rs12659487	chr5	72,820,607	7	95	820	5	110	817	0.913	5.50E-01	2.60E-01		
	rs347243	chr5	72,826,449	504	356	62	495	369	67	1.05	5.20E-01	2.84E-01		
	rs17663872	chr5	72,827,065	748	163	11	758	165	9	0.976	8.70E-01	6.05E-02		
	rs347245	chr5	72,828,807	124	443	354	138	432	362	0.98	7.90E-01	1.02E-01		
	rs2298890	chr5	72,837,683	7	94	821	5	109	818	0.912	5.50E-01	2.60E-01		
	rs7703551	chr5	72,842,274	821	95	6	819	108	5	1.1	5.50E-01	2.60E-01		
	rs10064416	chr5	72,844,806	2	114	806	4	112	816	0.994	9.80E-01	8.77E-03		
	rs2914544	chr5	72,845,587	210	471	241	199	470	263	1.07	3.00E-01	5.23E-01		
	rs10462345	chr5	72,846,913	678	225	19	664	248	20	1.1	3.30E-01	4.81E-01		
	rs6453008	chr5	72,851,543	291	439	192	254	467	211	1.13	6.60E-02	1.18E+00		
	rs3857374	chr5	72,854,468	277	439	204	247	464	220	1.11	1.30E-01	8.86E-01		
	rs819561	chr5	72,854,962	29	258	635	29	280	623	0.934	4.50E-01	3.47E-01		
	rs819557	chr5	72,857,853	370	414	136	325	468	136	1.11	1.20E-01	9.21E-01		
	rs865340	chr5	72,858,222	13	213	696	17	223	692	0.931	4.90E-01	3.10E-01		
	rs12651911	chr5	72,858,386	524	350	47	506	367	57	1.1	2.30E-01	6.38E-01		
	rs819550	chr5	72,860,712	355	438	129	361	433	138	1.01	8.80E-01	5.55E-02		
	rs819571	chr5	72,863,969	17	206	699	19	232	680	0.882	2.10E-01	6.78E-01		
	rs17663979	chr5	72,865,786	4	76	842	3	86	843	0.919	6.40E-01	1.94E-01		
	rs819569	chr5	72,867,949	339	437	146	301	469	162	1.13	6.70E-02	1.17E+00		
	rs819600	chr5	72,873,338	148	454	320	126	464	342	1.1	1.70E-01	7.70E-01		
	rs819591	chr5	72,879,536	572	311	34	542	342	46	1.17	5.50E-02	1.26E+00		
	rs703879	chr5	72,890,735	90	424	408	90	393	449	1.1	2.00E-01	6.99E-01		
	rs347236	chr5	72,898,045	408	419	90	448	388	90	0.912	2.10E-01	6.78E-01		
	rs1220634	chr5	72,904,302	516	357	49	486	378	68	1.16	4.90E-02	1.31E+00		
	rs343120	chr5	72,911,791	59	306	557	64	346	522	0.876	9.10E-02	1.04E+00		
	rs13170849	chr5	72,917,884	169	445	308	162	449	321	1.04	5.60E-01	2.52E-01		
	rs2249074	chr5	72,926,029	465	389	68	504	353	75	0.929	3.30E-01	4.81E-01		

	rs980631	chr5	72,927,514	257	453	199	285	442	198	0.941	3.80E-01	4.20E-01		
	rs16870631	chr5	72,929,457	2	68	846	1	70	850	1.01	9.60E-01	1.77E-02		
	rs17633441	chr5	72,964,003	273	467	182	302	447	183	0.943	3.90E-01	4.09E-01		
	rs6890730	chr5	72,968,811	848	73	1	853	79	0	1.04	8.60E-01	6.55E-02		
	rs2464432	chr5	72,970,310	67	378	475	73	394	465	0.945	4.60E-01	3.37E-01		
	rs13178946	chr5	72,995,212	207	474	239	207	456	268	1.06	3.70E-01	4.32E-01		
	rs10805881	chr5	72,999,704	470	376	76	473	382	77	1.01	9.60E-01	1.77E-02		
	rs4704090	chr5	73,002,834	470	376	76	473	382	77	1.01	9.60E-01	1.77E-02		
	rs754374	chr5	73,014,534	549	323	50	527	355	50	1.08	3.10E-01	5.09E-01		
	rs750345	chr5	73,015,009	275	467	180	304	456	171	0.922	2.30E-01	6.38E-01		
	rs17552288	chr5	73,021,756	5	133	784	7	132	793	0.989	9.80E-01	8.77E-03		
	rs6868499	chr5	73,025,228	859	62	1	858	74	0	1.15	4.70E-01	3.28E-01		
	rs16870743	chr5	73,052,566	501	355	66	473	388	71	1.11	1.80E-01	7.45E-01		
	rs7703182	chr5	73,058,823	504	355	63	479	386	67	1.1	2.30E-01	6.38E-01		
	rs17664695	chr5	73,066,698	852	66	3	851	76	1	1.08	7.10E-01	1.49E-01		
	rs11746513	chr5	73,072,829	454	376	91	421	420	89	1.09	2.40E-01	6.20E-01		
	rs952995	chr5	73,077,540	53	310	559	43	354	535	0.943	4.70E-01	3.28E-01		
	rs905159	chr5	73,082,471	78	364	480	81	424	427	0.857	3.40E-02	1.47E+00		
	rs10515162	chr5	73,090,012	776	139	7	783	144	5	0.995	9.80E-01	8.77E-03		
	rs10515163	chr5	73,091,337	478	364	79	427	422	82	1.16	4.10E-02	1.39E+00		
	rs6875883	chr5	73,095,704	921	1	0	932	0	0	0	5.00E-01	3.01E-01		
	rs16870823	chr5	73,096,410	865	57	0	865	67	0	1.17	4.50E-01	3.47E-01		
	rs10062885	chr5	73,104,644	545	315	62	507	359	65	1.14	9.20E-02	1.04E+00		
	rs7712515	chr5	73,104,765	495	353	74	467	401	64	1.06	4.30E-01	3.67E-01		
	rs10065074	chr5	73,105,788	797	121	4	777	148	7	1.27	6.30E-02	1.20E+00		
	rs7714670	chr5	73,108,110	270	449	203	243	499	190	1.03	6.50E-01	1.87E-01		
	rs11960055	chr5	73,108,956	2	95	825	3	120	809	0.783	8.80E-02	1.06E+00		
	rs6453022	chr5	73,112,267	231	457	234	222	506	204	0.956	5.10E-01	2.92E-01		
	rs4597943	chr5	73,123,824	219	456	247	212	485	235	0.989	8.90E-01	5.06E-02		
	rs12653477	chr5	73,125,845	248	451	223	245	477	210	0.979	7.80E-01	1.08E-01		
	rs2973548	chr5	73,126,984	223	451	248	210	477	245	1.02	7.80E-01	1.08E-01		
	rs6883409	chr5	73,128,025	850	69	3	842	87	3	1.24	2.00E-01	6.99E-01		
	rs716707	chr5	73,133,625	4	61	857	2	59	871	1.11	6.10E-01	2.15E-01		
	rs2931417	chr5	73,140,466	279	457	186	301	455	176	0.935	3.20E-01	4.95E-01		
	rs1009200	chr5	73,141,031	160	440	321	154	444	334	1.04	5.90E-01	2.29E-01		
	rs2973540	chr5	73,144,309	347	415	160	346	443	143	0.969	6.60E-01	1.80E-01		
	rs4703613	chr5	73,144,329	44	310	568	48	317	567	0.967	7.00E-01	1.55E-01		
	rs6868665	chr5	73,148,104	123	401	398	109	428	395	1.02	8.10E-01	9.15E-02		
	rs16870904	chr5	73,149,406	582	293	46	601	292	38	0.932	4.10E-01	3.87E-01		
	rs1038559	chr5	73,149,488	88	353	481	86	374	472	0.971	7.10E-01	1.49E-01		
	rs7705309	chr5	73,150,757	155	405	348	138	439	336	1.01	9.20E-01	3.62E-02		
	rs4704098	chr5	73,151,031	13	192	717	12	217	703	0.903	3.30E-01	4.81E-01		
	rs11949468	chr5	73,151,683	15	202	703	16	203	713	1	9.60E-01	1.77E-02		
	rs2931431	chr5	73,160,902	485	358	79	475	385	71	1.02	8.60E-01	6.55E-02		
	rs10515164	chr5	73,164,783	781	131	9	803	124	5	0.88	3.30E-01	4.81E-01		
	rs2973575	chr5	73,170,750	351	416	155	345	448	139	0.982	8.20E-01	8.62E-02		
	rs10040601	chr5	73,171,241	309	424	189	308	453	171	0.966	6.30E-01	2.01E-01		
	rs16870944	chr5	73,178,862	802	110	6	813	113	4	0.978	9.20E-01	3.62E-02		
	rs2931443	chr5	73,180,057	212	456	253	214	469	247	0.982	8.10E-01	9.15E-02		
	rs2973567	chr5	73,181,782	212	459	251	216	469	246	0.98	7.80E-01	1.08E-01		
	rs2973566	chr5	73,184,237	93	372	437	84	367	444	1.05	5.20E-01	2.84E-01		
	rs17553316	chr5	73,184,872	798	118	6	809	119	4	0.964	8.30E-01	8.09E-02		
	rs17732825	chr5	73,184,921	6	115	801	4	115	813	1.05	7.80E-01	1.08E-01		
	rs16870977	chr5	73,185,069	21	210	682	15	220	692	1.03	8.20E-01	8.62E-02		
	rs2973564	chr5	73,186,789	438	376	93	455	381	87	0.958	5.70E-01	2.44E-01		
	rs2973563	chr5	73,189,510	198	435	288	183	479	270	0.991	9.20E-01	3.62E-02		
	rs2931434	chr5	73,194,854	113	410	399	98	413	421	1.08	2.60E-01	5.85E-01		
	rs6869486	chr5	73,195,357	598	285	39	577	312	43	1.11	2.20E-01	6.58E-01		
	rs7720220	chr5	73,197,110	762	150	10	740	183	9	1.19	1.30E-01	8.86E-01		
	rs2973558	chr5	73,199,721	113	410	399	98	414	420	1.08	2.70E-01	5.69E-01		
	rs2931418	chr5	73,200,644	399	407	116	420	414	98	0.917	2.30E-01	6.38E-01		
	rs2973556	chr5	73,201,136	292	456	174	293	470	169	0.99	9.00E-01	4.58E-02		
	rs1352200	chr5	73,203,090	25	228	669	16	228	688	1.1	3.50E-01	4.56E-01		
	rs2973550	chr5	73,204,335	317	441	159	294	482	151	1.04	6.00E-01	2.22E-01		
	rs3749645	chr5	73,215,428	16	169	733	12	215	702	0.833	8.10E-02	1.09E+00		
	rs7732677	chr5	73,217,006	810	105	2	832	96	3	0.917	5.90E-01	2.29E-01		
	rs729272	chr5	73,221,260	604	276	42	578	315	39	1.1	2.50E-01	6.02E-01		
	rs2973529	chr5	73,224,901	140	439	343	116	445	371	1.12	1.00E-01	1.00E+00		
	rs3749642	chr5	73,232,396	148	471	303	164	459	309	0.975	7.20E-01	1.43E-01		
	rs3749640	chr5	73,232,566	101	404	417	87	403	442	1.09	2.30E-01	6.38E-01		

	rs283622	chr5	73,234,120	201	467	245	216	459	255	0.988	8.80E-01	5.55E-02		
	rs283623	chr5	73,234,405	287	464	169	297	463	172	0.988	8.80E-01	5.55E-02		
	rs283624	chr5	73,234,657	237	476	208	251	472	208	0.971	6.80E-01	1.67E-01		
	rs283626	chr5	73,237,008	199	458	265	196	475	261	0.996	9.80E-01	8.77E-03		
	rs2434215	chr5	73,237,747	27	257	638	34	307	591	0.806	1.20E-02	1.92E+00		
	rs2931425	chr5	73,238,236	216	454	252	202	478	252	1.03	6.80E-01	1.67E-01		
	rs283619	chr5	73,239,166	389	405	128	375	440	117	1.01	8.70E-01	6.05E-02		
	rs10515168	chr5	73,242,423	5	163	754	4	154	774	1.09	5.00E-01	3.01E-01		
	rs2931426	chr5	73,245,007	260	454	208	295	453	183	0.879	5.50E-02	1.26E+00		
	rs392344	chr5	73,245,048	453	365	104	442	396	94	1.01	8.90E-01	5.06E-02		
	rs411585	chr5	73,247,072	50	337	532	49	364	517	0.944	4.70E-01	3.28E-01		
	rs434008	chr5	73,250,537	16	191	715	7	188	737	1.13	2.50E-01	6.02E-01		
	rs425911	chr5	73,250,793	16	189	715	7	186	736	1.13	2.50E-01	6.02E-01		
	rs16871035	chr5	73,254,617	1	71	848	1	80	851	0.898	5.70E-01	2.44E-01		
	rs2973534	chr5	73,260,445	782	131	9	760	167	5	1.19	1.40E-01	8.54E-01		
	rs283628	chr5	73,261,646	124	432	366	114	457	361	1.01	9.60E-01	1.77E-02		
	rs1684850	chr5	73,265,862	33	317	572	31	283	618	1.15	9.10E-02	1.04E+00		
	rs1650439	chr5	73,266,817	352	432	130	345	449	131	1.02	7.40E-01	1.31E-01		
	rs283592	chr5	73,276,726	297	471	154	294	481	157	1.02	8.30E-01	8.09E-02		
	rs283596	chr5	73,278,467	296	471	154	293	482	157	1.02	8.30E-01	8.09E-02		
	rs283608	chr5	73,283,089	304	466	152	296	485	151	1.02	8.00E-01	9.69E-02		
	rs283612	chr5	73,286,248	349	438	135	334	465	133	1.04	6.30E-01	2.01E-01		
	rs2450438	chr5	73,290,296	629	263	30	606	291	35	1.13	1.60E-01	7.96E-01		
	rs17733544	chr5	73,293,966	661	237	24	649	256	27	1.09	3.50E-01	4.56E-01		
	rs388272	chr5	73,294,996	174	464	282	182	491	259	0.932	3.00E-01	5.23E-01		
	rs17733586	chr5	73,295,365	1	74	847	1	84	847	0.889	5.10E-01	2.92E-01		
	rs416286	chr5	73,297,869	487	362	73	474	396	62	1.02	8.40E-01	7.57E-02		
	rs453036	chr5	73,299,713	143	437	342	143	464	325	0.958	5.40E-01	2.68E-01		
	rs511623	chr5	73,300,426	143	436	343	143	464	325	0.956	5.20E-01	2.84E-01		
	rs410914	chr5	73,301,141	142	435	345	142	463	327	0.956	5.20E-01	2.84E-01		
	rs671716	chr5	73,302,245	104	408	410	92	452	388	0.969	6.70E-01	1.74E-01		
	rs282372	chr5	73,303,018	170	468	283	184	478	270	0.94	3.70E-01	4.32E-01		
	rs9986187	chr5	73,303,123	13	165	744	13	177	735	0.937	5.80E-01	2.37E-01		
	rs378872	chr5	73,303,585	286	463	170	270	479	183	1.07	3.30E-01	4.81E-01		
	rs414915	chr5	73,304,421	68	328	526	67	361	504	0.93	3.50E-01	4.56E-01		
	rs10038733	chr5	73,307,698	13	165	744	14	178	740	0.93	5.30E-01	2.76E-01		
	rs282376	chr5	73,308,860	95	393	429	90	429	409	0.954	5.30E-01	2.76E-01		
	rs403936	chr5	73,309,151	51	340	531	60	364	508	0.899	1.70E-01	7.70E-01		
	rs282377	chr5	73,309,406	5	75	842	2	65	865	1.26	1.90E-01	7.21E-01		
	rs384834	chr5	73,309,452	161	469	292	178	478	276	0.928	2.70E-01	5.69E-01		
	rs6894385	chr5	73,312,659	617	276	29	634	273	25	0.948	5.60E-01	2.52E-01		
	rs282381	chr5	73,313,129	228	480	212	263	468	200	0.904	1.30E-01	8.86E-01		
	rs2973536	chr5	73,316,114	223	483	215	214	480	238	1.07	3.10E-01	5.09E-01		
	rs255597	chr5	73,318,007	50	338	534	59	364	509	0.893	1.50E-01	8.24E-01		
	rs1904170	chr5	73,319,448	163	466	288	180	475	274	0.931	3.00E-01	5.23E-01		
	rs9293613	chr5	73,322,054	920	2	0	932	0	0	0	2.50E-01	6.02E-01		
	rs10058557	chr5	73,322,836	150	471	301	161	480	291	0.951	4.70E-01	3.28E-01		
	rs255592	chr5	73,323,167	473	378	71	481	377	74	0.998	9.90E-01	4.36E-03		
	rs17666430	chr5	73,331,083	783	131	8	777	145	10	1.12	3.60E-01	4.44E-01		
	rs1002830	chr5	73,332,988	588	279	27	606	270	25	0.943	5.30E-01	2.76E-01		
	rs194274	chr5	73,344,099	17	214	691	16	245	671	0.89	2.40E-01	6.20E-01		
	rs187637	chr5	73,345,060	301	460	161	282	487	163	1.05	4.80E-01	3.19E-01		
	rs10078294	chr5	73,349,730	293	446	180	276	480	174	1.03	7.10E-01	1.49E-01		
	rs3101727	chr5	73,364,958	303	443	176	320	445	166	0.945	4.10E-01	3.87E-01		
	rs410390	chr5	73,365,915	102	410	409	106	383	443	1.07	3.80E-01	4.20E-01		
	rs17636051	chr5	73,367,510	0	62	860	1	68	863	0.892	5.80E-01	2.37E-01		
	rs285600	chr5	73,367,540	888	34	0	891	41	0	1.2	5.10E-01	2.92E-01		
	rs285599	chr5	73,374,870	112	405	405	97	386	449	1.15	5.60E-02	1.25E+00		
	rs285598	chr5	73,375,361	112	405	405	97	386	449	1.15	5.60E-02	1.25E+00		
	rs255585	chr5	73,379,702	380	416	126	415	407	110	0.892	1.10E-01	9.59E-01		
	rs16871304	chr5	73,387,781	25	244	653	19	247	666	1.05	6.10E-01	2.15E-01		
	rs6861281	chr5	73,389,024	803	114	3	820	109	3	0.942	7.10E-01	1.49E-01		
	rs12522885	chr5	73,391,892	24	245	653	19	246	666	1.05	6.20E-01	2.08E-01		
	rs286645	chr5	73,402,451	413	411	98	444	386	101	0.941	4.00E-01	3.98E-01		
	rs286641	chr5	73,403,994	532	333	56	489	371	72	1.2	1.80E-02	1.74E+00		
	rs286636	chr5	73,406,589	541	324	51	498	361	69	1.21	1.20E-02	1.92E+00		
	rs152044	chr5	73,407,434	346	457	119	389	416	127	0.928	2.90E-01	5.38E-01		
	rs173483	chr5	73,408,092	346	456	120	386	420	126	0.93	3.00E-01	5.23E-01		
	rs1818123	chr5	73,411,975	159	474	289	163	455	314	1.04	5.40E-01	2.68E-01		
	rs768262	chr5	73,415,087	403	421	93	435	381	114	0.984	8.50E-01	7.06E-02		

	rs1995385	chr5	73,415,681	559	324	38	564	312	56	1.06	4.70E-01	3.28E-01		
	rs718164	chr5	73,417,137	559	325	38	565	311	56	1.06	5.10E-01	2.92E-01		
	rs7735553	chr5	73,418,739	365	426	131	405	391	136	0.928	2.90E-01	5.38E-01		
	rs17636389	chr5	73,418,871	9	150	763	9	149	774	1.02	9.20E-01	3.62E-02		
	rs4703619	chr5	73,420,482	147	462	313	182	413	336	0.97	6.80E-01	1.67E-01		
	rs2840108	chr5	73,428,125	213	482	227	243	430	258	1	1.00E+00	0.00E+00		
	rs4704108	chr5	73,428,422	79	379	464	84	354	494	1.06	4.70E-01	3.28E-01		
	rs10063644	chr5	73,430,875	145	456	321	178	412	341	0.968	6.50E-01	1.87E-01		
	rs17555606	chr5	73,435,599	9	144	768	7	136	787	1.1	4.60E-01	3.37E-01		
	rs288855	chr5	73,438,971	493	373	56	504	358	70	1.02	8.00E-01	9.69E-02		
	rs464577	chr5	73,447,915	494	372	56	503	358	70	1.03	7.60E-01	1.19E-01		
	rs10064733	chr5	73,455,822	93	421	408	113	374	445	1.03	6.60E-01	1.80E-01		
	rs10072489	chr5	73,455,899	406	422	94	446	374	112	0.956	5.40E-01	2.68E-01		
	rs169628	chr5	73,459,050	143	462	317	176	413	343	0.98	7.90E-01	1.02E-01		
	rs288864	chr5	73,463,799	188	472	258	238	436	255	0.89	8.40E-02	1.08E+00		
	rs158580	chr5	73,470,256	72	378	472	72	363	497	1.06	4.70E-01	3.28E-01		
	rs2120729	chr5	73,478,194	78	404	438	102	365	464	0.994	9.60E-01	1.77E-02		
	rs191899	chr5	73,481,573	347	436	139	361	429	142	0.98	8.00E-01	9.69E-02		
	rs10515174	chr5	73,483,134	842	78	1	862	68	2	0.885	5.10E-01	2.92E-01		
	rs12188714	chr5	73,486,196	605	282	35	634	256	42	0.946	5.30E-01	2.76E-01		
	rs12188320	chr5	73,488,183	113	412	396	126	407	399	0.969	6.70E-01	1.74E-01		
	rs17735123	chr5	73,488,223	658	240	24	640	262	30	1.13	1.90E-01	7.21E-01		
	rs290726	chr5	73,495,641	80	399	443	87	380	465	1.03	7.20E-01	1.43E-01		
	rs288837	chr5	73,499,743	59	376	487	76	341	515	1.02	8.40E-01	7.57E-02		
	rs1444565	chr5	73,506,496	379	434	108	399	418	115	0.977	7.60E-01	1.19E-01		
	rs158807	chr5	73,510,358	77	408	436	85	373	473	1.07	3.90E-01	4.09E-01		
	rs11745556	chr5	73,512,572	5	111	806	4	116	812	0.985	9.60E-01	1.77E-02		
	rs4704111	chr5	73,518,971	256	468	198	298	444	190	0.899	1.10E-01	9.59E-01		
	rs158805	chr5	73,522,406	476	384	62	511	353	68	0.935	3.90E-01	4.09E-01		
	rs716212	chr5	73,523,708	6	123	793	6	118	808	1.05	7.30E-01	1.37E-01		
	rs13169946	chr5	73,524,948	189	466	267	226	459	247	0.883	6.30E-02	1.20E+00		
	rs1444560	chr5	73,526,320	275	463	174	254	456	219	1.16	2.90E-02	1.54E+00		
	rs158815	chr5	73,533,753	547	323	52	574	312	46	0.918	3.00E-01	5.23E-01		
	rs10061061	chr5	73,538,940	177	454	287	206	473	249	0.862	2.70E-02	1.57E+00		
	rs7719793	chr5	73,541,155	820	98	3	835	96	0	0.908	5.60E-01	2.52E-01		
	rs158810	chr5	73,541,867	566	320	35	600	299	33	0.907	2.50E-01	6.02E-01		
	rs292203	chr5	73,542,540	79	401	442	75	381	476	1.09	2.40E-01	6.20E-01		
	rs167111	chr5	73,542,948	150	467	305	152	472	308	0.998	9.90E-01	4.36E-03		
	rs12519078	chr5	73,545,252	62	348	511	62	354	516	0.999	9.80E-01	8.77E-03		
	rs209770	chr5	73,545,509	460	374	88	463	392	77	0.975	7.50E-01	1.25E-01		
	rs2004500	chr5	73,546,408	188	437	297	208	443	281	0.923	2.40E-01	6.20E-01		
	rs680500	chr5	73,547,443	486	368	68	514	348	70	0.943	4.50E-01	3.47E-01		
	rs7728448	chr5	73,552,681	511	347	64	515	353	64	1	1.00E+00	0.00E+00		
	rs6453045	chr5	73,555,480	155	427	339	142	420	368	1.1	1.90E-01	7.21E-01		
	rs685295	chr5	73,556,393	335	417	170	337	434	161	0.98	7.90E-01	1.02E-01		
	rs6863889	chr5	73,560,159	800	120	2	820	106	6	0.937	6.80E-01	1.67E-01		
	rs4479825	chr5	73,563,084	6	173	743	7	169	756	1.02	8.70E-01	6.05E-02		
	rs10515165	chr5	73,563,580	196	483	243	226	474	232	0.915	1.90E-01	7.21E-01		
	rs1824495	chr5	73,572,506	387	400	135	393	438	101	0.916	2.20E-01	6.58E-01		
	rs627834	chr5	73,575,492	686	215	21	694	212	26	1.02	8.80E-01	5.55E-02		
	rs292213	chr5	73,579,347	46	367	509	74	364	494	0.875	8.10E-02	1.09E+00		
	rs654937	chr5	73,582,825	76	368	478	82	391	459	0.926	3.10E-01	5.09E-01		
	rs7723844	chr5	73,583,800	8	170	743	11	181	740	0.919	4.60E-01	3.37E-01		
	rs2962622	chr5	73,584,027	60	369	493	74	384	474	0.904	1.80E-01	7.45E-01		
	rs292209	chr5	73,590,100	482	366	74	457	383	92	1.13	9.50E-02	1.02E+00		
	rs11740435	chr5	73,591,760	799	119	4	779	150	3	1.23	1.00E-01	1.00E+00		
	rs7728483	chr5	73,592,316	16	222	683	20	229	683	0.948	6.10E-01	2.15E-01		
	rs1460806	chr5	73,593,425	211	442	269	223	473	235	0.905	1.40E-01	8.54E-01		
	rs2126039	chr5	73,600,305	29	259	634	28	265	639	0.998	9.80E-01	8.77E-03		
	rs17643294	chr5	73,601,431	515	344	63	506	361	65	1.05	5.80E-01	2.37E-01		
	rs569120	chr5	73,605,019	219	463	240	232	458	242	0.976	7.40E-01	1.31E-01		
	rs1870658	chr5	73,612,235	777	140	5	782	143	7	1.04	8.00E-01	9.69E-02		
	rs471405	chr5	73,613,440	5	140	777	7	143	782	0.963	8.00E-01	9.69E-02		
	rs2962624	chr5	73,614,274	172	459	291	182	426	324	1.05	4.90E-01	3.10E-01		
	rs10515167	chr5	73,616,240	427	410	85	471	386	75	0.88	7.90E-02	1.10E+00		
	rs922202	chr5	73,624,885	222	483	217	209	481	242	1.09	2.30E-01	6.38E-01		
	rs16871645	chr5	73,639,570	2	87	831	3	100	826	0.86	3.40E-01	4.69E-01		
	rs2872489	chr5	73,645,873	122	441	359	132	458	342	0.935	3.40E-01	4.69E-01		
	rs533886	chr5	73,654,548	9	178	735	8	183	741	0.995	9.90E-01	4.36E-03		
	rs12521529	chr5	73,654,575	355	444	123	343	457	132	1.05	4.50E-01	3.47E-01		

	rs2199316	chr5	73,655,326	89	417	416	94	428	410	0.965	6.40E-01	1.94E-01		
	rs4704129	chr5	73,655,459	245	474	203	234	487	211	1.04	5.50E-01	2.60E-01		
	rs4704133	chr5	73,667,082	94	419	409	93	431	408	0.992	9.30E-01	3.15E-02		
	rs4704134	chr5	73,669,592	152	472	298	155	473	304	1	9.90E-01	4.36E-03		
	rs376518	chr5	73,689,743	8	186	727	12	204	716	0.884	2.50E-01	6.02E-01		
	rs571408	chr5	73,690,403	8	187	727	12	204	716	0.888	2.70E-01	5.69E-01		
	rs1873363	chr5	73,692,476	749	162	10	762	159	11	0.981	9.10E-01	4.10E-02		
	rs409773	chr5	73,693,516	37	311	574	41	329	562	0.933	4.10E-01	3.87E-01		
	rs548734	chr5	73,704,090	92	415	413	93	419	420	1	9.80E-01	8.77E-03		
	rs10036629	chr5	73,706,989	191	445	285	178	471	283	1.02	7.70E-01	1.14E-01		
	rs10515178	chr5	73,709,913	124	412	386	126	401	405	1.03	6.50E-01	1.87E-01		
	rs540104	chr5	73,714,392	651	238	32	638	269	25	1.05	5.90E-01	2.29E-01		
	rs452853	chr5	73,715,141	32	239	650	26	268	638	0.95	5.90E-01	2.29E-01		
	rs9293625	chr5	73,715,681	149	411	362	135	428	369	1.04	5.50E-01	2.60E-01		
	rs7704601	chr5	73,718,020	214	434	268	212	430	281	1.03	6.50E-01	1.87E-01		
	rs294986	chr5	73,718,795	592	289	41	581	304	47	1.08	3.80E-01	4.20E-01		
	rs17558109	chr5	73,719,123	21	257	630	24	230	651	1.09	3.90E-01	4.09E-01		
	rs10454913	chr5	73,720,859	286	442	194	293	445	194	0.987	8.70E-01	6.05E-02		
	rs1846971	chr5	73,724,253	760	157	5	759	165	7	1.07	6.00E-01	2.22E-01		
	rs10065639	chr5	73,726,105	509	349	64	511	348	73	1.03	6.90E-01	1.61E-01		
	rs4704139	chr5	73,729,635	632	259	30	630	270	32	1.04	6.60E-01	1.80E-01		
	rs10474415	chr5	73,730,174	122	416	384	125	417	390	1	9.80E-01	8.77E-03		
	rs295691	chr5	73,738,066	364	410	148	362	436	134	0.978	7.70E-01	1.14E-01		
	rs464581	chr5	73,741,558	622	276	24	661	245	26	0.889	2.00E-01	6.99E-01		
	rs413430	chr5	73,746,180	761	155	6	752	168	12	1.15	2.20E-01	6.58E-01		
	rs435958	chr5	73,748,965	189	449	283	215	456	261	0.899	1.20E-01	9.21E-01		
	rs10515181	chr5	73,751,240	11	159	752	14	159	759	0.976	8.70E-01	6.05E-02		
	rs6889605	chr5	73,753,356	661	233	27	646	255	31	1.11	2.60E-01	5.85E-01		
	rs6863488	chr5	73,753,401	27	234	661	31	255	645	0.902	2.70E-01	5.69E-01		
	rs460738	chr5	73,755,416	343	410	165	354	427	145	0.936	3.40E-01	4.69E-01		
	rs417421	chr5	73,757,300	638	258	25	627	268	35	1.11	2.60E-01	5.85E-01		
	rs294983	chr5	73,757,965	105	385	432	126	405	401	0.875	6.00E-02	1.22E+00		
	rs16871870	chr5	73,778,817	711	191	20	728	188	16	0.934	5.30E-01	2.76E-01		
	rs445653	chr5	73,780,322	447	395	80	483	359	90	0.945	4.50E-01	3.47E-01		
	rs294971	chr5	73,780,816	133	395	393	139	431	362	0.912	1.90E-01	7.21E-01		
	rs296941	chr5	73,782,343	619	275	28	643	255	34	0.958	6.50E-01	1.87E-01		
	rs294975	chr5	73,783,037	190	447	285	193	429	310	1.05	5.10E-01	2.92E-01		
	rs1873365	chr5	73,785,648	869	38	3	869	56	3	1.39	1.20E-01	9.21E-01		
	rs2927624	chr5	73,793,817	665	234	23	687	226	19	0.922	4.10E-01	3.87E-01		
	rs17559038	chr5	73,802,706	782	135	4	811	116	4	0.848	2.20E-01	6.58E-01		
	rs7703491	chr5	73,803,337	644	250	28	677	232	23	0.881	1.70E-01	7.70E-01		
	rs6453059	chr5	73,803,891	45	365	510	59	340	532	1.01	9.60E-01	1.77E-02		
	rs6453061	chr5	73,807,422	304	442	176	296	458	176	1.02	7.90E-01	1.02E-01		
	rs12153302	chr5	73,813,134	30	230	662	16	263	652	0.991	9.60E-01	1.77E-02		
	rs2927632	chr5	73,814,412	70	359	493	86	357	489	0.936	3.90E-01	4.09E-01		
	rs2962189	chr5	73,814,530	21	269	632	28	270	634	0.957	6.50E-01	1.87E-01		
	rs2927633	chr5	73,816,540	168	444	310	189	470	273	0.878	5.40E-02	1.27E+00		
	rs6874566	chr5	73,822,636	500	351	71	491	361	80	1.06	4.30E-01	3.67E-01		
	rs1544939	chr5	73,823,034	499	350	72	488	364	80	1.07	4.00E-01	3.98E-01		
	rs890995	chr5	73,824,022	331	455	136	342	455	135	0.978	7.70E-01	1.14E-01		
	rs298380	chr5	73,829,765	522	340	55	528	327	73	1.05	5.30E-01	2.76E-01		
	rs6453065	chr5	73,832,280	22	283	617	21	247	664	1.17	7.50E-02	1.12E+00		
	rs11749231	chr5	73,835,839	587	309	26	631	274	27	0.877	1.30E-01	8.86E-01		
	rs298372	chr5	73,836,077	525	342	55	526	332	74	1.07	4.10E-01	3.87E-01		
	rs17645426	chr5	73,836,374	26	308	588	27	274	631	1.14	1.40E-01	8.54E-01		
	rs10053091	chr5	73,839,457	728	188	6	712	200	20	1.21	6.30E-02	1.20E+00		
	rs16871933	chr5	73,841,521	796	119	7	804	125	2	0.958	7.80E-01	1.08E-01		
	rs16871943	chr5	73,844,502	7	120	795	2	129	800	1.02	9.30E-01	3.15E-02		
	rs212925	chr5	73,846,234	205	463	254	228	442	262	0.967	6.30E-01	2.01E-01		
	rs11745925	chr5	73,847,006	59	380	483	55	356	520	1.11	1.80E-01	7.45E-01		
	rs186139	chr5	73,851,866	204	464	254	227	440	265	0.973	7.10E-01	1.49E-01		
	rs298393	chr5	73,856,998	205	464	253	229	439	264	0.971	6.80E-01	1.67E-01		
	rs16871987	chr5	73,867,959	7	143	772	5	152	775	0.978	8.90E-01	5.06E-02		
	rs1651349	chr5	73,869,708	113	408	401	127	411	394	0.945	4.30E-01	3.67E-01		
	rs1510936	chr5	73,869,868	507	354	61	515	335	82	1.05	5.30E-01	2.76E-01		
	rs390755	chr5	73,875,573	327	446	149	324	443	165	1.05	5.10E-01	2.92E-01		
	rs435624	chr5	73,877,583	277	462	183	307	445	180	0.933	3.10E-01	5.09E-01		
	rs13189660	chr5	73,877,692	437	393	92	456	395	81	0.936	3.70E-01	4.32E-01		
	rs424946	chr5	73,880,084	92	393	437	81	395	456	1.07	3.70E-01	4.32E-01		
	rs2173671	chr5	73,882,800	85	399	438	92	383	457	1.02	8.00E-01	9.69E-02		

	rs298389	chr5	73,884,748	44	319	550	67	321	534	0.876	9.60E-02	1.02E+00		
	rs1428558	chr5	73,887,534	121	459	342	160	443	329	0.885	7.40E-02	1.13E+00		
	rs298385	chr5	73,890,920	143	457	320	169	433	329	0.958	5.50E-01	2.60E-01		
	rs17560029	chr5	73,891,614	12	205	703	18	195	718	1	9.90E-01	4.36E-03		
	rs1428557	chr5	73,891,765	702	207	13	718	195	19	0.988	9.40E-01	2.69E-02		
	rs4704147	chr5	73,897,034	36	289	597	41	273	618	1.03	7.10E-01	1.49E-01		
	rs300248	chr5	73,914,951	501	365	56	539	337	56	0.909	2.20E-01	6.58E-01		
	rs300252	chr5	73,919,643	117	427	378	103	419	410	1.11	1.50E-01	8.24E-01		
	rs300245	chr5	73,922,447	242	474	206	272	444	216	0.959	5.40E-01	2.68E-01		
	rs17560266	chr5	73,929,865	746	169	7	767	159	6	0.917	4.70E-01	3.28E-01		
	rs300262	chr5	73,942,462	339	459	124	366	420	145	0.991	9.20E-01	3.62E-02		
	rs300261	chr5	73,943,589	493	369	59	525	348	59	0.927	3.30E-01	4.81E-01		
	rs10079609	chr5	73,954,372	711	200	11	702	211	19	1.13	2.50E-01	6.02E-01		
	rs300259	chr5	73,959,815	0	79	843	3	74	855	0.998	9.40E-01	2.69E-02		
	rs10075142	chr5	73,962,740	11	193	718	17	203	712	0.906	3.50E-01	4.56E-01		
	rs300240	chr5	73,966,198	387	416	119	404	405	123	0.977	7.60E-01	1.19E-01		
	rs10474420	chr5	73,970,030	711	200	11	709	206	17	1.08	4.70E-01	3.28E-01		
	rs1039321	chr5	73,973,262	546	321	55	567	317	48	0.933	4.00E-01	3.98E-01		
	rs7704467	chr5	73,979,865	110	411	401	118	399	415	1.01	9.50E-01	2.23E-02		
	rs6864196	chr5	73,980,200	706	204	12	711	205	16	1.03	7.90E-01	1.02E-01		
	rs2173673	chr5	73,981,854	546	321	55	563	314	55	0.965	6.80E-01	1.67E-01		
	rs9942341	chr5	73,982,920	60	324	538	50	331	547	1.05	5.70E-01	2.44E-01		
	rs13173835	chr5	73,983,347	129	416	377	137	409	386	0.996	9.80E-01	8.77E-03		
	rs10058135	chr5	73,984,765	777	139	6	777	146	9	1.08	5.40E-01	2.68E-01		
	rs4703638	chr5	73,987,489	56	274	588	56	328	545	0.858	5.80E-02	1.24E+00		
	rs12152861	chr5	73,990,375	264	445	213	238	466	228	1.09	1.90E-01	7.21E-01		
	rs16872154	chr5	73,991,036	39	325	558	55	316	561	0.944	4.90E-01	3.10E-01		
	rs4703640	chr5	73,994,001	73	333	516	80	360	492	0.907	2.00E-01	6.99E-01		
	rs2454847	chr5	73,995,388	67	349	506	64	312	556	1.15	7.40E-02	1.13E+00		
	rs820857	chr5	73,996,748	448	384	90	486	357	89	0.914	2.20E-01	6.58E-01		
	rs820853	chr5	73,998,262	335	419	168	323	450	159	1.01	9.00E-01	4.58E-02		
	rs820848	chr5	74,000,416	441	379	102	482	359	91	0.884	9.30E-02	1.03E+00		
	rs820846	chr5	74,001,266	163	420	339	155	451	326	0.985	8.40E-01	7.57E-02		
	rs6453072	chr5	74,002,657	267	472	183	270	478	184	0.998	1.00E+00	0.00E+00		
	rs820844	chr5	74,003,720	156	436	330	171	462	299	0.9	1.20E-01	9.21E-01		
	rs16872173	chr5	74,004,814	706	199	17	705	212	15	1.03	7.90E-01	1.02E-01		
	rs820841	chr5	74,006,353	254	473	192	281	461	188	0.937	3.40E-01	4.69E-01		
	rs820840	chr5	74,006,863	158	439	324	152	427	353	1.08	2.90E-01	5.38E-01		
	rs11749103	chr5	74,007,533	710	201	11	721	195	16	1.01	9.80E-01	8.77E-03		
	rs11748460	chr5	74,010,243	3	106	813	7	102	823	0.974	9.00E-01	4.58E-02		
	rs3846656	chr5	74,013,224	0	1	921	0	0	932	0	5.00E-01	3.01E-01		
	rs820870	chr5	74,015,867	279	469	174	298	465	169	0.951	4.70E-01	3.28E-01		
	rs820880	chr5	74,018,081	474	382	65	516	351	65	0.903	1.80E-01	7.45E-01		
	rs11556045	chr5	74,020,971	592	302	28	641	265	26	0.851	6.30E-02	1.20E+00		
	rs820889	chr5	74,021,290	3	148	771	12	139	781	0.951	7.10E-01	1.49E-01		
	rs3756443	chr5	74,023,302	590	303	28	641	265	26	0.846	5.60E-02	1.25E+00		
	rs3756444	chr5	74,023,531	380	422	117	402	423	105	0.93	3.10E-01	5.09E-01		
	rs1696979	chr5	74,028,162	131	440	345	141	446	337	0.956	5.30E-01	2.76E-01		
	rs10805890	chr5	74,028,637	648	249	25	643	261	28	1.06	5.50E-01	2.60E-01		
	rs820834	chr5	74,034,961	5	149	768	9	156	767	0.917	4.80E-01	3.19E-01		
	rs820827	chr5	74,037,733	740	170	8	723	197	10	1.17	1.50E-01	8.24E-01		
	rs17561000	chr5	74,045,083	770	142	10	772	153	7	1.02	9.00E-01	4.58E-02		
	rs6453074	chr5	74,049,367	46	309	561	42	284	597	1.13	1.60E-01	7.96E-01		
	rs6867972	chr5	74,051,106	334	434	153	311	464	156	1.06	3.70E-01	4.32E-01		
	rs7705519	chr5	74,092,269	688	205	26	702	209	21	0.957	6.80E-01	1.67E-01		
	rs11948188	chr5	74,093,839	56	305	559	48	330	552	0.986	8.90E-01	5.06E-02		
	rs10942719	chr5	74,094,154	328	429	165	335	429	167	0.992	9.40E-01	2.69E-02		
	rs10515193	chr5	74,094,847	712	202	8	699	215	18	1.16	1.50E-01	8.24E-01		
	rs1164694	chr5	74,102,142	857	61	4	850	81	1	1.2	3.10E-01	5.09E-01		
	rs6453083	chr5	74,111,114	117	402	394	115	435	368	0.941	4.00E-01	3.98E-01		
	rs10044287	chr5	74,131,298	607	277	38	588	296	47	1.12	1.80E-01	7.45E-01		
	rs6866082	chr5	74,132,165	55	305	562	46	330	556	0.992	9.50E-01	2.23E-02		
	rs6881400	chr5	74,152,776	712	194	16	738	179	15	0.904	3.50E-01	4.56E-01		
	rs6877116	chr5	74,155,058	43	290	555	39	298	564	1.02	8.50E-01	7.06E-02		
	rs2218517	chr5	74,157,402	56	332	534	46	329	557	1.09	3.00E-01	5.23E-01		
	rs1164624	chr5	74,178,621	318	439	165	290	471	171	1.08	2.50E-01	6.02E-01		
	rs17738565	chr5	74,179,544	765	149	5	767	154	9	1.08	5.60E-01	2.52E-01		
	rs10062244	chr5	74,204,801	1	144	777	8	150	774	0.88	3.10E-01	5.09E-01		
	rs11739301	chr5	74,208,224	33	274	615	31	269	632	1.05	6.20E-01	2.08E-01		
	rs10942722	chr5	74,219,900	338	446	138	347	442	143	0.996	9.80E-01	8.77E-03		

	rs12519932	chr5	74,233,709	439	406	76	449	398	85	1.01	9.30E-01	3.15E-02		
	rs11745686	chr5	74,234,367	441	406	75	450	397	85	1.01	8.90E-01	5.06E-02		
	rs11746561	chr5	74,248,684	34	278	610	32	272	628	1.05	5.90E-01	2.29E-01		
	rs7722269	chr5	74,251,060	8	190	722	14	203	714	0.89	2.80E-01	5.53E-01		
	rs6878227	chr5	74,257,948	65	356	501	74	343	515	1	9.80E-01	8.77E-03		
	rs6453096	chr5	74,272,205	750	156	16	768	152	9	0.887	3.10E-01	5.09E-01		
	rs7728102	chr5	74,275,132	568	299	55	596	299	37	0.878	1.10E-01	9.59E-01		
	rs9293636	chr5	74,275,702	15	191	716	9	181	742	1.14	2.30E-01	6.38E-01		
	rs3934476	chr5	74,278,608	287	460	175	289	473	170	0.987	8.70E-01	6.05E-02		
	rs7356745	chr5	74,278,876	728	182	12	737	183	12	0.993	9.90E-01	4.36E-03		
	rs6453099	chr5	74,295,306	149	434	339	145	453	334	0.993	9.50E-01	2.23E-02		
	rs4392601	chr5	74,298,736	504	357	61	515	351	66	0.997	9.90E-01	4.36E-03		
	rs10069744	chr5	74,299,211	749	160	13	751	168	13	1.04	7.90E-01	1.02E-01		
	rs10043960	chr5	74,302,099	543	323	56	544	336	52	1	9.80E-01	8.77E-03		
	rs13182265	chr5	74,305,480	27	236	659	23	254	655	0.973	7.90E-01	1.02E-01		
	rs6870053	chr5	74,312,322	353	433	136	356	449	126	0.975	7.40E-01	1.31E-01		
	rs6867482	chr5	74,316,736	179	438	305	169	474	289	0.984	8.30E-01	8.09E-02		
	rs7707671	chr5	74,323,498	454	370	98	455	386	91	0.99	9.10E-01	4.10E-02		
	rs6453104	chr5	74,324,825	347	425	148	339	453	135	0.992	9.30E-01	3.15E-02		
	rs6894634	chr5	74,336,369	56	360	506	62	369	501	0.957	5.80E-01	2.37E-01		
	rs12651778	chr5	74,337,389	5	115	802	5	126	801	0.924	5.80E-01	2.37E-01		
	rs7721887	chr5	74,337,608	775	142	4	774	152	6	1.09	5.10E-01	2.92E-01		
	rs3811986	chr5	74,360,193	55	366	501	60	374	498	0.965	6.60E-01	1.80E-01		
	rs4704166	chr5	74,360,658	54	367	501	60	374	498	0.962	6.30E-01	2.01E-01		
	rs7734427	chr5	74,363,824	55	366	501	60	374	498	0.965	6.60E-01	1.80E-01		
	rs11955513	chr5	74,372,639	8	151	763	7	148	776	1.05	7.50E-01	1.25E-01		
	rs7714420	chr5	74,376,083	480	371	71	478	391	63	0.996	9.80E-01	8.77E-03		
	rs9293641	chr5	74,381,125	14	151	757	10	153	769	1.05	7.00E-01	1.55E-01		
	rs7700965	chr5	74,392,613	381	425	116	407	418	107	0.927	2.90E-01	5.38E-01		
	rs3923323	chr5	74,407,897	649	240	33	667	243	22	0.915	3.40E-01	4.69E-01		
	rs4345300	chr5	74,412,327	117	424	381	108	418	406	1.08	3.00E-01	5.23E-01		
	rs4301212	chr5	74,444,735	24	239	657	20	237	671	1.05	6.00E-01	2.22E-01		
	rs2035191	chr5	74,449,469	637	252	33	651	258	22	0.929	4.30E-01	3.67E-01		
	rs6453116	chr5	74,457,542	721	190	11	748	174	10	0.894	3.10E-01	5.09E-01		
	rs717587	chr5	74,469,591	716	188	11	746	169	10	0.878	2.40E-01	6.20E-01		
	rs1422698	chr5	74,478,888	115	419	375	105	412	402	1.09	2.50E-01	6.02E-01		
	rs10054860	chr5	74,482,560	571	307	44	586	304	42	0.964	6.80E-01	1.67E-01		
	rs7378985	chr5	74,490,823	11	175	736	10	157	765	1.14	2.50E-01	6.02E-01		
	rs6872586	chr5	74,492,233	13	208	699	13	184	733	1.14	2.00E-01	6.99E-01		
	rs9293646	chr5	74,495,327	886	36	0	892	40	0	1.1	7.60E-01	1.19E-01		
	rs7726659	chr5	74,513,834	897	22	0	915	17	0	0.76	4.90E-01	3.10E-01		
	rs2219745	chr5	74,542,414	44	307	569	43	302	585	1.04	6.80E-01	1.67E-01		
	rs6866661	chr5	74,570,128	375	428	119	398	422	112	0.938	3.70E-01	4.32E-01		
	rs1551894	chr5	74,606,287	38	307	577	36	307	589	1.03	7.70E-01	1.14E-01		
	rs1423528	chr5	74,632,733	826	93	0	825	103	3	1.17	3.20E-01	4.95E-01		
	rs2335418	chr5	74,639,235	166	431	325	151	471	310	0.996	9.80E-01	8.77E-03		
	rs4703666	chr5	74,642,616	830	91	1	829	99	4	1.15	3.90E-01	4.09E-01		
	rs13358429	chr5	74,653,942	104	395	408	99	403	417	1.02	7.50E-01	1.25E-01		
	rs13354746	chr5	74,654,888	40	323	550	39	319	560	1.03	7.70E-01	1.14E-01		
	rs3761739	chr5	74,667,257	13	224	685	21	243	668	0.869	1.50E-01	8.24E-01		
	rs3761740	chr5	74,667,889	4	150	768	9	162	761	0.877	2.70E-01	5.69E-01		
	rs2303152	chr5	74,677,463	8	160	754	12	165	753	0.933	5.70E-01	2.44E-01		
	rs11742194	chr5	74,682,634	3	151	757	7	162	750	0.89	3.40E-01	4.69E-01		
	rs3846662	chr5	74,686,840	315	439	168	306	470	156	0.997	9.90E-01	4.36E-03		
	rs5909	chr5	74,691,931	3	151	768	7	164	761	0.881	3.00E-01	5.23E-01		
	rs16872536	chr5	74,714,241	4	146	772	10	150	772	0.908	4.40E-01	3.57E-01		
	rs3761742	chr5	74,714,693	31	306	562	36	308	573	0.985	8.80E-01	5.55E-02		
	rs6453133	chr5	74,728,532	462	376	79	461	399	70	0.993	9.60E-01	1.77E-02		
	rs4385188	chr5	74,770,118	525	350	47	548	334	50	0.957	6.00E-01	2.22E-01		
	rs6872314	chr5	74,771,821	525	350	47	549	332	51	0.957	6.00E-01	2.22E-01		
	rs12659791	chr5	74,793,514	683	224	13	665	244	22	1.16	1.10E-01	9.59E-01		
	rs6896136	chr5	74,823,066	361	427	134	338	465	129	1.05	5.10E-01	2.92E-01		
	rs10055011	chr5	74,833,912	12	212	697	12	192	727	1.12	2.80E-01	5.53E-01		
	rs5744545	chr5	74,844,173	10	191	721	9	181	742	1.08	4.90E-01	3.10E-01		
	rs3756558	chr5	74,898,797	680	228	14	664	248	20	1.13	1.90E-01	7.21E-01		
	rs3097154	chr5	74,906,574	919	2	0	932	0	0	0	2.50E-01	6.02E-01		
	rs5744661	chr5	74,910,746	4	145	773	10	150	772	0.902	4.10E-01	3.87E-01		
	rs3213801	chr5	74,913,022	9	154	751	10	189	728	0.817	7.10E-02	1.15E+00		
	rs5744680	chr5	74,915,646	132	428	362	121	467	344	0.979	7.70E-01	1.14E-01		
	rs5744707	chr5	74,926,374	762	156	4	755	170	7	1.12	3.30E-01	4.81E-01		

	rs1051795	chr5	74,932,554	37	317	568	37	313	582	1.03	7.70E-01	1.14E-01		
	rs904743	chr5	74,953,618	688	219	15	716	202	14	0.902	3.10E-01	5.09E-01		
	rs16872661	chr5	74,959,688	1	46	874	1	43	888	1.08	7.90E-01	1.02E-01		
	rs924452	chr5	74,959,712	36	320	565	37	310	581	1.04	6.90E-01	1.61E-01		
	rs253407	chr5	74,962,534	68	380	474	74	359	499	1.04	6.20E-01	2.08E-01		
	rs34344	chr5	74,971,581	60	372	485	70	339	511	1.04	6.10E-01	2.15E-01		
	rs16872693	chr5	74,982,211	1	50	871	2	43	887	1.12	6.40E-01	1.94E-01		
	rs16872698	chr5	74,983,191	874	47	1	887	43	2	0.948	8.80E-01	5.55E-02		
	rs10942740	chr5	74,984,799	921	1	0	932	0	0	0	5.00E-01	3.01E-01		
	rs7717355	chr5	74,987,657	11	142	769	10	153	769	0.954	7.20E-01	1.43E-01		
	rs253414	chr5	74,992,273	420	406	96	427	405	96	0.988	8.90E-01	5.06E-02		
	rs16872726	chr5	74,994,052	1	59	862	1	63	863	0.942	8.10E-01	9.15E-02		
	rs34355	chr5	74,995,726	601	283	38	598	294	40	1.04	6.80E-01	1.67E-01		
	rs6453138	chr5	74,996,114	921	0	1	930	2	0	0.989	1.00E+00	0.00E+00		
	rs6453139	chr5	74,998,524	4	81	837	4	90	838	0.914	6.00E-01	2.22E-01		
	rs11750595	chr5	74,999,356	807	105	7	822	107	3	0.932	6.50E-01	1.87E-01		
	rs34358	chr5	75,000,878	409	414	99	408	416	108	1.03	6.70E-01	1.74E-01		
	rs10056022	chr5	75,005,171	7	111	804	4	111	817	1.07	6.80E-01	1.67E-01		
	rs17648910	chr5	75,006,312	814	103	5	824	105	3	0.97	8.80E-01	5.55E-02		
	rs888786	chr5	75,006,606	22	208	692	21	225	686	0.947	6.00E-01	2.22E-01		
	rs2303647	chr5	75,009,635	7	147	768	10	148	773	0.965	8.00E-01	9.69E-02		
	rs253388	chr5	75,011,668	70	342	507	66	355	509	1	9.90E-01	4.36E-03		
	rs253390	chr5	75,013,417	72	351	497	72	355	505	1.01	9.60E-01	1.77E-02		
	rs194137	chr5	75,014,613	499	351	72	506	354	72	0.994	9.60E-01	1.77E-02		
	rs17649248	chr5	75,034,182	41	289	592	40	297	595	0.993	9.70E-01	1.32E-02		
	rs2307111	chr5	75,039,434	345	438	138	354	431	146	1	9.90E-01	4.36E-03		
	rs17649332	chr5	75,039,634	591	289	41	594	297	40	1.01	9.70E-01	1.32E-02		
	rs4640771	chr5	75,047,529	0	1	921	0	0	931	0	5.00E-01	3.01E-01		
	rs7730317	chr5	75,048,073	6	146	770	10	146	776	0.959	7.60E-01	1.19E-01		
	rs2291631	chr5	75,048,792	4	79	838	4	88	839	0.912	5.90E-01	2.29E-01		
	rs6867195	chr5	75,051,345	0	1	920	0	0	931	0	5.00E-01	3.01E-01		
	rs7715806	chr5	75,070,143	132	417	373	122	420	390	1.06	4.30E-01	3.67E-01		
	rs1001935	chr5	75,075,936	6	145	771	4	124	804	1.22	1.20E-01	9.21E-01		
	rs258497	chr5	75,080,441	180	457	284	161	452	319	1.12	8.80E-02	1.06E+00		
	rs795024	chr5	75,109,450	280	461	181	322	442	168	0.889	8.10E-02	1.09E+00		
	rs7725316	chr5	75,132,315	124	438	360	127	412	393	1.07	3.70E-01	4.32E-01		
	rs1525774	chr5	75,133,141	193	455	271	176	437	319	1.15	3.90E-02	1.41E+00		
	rs1464919	chr5	75,139,565	421	410	91	458	382	92	0.922	2.70E-01	5.69E-01		
	rs7709242	chr5	75,147,153	421	409	91	458	382	92	0.923	2.70E-01	5.69E-01		
	rs2049495	chr5	75,158,571	75	390	456	73	361	494	1.1	1.90E-01	7.21E-01		
	rs13158199	chr5	75,164,186	287	478	156	332	444	156	0.909	1.60E-01	7.96E-01		
	rs2049494	chr5	75,170,912	158	476	288	156	443	333	1.11	1.40E-01	8.54E-01		
	rs6891632	chr5	75,174,199	265	487	169	304	457	171	0.925	2.50E-01	6.02E-01		
	rs1546791	chr5	75,179,469	722	188	12	734	182	16	0.998	9.70E-01	1.32E-02		
	rs10805894	chr5	75,182,012	169	485	266	170	456	303	1.08	2.60E-01	5.85E-01		
	rs10515208	chr5	75,195,675	659	238	21	668	238	22	0.996	1.00E+00	0.00E+00		
	rs6891276	chr5	75,196,222	21	240	658	23	240	667	0.997	9.90E-01	4.36E-03		
	rs1464914	chr5	75,209,785	534	340	48	553	326	53	0.974	7.70E-01	1.14E-01		
	rs6859261	chr5	75,210,354	7	153	762	7	147	778	1.05	7.00E-01	1.55E-01		
	rs6860538	chr5	75,210,382	9	161	752	9	153	770	1.06	6.20E-01	2.08E-01		
	rs10474452	chr5	75,211,038	214	498	210	250	460	222	0.95	4.50E-01	3.47E-01		
	rs1357999	chr5	75,215,969	777	138	7	793	133	6	0.939	6.50E-01	1.87E-01		
	rs1404989	chr5	75,220,176	695	211	16	711	202	19	0.974	8.20E-01	8.62E-02		
	rs4704262	chr5	75,227,206	481	368	70	507	350	72	0.948	5.00E-01	3.01E-01		
	rs1525763	chr5	75,236,640	896	25	1	908	23	1	0.915	8.60E-01	6.55E-02		
	rs2222624	chr5	75,246,011	788	128	6	803	123	6	0.95	7.30E-01	1.37E-01		
	rs17650177	chr5	75,250,155	15	223	684	18	211	703	1.04	7.10E-01	1.49E-01		
	rs6863177	chr5	75,251,076	9	136	777	6	132	794	1.09	5.20E-01	2.84E-01		
	rs17564993	chr5	75,251,726	39	315	567	45	291	596	1.06	5.30E-01	2.76E-01		
	rs10942747	chr5	75,252,167	293	482	147	323	465	144	0.933	3.10E-01	5.09E-01		
	rs1917759	chr5	75,256,689	42	321	558	45	298	588	1.07	4.20E-01	3.77E-01		
	rs4487476	chr5	75,265,370	246	462	213	233	467	232	1.07	3.10E-01	5.09E-01		
	rs7729037	chr5	75,266,283	59	373	490	80	383	469	0.883	9.60E-02	1.02E+00		
	rs10076338	chr5	75,266,403	36	296	590	37	284	611	1.05	5.90E-01	2.29E-01		
	rs1357997	chr5	75,268,145	234	466	222	222	470	240	1.07	3.40E-01	4.69E-01		
	rs6453180	chr5	75,269,863	245	463	213	232	468	232	1.07	3.10E-01	5.09E-01		
	rs10075075	chr5	75,274,068	592	291	39	613	283	36	0.94	4.80E-01	3.19E-01		
	rs6895132	chr5	75,274,886	547	325	43	572	308	49	0.965	6.90E-01	1.61E-01		
	rs1917757	chr5	75,283,433	8	160	754	5	171	756	0.981	9.10E-01	4.10E-02		
	rs4704273	chr5	75,290,699	400	403	119	422	432	78	0.865	4.10E-02	1.39E+00		

	rs11747018	chr5	75,296,268	352	418	152	329	464	139	1.03	7.10E-01	1.49E-01		
	rs11740550	chr5	75,306,396	141	420	360	133	456	342	0.972	7.00E-01	1.55E-01		
	rs7731991	chr5	75,309,030	1	8	913	0	8	923	1.26	8.00E-01	9.69E-02		
	rs12055083	chr5	75,317,053	7	156	759	8	164	760	0.95	6.90E-01	1.61E-01		
	rs6414964	chr5	75,318,788	70	327	525	45	340	547	1.13	1.20E-01	9.21E-01		
	rs4580755	chr5	75,321,903	338	444	139	338	443	151	1.03	6.60E-01	1.80E-01		
	rs12657643	chr5	75,327,573	698	210	14	706	211	15	1	9.80E-01	8.77E-03		
	rs4425488	chr5	75,330,102	221	427	274	180	469	283	1.11	1.10E-01	9.59E-01		
	rs6864642	chr5	75,334,249	567	308	47	597	311	24	0.856	6.10E-02	1.21E+00		
	rs11750465	chr5	75,335,980	222	424	275	180	470	281	1.11	1.30E-01	8.86E-01		
	rs6871717	chr5	75,338,063	174	416	332	133	455	343	1.12	9.90E-02	1.00E+00		
	rs4235691	chr5	75,341,330	324	431	163	315	452	160	1.02	8.30E-01	8.09E-02		
	rs4704278	chr5	75,347,523	168	428	305	166	459	288	0.963	6.00E-01	2.22E-01		
	rs4374745	chr5	75,351,083	328	433	161	315	454	163	1.04	6.00E-01	2.22E-01		
	rs9293664	chr5	75,355,790	65	322	534	58	337	535	1.01	9.30E-01	3.15E-02		
	rs1553311	chr5	75,356,685	609	288	25	615	286	31	1.02	8.20E-01	8.62E-02		
	rs1910051	chr5	75,361,035	189	455	276	196	459	276	0.983	8.20E-01	8.62E-02		
	rs10044546	chr5	75,373,350	25	283	614	31	285	616	0.963	6.90E-01	1.61E-01		
	rs10515216	chr5	75,373,492	187	456	279	195	459	278	0.979	7.70E-01	1.14E-01		
	rs10042012	chr5	75,375,455	25	279	607	31	282	610	0.962	6.80E-01	1.67E-01		
	rs258622	chr5	75,424,590	189	474	259	187	461	284	1.06	4.10E-01	3.87E-01		
	rs1423101	chr5	75,431,572	65	326	531	58	333	539	1.03	7.00E-01	1.55E-01		
	rs2081076	chr5	75,437,493	920	2	0	931	1	0	0.494	6.20E-01	2.08E-01		
	rs17673786	chr5	75,439,893	607	288	27	612	290	30	1.02	8.90E-01	5.06E-02		
	rs187727	chr5	75,439,941	71	353	498	64	363	504	1.02	7.70E-01	1.14E-01		
	rs13160383	chr5	75,445,309	38	306	578	39	314	577	0.978	8.20E-01	8.62E-02		
	rs10054931	chr5	75,446,267	27	283	604	31	286	610	0.978	8.30E-01	8.09E-02		
	rs183766	chr5	75,446,387	421	410	91	436	399	97	0.987	8.80E-01	5.55E-02		
	rs10043369	chr5	75,447,560	29	285	608	31	289	612	0.985	8.90E-01	5.06E-02		
	rs10434645	chr5	75,457,862	641	256	24	630	274	28	1.09	3.50E-01	4.56E-01		
	rs1021782	chr5	75,459,306	312	442	168	324	434	174	0.99	9.10E-01	4.10E-02		
	rs10514059	chr5	75,460,983	408	401	113	392	423	117	1.06	4.50E-01	3.47E-01		
	rs2112865	chr5	75,465,359	788	132	2	789	138	5	1.08	5.60E-01	2.52E-01		
	rs12657223	chr5	75,467,195	540	320	61	530	341	60	1.04	6.20E-01	2.08E-01		
	rs10514061	chr5	75,471,124	25	235	658	23	227	681	1.07	4.90E-01	3.10E-01		
	rs7701465	chr5	75,472,145	172	439	310	188	436	306	0.954	5.00E-01	3.01E-01		
	rs6453205	chr5	75,474,978	325	433	164	319	432	181	1.06	4.30E-01	3.67E-01		
	rs10051982	chr5	75,477,632	6	107	807	6	103	822	1.05	7.70E-01	1.14E-01		
	rs1895391	chr5	75,481,681	218	453	249	224	453	249	0.987	8.60E-01	6.55E-02		
	rs6879305	chr5	75,485,883	219	482	221	227	459	246	1.04	6.00E-01	2.22E-01		
	rs6887093	chr5	75,491,007	4	100	811	5	109	811	0.912	5.50E-01	2.60E-01		
	rs13167138	chr5	75,494,053	23	234	665	24	222	686	1.06	5.80E-01	2.37E-01		
	rs4610441	chr5	75,494,157	289	472	160	305	463	164	0.977	7.50E-01	1.25E-01		
	rs4704283	chr5	75,506,572	142	467	313	151	456	325	1	1.00E+00	0.00E+00		
	rs1553325	chr5	75,507,473	312	466	144	325	455	152	0.993	9.40E-01	2.69E-02		
	rs9293675	chr5	75,510,536	312	464	144	325	455	152	0.994	9.50E-01	2.23E-02		
	rs2055439	chr5	75,511,014	286	462	174	294	441	197	1.04	6.20E-01	2.08E-01		
	rs2358531	chr5	75,515,542	481	385	55	498	360	74	1.02	8.20E-01	8.62E-02		
	rs1002541	chr5	75,518,658	301	466	155	327	432	173	0.986	8.60E-01	6.55E-02		
	rs12655684	chr5	75,528,458	43	258	621	34	259	639	1.08	4.00E-01	3.98E-01		
	rs7448529	chr5	75,530,752	180	443	299	190	472	270	0.916	2.00E-01	6.99E-01		
	rs11741865	chr5	75,532,899	4	131	787	6	138	788	0.932	6.10E-01	2.15E-01		
	rs9293679	chr5	75,538,611	110	412	393	103	447	378	0.972	7.10E-01	1.49E-01		
	rs17566351	chr5	75,539,999	44	301	577	38	271	623	1.17	6.40E-02	1.19E+00		
	rs4703700	chr5	75,544,306	68	376	478	65	366	501	1.06	4.50E-01	3.47E-01		
	rs6870971	chr5	75,554,185	109	434	376	110	411	411	1.07	3.20E-01	4.95E-01		
	rs981113	chr5	75,556,684	151	470	301	157	452	323	1.03	6.60E-01	1.80E-01		
	rs1532696	chr5	75,559,991	743	168	11	712	204	16	1.26	2.80E-02	1.55E+00		
	rs7444555	chr5	75,561,170	106	421	395	98	420	414	1.06	4.30E-01	3.67E-01		
	rs11750299	chr5	75,562,678	126	417	379	125	406	401	1.05	5.10E-01	2.92E-01		
	rs10514063	chr5	75,566,250	130	421	371	137	414	381	1	9.80E-01	8.77E-03		
	rs11959133	chr5	75,566,860	0	14	908	0	31	901	0.452	1.80E-02	1.74E+00		
	rs1532699	chr5	75,570,326	370	420	132	381	412	139	0.997	9.90E-01	4.36E-03		
	rs11750504	chr5	75,576,597	380	414	126	399	408	124	0.959	5.60E-01	2.52E-01		
	rs6879020	chr5	75,580,791	275	455	192	269	459	204	1.04	5.60E-01	2.52E-01		
	rs10075253	chr5	75,584,002	24	225	666	24	222	681	1.03	8.00E-01	9.69E-02		
	rs6891999	chr5	75,585,263	555	314	53	560	314	58	1.02	8.60E-01	6.55E-02		
	rs12654150	chr5	75,586,081	439	390	93	471	373	88	0.919	2.50E-01	6.02E-01		
	rs6862529	chr5	75,586,965	195	448	278	202	453	277	0.981	7.90E-01	1.02E-01		
	rs10462538	chr5	75,587,861	89	373	460	75	363	494	1.12	1.20E-01	9.21E-01		

	rs4481336	chr5	75,597,667	48	279	595	43	278	611	1.05	5.70E-01	2.44E-01		
	rs10064140	chr5	75,598,554	89	373	460	79	404	449	0.987	8.90E-01	5.06E-02		
	rs2972827	chr5	75,598,829	49	279	594	45	279	607	1.04	6.60E-01	1.80E-01		
	rs2937720	chr5	75,599,937	91	389	441	103	369	460	1.01	9.50E-01	2.23E-02		
	rs2937719	chr5	75,600,193	242	445	235	244	442	246	1.02	7.90E-01	1.02E-01		
	rs2972831	chr5	75,602,262	9	178	734	10	190	731	0.937	5.70E-01	2.44E-01		
	rs2937715	chr5	75,603,809	364	449	109	385	428	119	0.981	8.10E-01	9.15E-02		
	rs16873292	chr5	75,603,935	795	122	4	785	144	3	1.15	2.80E-01	5.53E-01		
	rs2937748	chr5	75,607,788	774	142	6	792	137	3	0.912	4.80E-01	3.19E-01		
	rs6874435	chr5	75,610,611	708	202	12	717	199	16	1.01	9.40E-01	2.69E-02		
	rs4566770	chr5	75,612,069	12	201	709	14	199	719	1	9.80E-01	8.77E-03		
	rs2358712	chr5	75,616,443	775	142	5	797	132	3	0.89	3.70E-01	4.32E-01		
	rs17566680	chr5	75,622,692	33	315	574	44	302	585	0.983	8.60E-01	6.55E-02		
	rs17747572	chr5	75,624,777	756	149	5	778	134	8	0.927	5.60E-01	2.52E-01		
	rs246813	chr5	75,635,361	5	142	775	3	132	797	1.12	3.70E-01	4.32E-01		
	rs2937746	chr5	75,638,459	761	154	7	787	141	4	0.867	2.50E-01	6.02E-01		
	rs2972844	chr5	75,640,607	298	455	169	283	476	173	1.05	5.20E-01	2.84E-01		
	rs4703705	chr5	75,642,464	461	384	77	440	404	88	1.1	2.10E-01	6.78E-01		
	rs2913261	chr5	75,644,749	342	445	135	340	445	147	1.04	6.10E-01	2.15E-01		
	rs2913257	chr5	75,648,088	762	152	7	787	141	4	0.877	2.90E-01	5.38E-01		
	rs10062226	chr5	75,650,493	2	64	856	2	74	856	0.877	4.90E-01	3.10E-01		
	rs3733860	chr5	75,658,570	20	210	692	19	200	713	1.07	5.10E-01	2.92E-01		
	rs2913251	chr5	75,659,445	129	418	375	115	424	393	1.07	3.30E-01	4.81E-01		
	rs6892721	chr5	75,659,531	70	356	496	73	360	499	0.988	8.90E-01	5.06E-02		
	rs17566917	chr5	75,661,030	548	311	62	518	351	63	1.11	1.70E-01	7.70E-01		
	rs31270	chr5	75,661,170	303	452	165	333	452	147	0.903	1.30E-01	8.86E-01		
	rs31269	chr5	75,661,708	237	449	235	249	469	214	0.932	3.00E-01	5.23E-01		
	rs10805900	chr5	75,662,126	643	245	30	674	235	23	0.891	2.20E-01	6.58E-01		
	rs16873320	chr5	75,663,745	863	59	0	865	64	3	1.18	4.00E-01	3.98E-01		
	rs31266	chr5	75,664,169	216	447	259	193	462	277	1.09	2.00E-01	6.99E-01		
	rs246819	chr5	75,673,318	289	454	177	295	464	173	0.982	8.00E-01	9.69E-02		
	rs246804	chr5	75,678,242	175	459	287	174	465	293	1.01	8.80E-01	5.55E-02		
	rs246801	chr5	75,681,754	165	442	315	165	467	300	0.964	6.10E-01	2.15E-01		
	rs246797	chr5	75,683,386	103	404	415	97	417	418	1.01	8.70E-01	6.05E-02		
	rs11748035	chr5	75,687,542	769	143	10	760	160	12	1.13	3.10E-01	5.09E-01		
	rs246789	chr5	75,699,372	34	281	605	32	285	608	1.01	9.70E-01	1.32E-02		
	rs246784	chr5	75,705,230	613	279	28	619	281	31	1.01	9.00E-01	4.58E-02		
	rs10514065	chr5	75,711,075	93	392	437	107	401	424	0.927	3.00E-01	5.23E-01		
	rs1811340	chr5	75,712,414	70	368	484	79	372	481	0.957	5.70E-01	2.44E-01		
	rs890708	chr5	75,719,916	142	422	355	124	424	384	1.11	1.50E-01	8.24E-01		
	rs16873342	chr5	75,720,845	914	8	0	928	4	0	0.494	2.60E-01	5.85E-01		
	rs875543	chr5	75,728,732	710	193	19	695	220	17	1.1	3.50E-01	4.56E-01		
	rs1984636	chr5	75,734,047	712	192	18	696	219	17	1.11	3.00E-01	5.23E-01		
	rs3886043	chr5	75,739,841	237	473	212	229	470	233	1.06	3.60E-01	4.44E-01		
	rs4296785	chr5	75,746,539	30	246	646	28	271	633	0.935	4.70E-01	3.28E-01		
	rs11749318	chr5	75,758,425	224	461	235	252	466	214	0.9	1.20E-01	9.21E-01		
	rs11741153	chr5	75,760,741	728	180	14	719	200	13	1.09	4.50E-01	3.47E-01		
	rs13183341	chr5	75,763,487	112	429	381	121	446	363	0.934	3.30E-01	4.81E-01		
	rs4130148	chr5	75,775,087	728	179	15	720	198	14	1.08	4.90E-01	3.10E-01		
	rs7730891	chr5	75,776,998	898	23	1	911	21	0	0.829	6.30E-01	2.01E-01		
	rs6880993	chr5	75,782,569	3	117	802	2	126	804	0.953	7.60E-01	1.19E-01		
	rs6898214	chr5	75,787,217	219	437	266	191	460	281	1.1	1.70E-01	7.70E-01		
	rs10942781	chr5	75,787,730	542	332	45	596	300	34	0.828	2.10E-02	1.68E+00		
	rs10474476	chr5	75,789,080	47	332	543	34	302	596	1.21	1.80E-02	1.74E+00		
	rs11739486	chr5	75,789,455	455	390	77	455	391	86	1.03	6.70E-01	1.74E-01		
	rs10045155	chr5	75,793,312	51	319	552	56	346	529	0.907	2.20E-01	6.58E-01		
	rs4529172	chr5	75,794,828	541	333	48	595	302	35	0.822	1.60E-02	1.80E+00		
	rs4452539	chr5	75,795,960	279	460	183	252	493	186	1.07	3.30E-01	4.81E-01		
	rs6859984	chr5	75,805,324	516	344	61	499	370	63	1.07	3.80E-01	4.20E-01		
	rs10070864	chr5	75,810,816	843	78	1	857	68	7	1.01	9.90E-01	4.36E-03		
	rs2068434	chr5	75,815,227	144	439	339	137	476	319	0.967	6.40E-01	1.94E-01		
	rs10942782	chr5	75,817,046	249	438	235	241	469	222	0.99	9.00E-01	4.58E-02		
	rs7711417	chr5	75,822,160	170	473	279	203	442	287	0.945	4.10E-01	3.87E-01		
	rs7727095	chr5	75,831,163	235	463	224	222	462	248	1.08	2.40E-01	6.20E-01		
	rs7710225	chr5	75,837,093	287	472	163	279	457	196	1.1	1.70E-01	7.70E-01		
	rs10054825	chr5	75,837,339	10	185	727	13	187	732	0.969	8.10E-01	9.15E-02		
	rs6453227	chr5	75,840,861	618	280	22	615	290	24	1.04	6.70E-01	1.74E-01		
	rs10079855	chr5	75,844,964	14	219	689	20	230	682	0.913	3.60E-01	4.44E-01		
	rs4704320	chr5	75,844,989	73	353	495	89	383	460	0.863	4.70E-02	1.33E+00		
	rs4438855	chr5	75,848,437	11	151	760	4	149	779	1.13	3.30E-01	4.81E-01		

	rs4704322	chr5	75,858,230	17	264	641	31	276	625	0.87	1.20E-01	9.21E-01		
	rs6872396	chr5	75,859,721	124	451	347	158	436	338	0.903	1.40E-01	8.54E-01		
	rs6886903	chr5	75,864,373	29	279	614	29	271	632	1.04	6.50E-01	1.87E-01		
	rs9293688	chr5	75,874,921	83	388	450	82	374	476	1.06	4.40E-01	3.57E-01		
	rs4703712	chr5	75,875,106	664	237	21	684	235	13	0.913	3.50E-01	4.56E-01		
	rs4704330	chr5	75,876,943	270	464	188	308	446	178	0.903	1.30E-01	8.86E-01		
	rs6875519	chr5	75,878,333	69	380	473	71	360	501	1.06	4.50E-01	3.47E-01		
	rs879017	chr5	75,878,727	871	51	0	884	48	0	0.929	8.00E-01	9.69E-02		
	rs10942784	chr5	75,889,806	178	465	267	173	448	296	1.08	2.80E-01	5.53E-01		
	rs10072548	chr5	75,890,974	66	388	468	72	366	494	1.04	5.90E-01	2.29E-01		
	rs6453232	chr5	75,896,451	260	462	199	299	447	186	0.895	9.90E-02	1.00E+00		
	rs4704336	chr5	75,899,375	255	460	206	292	450	190	0.893	9.20E-02	1.04E+00		
	rs12523647	chr5	75,899,743	0	53	869	0	52	880	1.03	9.60E-01	1.77E-02		
	rs3756514	chr5	75,907,035	0	53	869	0	51	881	1.05	8.80E-01	5.55E-02		
	rs16873483	chr5	75,907,106	659	243	20	694	222	16	0.87	1.50E-01	8.24E-01		
	rs3797373	chr5	75,908,204	547	336	37	569	329	30	0.925	3.50E-01	4.56E-01		
	rs17748322	chr5	75,911,385	618	272	32	651	254	26	0.883	1.60E-01	7.96E-01		
	rs905164	chr5	75,915,309	255	452	214	290	446	193	0.886	7.30E-02	1.14E+00		
	rs3822528	chr5	75,920,211	279	459	183	313	456	162	0.889	8.10E-02	1.09E+00		
	rs1501788	chr5	75,925,776	69	399	454	67	394	471	1.04	6.20E-01	2.08E-01		
	rs1501787	chr5	75,925,995	51	344	527	36	359	536	1.06	4.80E-01	3.19E-01		
	rs9293690	chr5	75,926,759	494	371	57	507	374	51	0.961	6.20E-01	2.08E-01		
	rs11948805	chr5	75,930,894	184	459	278	161	468	302	1.11	1.40E-01	8.54E-01		
	rs1393098	chr5	75,938,289	19	245	652	18	218	685	1.14	1.70E-01	7.70E-01		
	rs3797390	chr5	75,942,821	543	330	49	579	311	42	0.89	1.50E-01	8.24E-01		
	rs2069693	chr5	75,947,471	195	476	251	230	483	217	0.861	2.50E-02	1.60E+00		
	rs961536	chr5	75,948,599	210	486	226	183	484	264	1.15	3.70E-02	1.43E+00		
	rs2069685	chr5	75,949,061	314	449	151	343	466	117	0.871	4.50E-02	1.35E+00		
	rs2069683	chr5	75,949,540	909	13	0	925	7	0	0.531	2.50E-01	6.02E-01		
	rs2069682	chr5	75,950,485	1	51	870	2	46	884	1.07	8.00E-01	9.69E-02		
	rs2069664	chr5	75,952,190	184	468	270	213	485	233	0.866	3.10E-02	1.51E+00		
	rs2069662	chr5	75,952,359	3	109	807	1	109	819	1.05	7.70E-01	1.14E-01		
	rs2069698	chr5	75,952,643	1	59	862	1	73	858	0.816	2.80E-01	5.53E-01		
	rs2069656	chr5	75,953,389	54	303	565	45	329	558	0.989	9.20E-01	3.62E-02		
	rs2069655	chr5	75,953,476	14	241	667	15	236	681	1.03	8.20E-01	8.62E-02		
	rs2069641	chr5	75,955,505	423	392	84	414	402	102	1.09	2.40E-01	6.20E-01		
	rs6881371	chr5	75,958,425	488	361	73	458	387	87	1.14	8.70E-02	1.06E+00		
	rs2455223	chr5	75,959,368	338	433	151	330	434	168	1.06	3.90E-01	4.09E-01		
	rs2431359	chr5	75,960,769	840	79	3	854	75	3	0.94	7.60E-01	1.19E-01		
	rs17567779	chr5	75,961,672	1	94	827	5	87	838	0.998	9.50E-01	2.23E-02		
	rs463815	chr5	75,961,882	3	80	839	3	74	855	1.09	6.40E-01	1.94E-01		
	rs3797410	chr5	75,963,391	51	324	547	72	351	509	0.831	1.70E-02	1.77E+00		
	rs7728450	chr5	75,964,208	674	223	25	710	202	20	0.859	1.20E-01	9.21E-01		
	rs6867296	chr5	75,968,138	420	392	110	395	407	130	1.12	1.00E-01	1.00E+00		
	rs2516272	chr5	75,969,337	3	80	839	3	74	855	1.09	6.40E-01	1.94E-01		
	rs10474483	chr5	75,970,541	551	319	52	606	282	44	0.832	2.40E-02	1.62E+00		
	rs2455232	chr5	75,970,844	180	443	299	177	461	294	0.993	9.40E-01	2.69E-02		
	rs3797418	chr5	75,971,170	98	386	438	93	397	442	1.01	8.80E-01	5.55E-02		
	rs17748939	chr5	75,973,939	2	51	869	1	54	877	0.993	9.50E-01	2.23E-02		
	rs2455236	chr5	75,977,734	110	393	419	134	410	388	0.871	4.90E-02	1.31E+00		
	rs458059	chr5	75,978,692	253	426	208	232	449	209	1.05	4.80E-01	3.19E-01		
	rs2059222	chr5	75,978,912	72	360	488	63	323	543	1.18	2.70E-02	1.57E+00		
	rs615951	chr5	75,980,539	155	442	325	156	438	338	1.02	7.60E-01	1.19E-01		
	rs12697857	chr5	75,983,374	56	327	538	78	355	499	0.828	1.40E-02	1.85E+00		
	rs2431363	chr5	75,984,406	162	437	323	178	449	305	0.924	2.50E-01	6.02E-01		
	rs2431361	chr5	75,984,799	324	436	162	305	450	177	1.08	2.50E-01	6.02E-01		
	rs3815774	chr5	75,985,978	769	141	7	797	124	6	0.858	2.30E-01	6.38E-01		
	rs2455217	chr5	75,988,521	399	393	97	385	387	124	1.11	1.40E-01	8.54E-01		
	rs17681722	chr5	75,989,785	3	34	885	0	41	891	0.986	9.60E-01	1.77E-02		
	rs2287930	chr5	75,989,852	256	453	213	235	460	237	1.1	1.50E-01	8.24E-01		
	rs3797437	chr5	75,991,706	115	396	406	110	406	414	1.02	7.90E-01	1.02E-01		
	rs1038920	chr5	75,993,380	84	398	440	114	405	413	0.861	3.70E-02	1.43E+00		
	rs7734540	chr5	75,995,877	193	454	275	169	440	323	1.17	2.10E-02	1.68E+00		
	rs253093	chr5	75,996,621	0	9	913	0	8	924	1.14	9.80E-01	8.77E-03		
	rs4704350	chr5	75,998,142	11	167	744	8	171	753	1.02	8.70E-01	6.05E-02		
	rs253096	chr5	75,998,183	549	329	43	589	299	44	0.901	2.10E-01	6.78E-01		
	rs17568154	chr5	75,998,966	628	267	27	679	229	24	0.828	3.90E-02	1.41E+00		
	rs3764935	chr5	76,000,458	86	424	412	95	380	457	1.08	2.70E-01	5.69E-01		
	rs7734335	chr5	76,002,988	332	452	137	303	453	175	1.17	2.40E-02	1.62E+00		
	rs17652917	chr5	76,003,855	93	399	430	119	425	388	0.842	1.50E-02	1.82E+00		

	rs4704352	chr5	76,004,274	534	336	52	572	311	49	0.897	1.80E-01	7.45E-01		
	rs2287933	chr5	76,005,975	785	134	3	795	129	7	1.01	9.70E-01	1.32E-02		
	rs2303162	chr5	76,008,707	30	303	589	40	306	586	0.939	4.60E-01	3.37E-01		
	rs17568237	chr5	76,009,201	5	100	817	5	87	840	1.16	3.50E-01	4.56E-01		
	rs11741978	chr5	76,009,875	19	231	672	14	188	730	1.3	7.80E-03	2.11E+00		
	rs10805907	chr5	76,018,426	82	376	464	58	375	499	1.16	5.00E-02	1.30E+00		
	rs11951337	chr5	76,021,722	34	254	634	16	248	668	1.2	4.90E-02	1.31E+00		
	rs7708326	chr5	76,022,337	558	310	50	608	284	32	0.807	9.70E-03	2.01E+00		
	rs6884442	chr5	76,024,190	560	328	33	545	340	47	1.12	1.80E-01	7.45E-01		
	rs4704356	chr5	76,029,331	106	429	386	116	453	363	0.919	2.30E-01	6.38E-01		
	rs468648	chr5	76,032,621	781	129	12	782	138	12	1.05	7.10E-01	1.49E-01		
	rs10063791	chr5	76,032,969	386	430	106	363	452	116	1.09	2.40E-01	6.20E-01		
	rs7723344	chr5	76,039,770	92	445	385	103	441	388	0.974	7.30E-01	1.37E-01		
	rs153317	chr5	76,040,002	316	457	149	311	462	159	1.04	6.00E-01	2.22E-01		
	rs7728560	chr5	76,040,689	24	200	698	16	174	742	1.25	2.90E-02	1.54E+00		
	rs27951	chr5	76,041,077	73	362	487	92	391	449	0.852	3.00E-02	1.52E+00		
	rs2227744	chr5	76,046,105	212	435	275	212	448	272	0.992	9.30E-01	3.15E-02		
	rs32934	chr5	76,046,462	11	102	809	11	110	811	0.946	7.20E-01	1.43E-01		
	rs27593	chr5	76,048,669	511	336	74	546	341	45	0.844	2.90E-02	1.54E+00		
	rs37244	chr5	76,050,867	508	340	74	545	342	45	0.838	2.30E-02	1.64E+00		
	rs37248	chr5	76,052,702	458	372	92	478	380	74	0.916	2.40E-01	6.20E-01		
	rs37249	chr5	76,052,731	296	441	185	292	456	184	1.01	9.20E-01	3.62E-02		
	rs37250	chr5	76,055,072	163	437	322	163	457	312	0.974	7.20E-01	1.43E-01		
	rs153311	chr5	76,063,579	68	320	534	49	332	551	1.1	2.50E-01	6.02E-01		
	rs2227800	chr5	76,065,128	914	8	0	924	8	0	0.989	8.20E-01	8.62E-02		
	rs250736	chr5	76,070,111	40	249	633	37	244	651	1.06	5.60E-01	2.52E-01		
	rs11954573	chr5	76,070,823	67	352	500	64	373	495	0.978	7.90E-01	1.02E-01		
	rs250744	chr5	76,077,666	143	432	347	142	452	338	0.977	7.60E-01	1.19E-01		
	rs168752	chr5	76,082,411	65	368	489	76	376	480	0.936	3.90E-01	4.09E-01		
	rs250723	chr5	76,091,810	209	442	271	193	452	287	1.07	3.20E-01	4.95E-01		
	rs2047076	chr5	76,094,265	25	258	613	34	286	591	0.861	8.90E-02	1.05E+00		
	rs250724	chr5	76,097,012	225	448	249	211	464	257	1.05	5.00E-01	3.01E-01		
	rs250760	chr5	76,103,380	136	427	356	145	440	343	0.947	4.40E-01	3.57E-01		
	rs250763	chr5	76,122,353	46	278	598	35	291	606	1.05	6.20E-01	2.08E-01		
	rs463466	chr5	76,127,981	378	406	136	375	436	120	0.977	7.60E-01	1.19E-01		
	rs615986	chr5	76,128,963	135	408	379	119	437	374	1.02	7.90E-01	1.02E-01		
	rs636150	chr5	76,129,070	44	293	584	39	274	618	1.12	1.90E-01	7.21E-01		
	rs458591	chr5	76,129,130	173	441	308	159	443	329	1.08	2.80E-01	5.53E-01		
	rs1030484	chr5	76,135,668	154	449	318	166	457	307	0.947	4.30E-01	3.67E-01		
	rs13177258	chr5	76,136,292	322	451	147	313	449	166	1.07	3.40E-01	4.69E-01		
	rs6453251	chr5	76,142,908	225	472	225	212	456	264	1.12	9.60E-02	1.02E+00		
	rs639342	chr5	76,148,625	24	252	646	26	218	688	1.15	1.40E-01	8.54E-01		
	rs2242993	chr5	76,151,277	74	404	444	110	397	425	0.863	4.10E-02	1.39E+00		
	rs4704366	chr5	76,172,978	637	257	28	634	282	16	0.991	9.50E-01	2.23E-02		
	rs6883291	chr5	76,176,093	476	371	75	461	390	81	1.07	3.80E-01	4.20E-01		
	rs7724915	chr5	76,177,435	806	109	7	833	95	4	0.818	1.70E-01	7.70E-01		
	rs2460498	chr5	76,177,535	44	318	555	42	322	564	1.02	8.80E-01	5.55E-02		
	rs7729082	chr5	76,177,776	561	327	34	538	341	53	1.16	6.90E-02	1.16E+00		
	rs2460506	chr5	76,182,990	127	432	363	158	460	314	0.831	6.40E-03	2.19E+00		
	rs2460508	chr5	76,192,137	299	447	176	255	483	194	1.15	4.10E-02	1.39E+00		
	rs2359425	chr5	76,193,792	3	68	851	2	61	869	1.16	4.50E-01	3.47E-01		
	rs7712142	chr5	76,196,125	38	289	591	26	281	618	1.13	1.60E-01	7.96E-01		
	rs6453259	chr5	76,201,774	154	463	305	152	452	328	1.05	4.60E-01	3.37E-01		
	rs10462548	chr5	76,205,711	120	419	382	142	424	365	0.908	1.70E-01	7.70E-01		
	rs13188087	chr5	76,209,478	801	118	3	816	112	4	0.954	7.70E-01	1.14E-01		
	rs1320306	chr5	76,210,275	93	394	434	125	397	410	0.864	4.00E-02	1.40E+00		
	rs12519617	chr5	76,211,213	91	397	434	124	398	410	0.863	3.80E-02	1.42E+00		
	rs7722773	chr5	76,223,064	408	407	100	390	395	144	1.17	2.40E-02	1.62E+00		
	rs17749734	chr5	76,223,533	0	2	919	0	1	928	2.02	6.20E-01	2.08E-01		
	rs10942800	chr5	76,255,728	96	423	403	140	407	385	0.857	2.80E-02	1.55E+00		
	rs987951	chr5	76,256,954	73	355	494	76	415	441	0.853	3.20E-02	1.49E+00		
	rs8180518	chr5	76,258,503	365	445	112	369	435	128	1.03	6.40E-01	1.94E-01		
	rs2434651	chr5	76,259,121	70	374	478	83	389	460	0.912	2.10E-01	6.78E-01		
	rs1110413	chr5	76,259,612	21	233	668	21	253	658	0.932	4.70E-01	3.28E-01		
	rs1715762	chr5	76,260,909	603	288	31	588	314	30	1.07	4.30E-01	3.67E-01		
	rs1613519	chr5	76,261,811	213	430	279	186	466	280	1.06	3.90E-01	4.09E-01		
	rs11747190	chr5	76,266,949	10	180	732	6	197	729	0.963	7.60E-01	1.19E-01		
	rs10942804	chr5	76,267,803	781	137	4	787	142	3	1.01	9.80E-01	8.77E-03		
	rs1700687	chr5	76,268,760	256	443	222	235	457	234	1.07	2.90E-01	5.38E-01		
	rs1700686	chr5	76,268,926	51	309	556	52	345	531	0.906	2.20E-01	6.58E-01		

	rs2055627	chr5	76,270,400	10	171	737	10	196	724	0.884	2.60E-01	5.85E-01		
	rs1700680	chr5	76,274,234	101	404	416	124	422	386	0.874	5.50E-02	1.26E+00		
	rs3792738	chr5	76,283,540	5	121	792	3	102	823	1.24	1.20E-01	9.21E-01		
	rs32897	chr5	76,286,728	667	235	20	638	262	31	1.2	4.40E-02	1.36E+00		
	rs7728378	chr5	76,295,106	379	415	128	339	457	136	1.12	9.40E-02	1.03E+00		
	rs10062367	chr5	76,300,110	34	260	628	41	299	592	0.842	4.40E-02	1.36E+00		
	rs1875999	chr5	76,300,738	430	391	99	413	413	105	1.07	3.70E-01	4.32E-01		
	rs10514082	chr5	76,302,203	689	210	23	661	246	25	1.17	9.70E-02	1.01E+00		
	rs10474485	chr5	76,306,609	31	269	616	21	283	621	1.03	7.20E-01	1.43E-01		
	rs7704995	chr5	76,310,293	430	396	96	420	413	99	1.04	5.90E-01	2.29E-01		
	rs1651094	chr5	76,328,399	525	339	57	528	341	60	1.01	9.10E-01	4.10E-02		
	rs10066702	chr5	76,332,166	165	459	298	164	453	315	1.04	6.10E-01	2.15E-01		
	rs173545	chr5	76,332,563	42	268	612	20	313	599	1.01	9.40E-01	2.69E-02		
	rs247746	chr5	76,334,528	113	426	383	110	442	380	0.993	9.50E-01	2.23E-02		
	rs6898403	chr5	76,339,154	369	438	115	367	449	116	1.01	8.70E-01	6.05E-02		
	rs1035887	chr5	76,339,455	1	62	859	4	67	860	0.857	4.20E-01	3.77E-01		
	rs10473985	chr5	76,340,812	8	131	783	10	144	778	0.898	4.00E-01	3.98E-01		
	rs32902	chr5	76,344,668	282	441	197	255	480	192	1.05	4.80E-01	3.19E-01		
	rs12653511	chr5	76,346,536	363	441	113	384	429	114	0.96	5.80E-01	2.37E-01		
	rs13178007	chr5	76,347,247	337	432	153	315	468	149	1.05	5.30E-01	2.76E-01		
	rs6865782	chr5	76,347,874	551	308	62	531	351	50	1.04	6.20E-01	2.08E-01		
	rs17750118	chr5	76,348,021	433	396	93	471	384	77	0.88	7.90E-02	1.10E+00		
	rs247748	chr5	76,349,662	182	407	333	167	495	270	0.897	1.10E-01	9.59E-01		
	rs4704382	chr5	76,353,962	0	2	920	0	1	931	2.02	6.20E-01	2.08E-01		
	rs247753	chr5	76,355,650	49	292	581	43	317	572	0.972	7.60E-01	1.19E-01		
	rs3762982	chr5	76,359,244	444	378	100	465	390	77	0.903	1.60E-01	7.96E-01		
	rs995743	chr5	76,378,585	379	412	131	348	453	131	1.08	2.70E-01	5.69E-01		
	rs10072933	chr5	76,383,219	600	284	38	574	324	33	1.09	3.00E-01	5.23E-01		
	rs2545	chr5	76,396,572	102	395	425	112	424	394	0.9	1.40E-01	8.54E-01		
	rs1048834	chr5	76,405,093	729	180	13	750	176	6	0.892	3.10E-01	5.09E-01		
	rs1001960	chr5	76,406,875	12	190	720	14	211	707	0.893	2.80E-01	5.53E-01		
	rs2914143	chr5	76,415,756	38	330	554	40	336	556	0.983	8.60E-01	6.55E-02		
	rs10942811	chr5	76,424,037	71	343	508	77	368	487	0.917	2.60E-01	5.85E-01		
	rs6882793	chr5	76,434,547	362	410	150	315	443	174	1.18	1.60E-02	1.80E+00		
	rs13358038	chr5	76,439,807	80	347	495	92	374	466	0.888	1.10E-01	9.59E-01		
	rs3857395	chr5	76,442,207	361	414	147	316	441	175	1.18	1.30E-02	1.89E+00		
	rs4703727	chr5	76,443,333	493	347	79	465	372	94	1.14	8.70E-02	1.06E+00		
	rs12188833	chr5	76,443,799	7	184	724	13	192	712	0.899	3.30E-01	4.81E-01		
	rs7702095	chr5	76,450,368	78	351	493	91	386	455	0.865	5.00E-02	1.30E+00		
	rs6453278	chr5	76,452,798	448	378	96	410	410	112	1.15	4.80E-02	1.32E+00		
	rs12519589	chr5	76,453,974	13	209	700	11	216	705	0.998	9.80E-01	8.77E-03		
	rs10070420	chr5	76,454,850	7	146	766	6	148	778	1.02	9.40E-01	2.69E-02		
	rs4128741	chr5	76,459,723	136	413	373	124	434	374	1.02	7.50E-01	1.25E-01		
	rs7708285	chr5	76,461,623	438	376	91	443	385	92	1	9.80E-01	8.77E-03		
	rs12521058	chr5	76,462,743	139	412	370	140	440	352	0.952	4.90E-01	3.10E-01		
	rs6880285	chr5	76,464,235	716	197	9	750	171	11	0.875	2.20E-01	6.58E-01		
	rs10052560	chr5	76,466,058	139	412	371	140	443	349	0.944	4.10E-01	3.87E-01		
	rs7732628	chr5	76,471,102	292	445	185	303	444	183	0.974	7.20E-01	1.43E-01		
	rs6885904	chr5	76,475,006	289	446	187	290	453	189	1	9.70E-01	1.32E-02		
	rs9293707	chr5	76,475,423	171	424	327	162	450	319	0.999	9.90E-01	4.36E-03		
	rs3924674	chr5	76,476,752	3	99	806	3	114	800	0.877	3.70E-01	4.32E-01		
	rs6453285	chr5	76,481,305	8	182	731	7	174	750	1.07	5.50E-01	2.60E-01		
	rs12189028	chr5	76,482,604	6	134	782	3	125	804	1.14	3.30E-01	4.81E-01		
	rs7708818	chr5	76,484,040	1	100	821	3	111	818	0.874	3.70E-01	4.32E-01		
	rs6864250	chr5	76,485,946	183	433	306	160	460	312	1.06	3.80E-01	4.20E-01		
	rs9293710	chr5	76,486,642	180	414	327	166	443	320	1.01	8.80E-01	5.55E-02		
	rs6866080	chr5	76,487,276	139	415	367	122	415	395	1.1	1.60E-01	7.96E-01		
	rs6862414	chr5	76,490,315	471	368	81	490	366	76	0.951	5.20E-01	2.84E-01		
	rs4426900	chr5	76,496,556	118	425	379	96	465	371	1.03	7.30E-01	1.37E-01		
	rs7721952	chr5	76,499,316	6	124	792	1	144	787	0.937	6.40E-01	1.94E-01		
	rs1479561	chr5	76,503,740	22	226	674	22	241	669	0.95	6.10E-01	2.15E-01		
	rs2972341	chr5	76,504,599	209	461	252	208	455	269	1.04	5.90E-01	2.29E-01		
	rs16874057	chr5	76,504,862	3	84	835	3	84	845	1.01	1.00E+00	0.00E+00		
	rs963913	chr5	76,506,179	180	443	299	175	476	281	0.969	6.60E-01	1.80E-01		
	rs6453292	chr5	76,516,517	292	438	185	284	461	183	1.02	8.30E-01	8.09E-02		
	rs1479558	chr5	76,518,410	605	271	39	605	297	28	0.994	9.80E-01	8.77E-03		
	rs1119208	chr5	76,524,369	117	408	397	108	442	382	0.979	7.80E-01	1.08E-01		
	rs10462550	chr5	76,531,908	9	163	750	9	157	765	1.05	7.10E-01	1.49E-01		
	rs974280	chr5	76,535,005	354	433	135	344	457	131	1.02	8.00E-01	9.69E-02		
	rs9687206	chr5	76,551,580	307	430	185	302	473	157	0.954	4.90E-01	3.10E-01		

	rs12520862	chr5	76,556,268	13	205	704	18	205	709	0.964	7.50E-01	1.25E-01		
	rs12516568	chr5	76,557,177	13	205	703	18	204	708	0.968	7.80E-01	1.08E-01		
	rs1382879	chr5	76,557,624	339	444	139	351	452	129	0.956	5.30E-01	2.76E-01		
	rs2046045	chr5	76,571,567	337	442	143	349	454	129	0.947	4.40E-01	3.57E-01		
	rs12515498	chr5	76,575,728	517	343	58	544	327	59	0.943	4.70E-01	3.28E-01		
	rs1351283	chr5	76,580,698	376	424	122	399	424	109	0.925	2.70E-01	5.69E-01		
	rs989758	chr5	76,580,716	122	421	378	109	424	399	1.08	3.10E-01	5.09E-01		
	rs1096752	chr5	76,585,314	200	462	260	206	487	239	0.942	3.80E-01	4.20E-01		
	rs4703730	chr5	76,585,444	225	482	214	231	476	220	1	9.80E-01	8.77E-03		
	rs832785	chr5	76,586,085	353	439	129	327	472	132	1.07	3.10E-01	5.09E-01		
	rs4704400	chr5	76,586,766	209	486	226	220	479	233	0.991	9.20E-01	3.62E-02		
	rs7702192	chr5	76,590,563	181	464	277	184	459	289	1.02	8.20E-01	8.62E-02		
	rs2859576	chr5	76,592,073	723	189	10	758	166	8	0.846	1.30E-01	8.86E-01		
	rs7714529	chr5	76,598,001	72	416	434	93	387	452	0.983	8.30E-01	8.09E-02		
	rs16874127	chr5	76,599,527	813	105	4	820	106	6	1.04	8.50E-01	7.06E-02		
	rs832790	chr5	76,604,048	152	435	334	138	424	369	1.11	1.20E-01	9.21E-01		
	rs6885813	chr5	76,605,367	65	328	518	42	350	531	1.09	2.70E-01	5.69E-01		
	rs13361710	chr5	76,606,098	58	326	535	41	353	537	1.04	6.50E-01	1.87E-01		
	rs251421	chr5	76,608,726	517	345	58	514	358	60	1.03	7.10E-01	1.49E-01		
	rs251416	chr5	76,614,391	44	337	541	54	335	543	0.961	6.30E-01	2.01E-01		
	rs251411	chr5	76,623,912	43	335	544	54	331	547	0.96	6.30E-01	2.01E-01		
	rs12514694	chr5	76,627,454	9	203	710	11	196	725	1.03	8.20E-01	8.62E-02		
	rs844274	chr5	76,628,500	43	335	544	54	331	547	0.96	6.30E-01	2.01E-01		
	rs10066802	chr5	76,633,217	119	403	400	99	427	406	1.06	4.50E-01	3.47E-01		
	rs10045589	chr5	76,649,815	464	395	63	456	406	70	1.05	5.10E-01	2.92E-01		
	rs10474494	chr5	76,650,146	79	415	428	87	419	426	0.966	6.50E-01	1.87E-01		
	rs6863296	chr5	76,652,494	805	114	3	814	114	4	1.01	9.80E-01	8.77E-03		
	rs1531615	chr5	76,654,613	179	462	281	170	468	294	1.05	5.10E-01	2.92E-01		
	rs6873924	chr5	76,656,338	0	65	857	0	72	860	0.909	6.50E-01	1.87E-01		
	rs3797462	chr5	76,657,430	61	394	467	67	405	460	0.955	5.50E-01	2.60E-01		
	rs9686502	chr5	76,659,177	242	467	213	251	474	207	0.969	6.50E-01	1.87E-01		
	rs9687030	chr5	76,663,192	306	446	170	282	491	159	1.03	6.60E-01	1.80E-01		
	rs7703993	chr5	76,669,108	615	281	26	615	292	25	1.02	8.50E-01	7.06E-02		
	rs11949597	chr5	76,680,266	2	88	832	1	89	842	1.02	9.40E-01	2.69E-02		
	rs2046046	chr5	76,680,718	439	395	83	439	399	87	1.02	8.30E-01	8.09E-02		
	rs2306344	chr5	76,681,558	75	396	451	73	415	444	0.977	7.70E-01	1.14E-01		
	rs7723920	chr5	76,687,906	279	475	168	287	462	183	1.02	8.10E-01	9.15E-02		
	rs17683162	chr5	76,689,508	142	422	358	122	442	368	1.07	3.70E-01	4.32E-01		
	rs4704416	chr5	76,698,274	493	367	62	510	368	54	0.945	4.70E-01	3.28E-01		
	rs10514096	chr5	76,700,940	48	353	521	48	370	513	0.964	6.60E-01	1.80E-01		
	rs6453304	chr5	76,706,384	20	255	647	18	258	652	1.01	9.30E-01	3.15E-02		
	rs7732963	chr5	76,707,300	0	68	854	1	67	863	0.995	9.50E-01	2.23E-02		
	rs7715119	chr5	76,711,174	18	201	703	17	209	706	0.984	9.10E-01	4.10E-02		
	rs2359874	chr5	76,713,932	330	453	139	329	445	157	1.05	5.10E-01	2.92E-01		
	rs335618	chr5	76,721,372	300	484	138	309	471	152	1.02	8.50E-01	7.06E-02		
	rs2359875	chr5	76,731,296	557	314	50	579	313	39	0.917	2.90E-01	5.38E-01		
	rs3733952	chr5	76,732,090	33	286	603	29	278	625	1.07	4.30E-01	3.67E-01		
	rs6874493	chr5	76,737,495	193	477	252	196	488	248	0.984	8.30E-01	8.09E-02		
	rs374003	chr5	76,737,959	646	258	18	651	258	23	1.03	8.00E-01	9.69E-02		
	rs7705337	chr5	76,739,091	2	58	862	1	51	880	1.19	4.10E-01	3.87E-01		
	rs3101866	chr5	76,739,130	225	465	232	205	493	234	1.05	4.90E-01	3.10E-01		
	rs2359876	chr5	76,739,394	45	345	532	56	341	534	0.96	6.30E-01	2.01E-01		
	rs11956168	chr5	76,740,007	6	135	781	3	153	776	0.929	5.80E-01	2.37E-01		
	rs10514098	chr5	76,740,837	683	220	19	698	219	15	0.948	6.10E-01	2.15E-01		
	rs9293713	chr5	76,741,011	5	119	796	4	113	815	1.09	5.70E-01	2.44E-01		
	rs335614	chr5	76,744,743	132	433	357	141	424	367	0.997	9.90E-01	4.36E-03		
	rs2279095	chr5	76,745,182	9	146	767	5	148	779	1.05	6.90E-01	1.61E-01		
	rs12521494	chr5	76,747,918	562	321	39	563	327	42	1.02	7.90E-01	1.02E-01		
	rs890717	chr5	76,748,331	692	219	10	690	225	16	1.07	4.90E-01	3.10E-01		
	rs2242159	chr5	76,750,136	176	452	294	159	473	300	1.05	4.90E-01	3.10E-01		
	rs2242160	chr5	76,750,887	385	431	106	405	413	114	0.979	7.80E-01	1.08E-01		
	rs335644	chr5	76,752,669	183	480	259	201	464	267	0.977	7.50E-01	1.25E-01		
	rs335643	chr5	76,753,826	534	338	50	538	339	55	1.02	8.40E-01	7.57E-02		
	rs335640	chr5	76,756,000	77	397	448	89	403	439	0.94	4.00E-01	3.98E-01		
	rs2359877	chr5	76,756,293	2	128	792	6	136	790	0.894	4.00E-01	3.98E-01		
	rs90682	chr5	76,756,845	357	441	124	387	420	125	0.941	3.90E-01	4.09E-01		
	rs3816609	chr5	76,757,694	114	429	379	112	412	408	1.07	3.50E-01	4.56E-01		
	rs335636	chr5	76,760,355	203	472	247	222	445	265	0.997	9.90E-01	4.36E-03		
	rs7447456	chr5	76,761,958	624	248	34	632	268	18	0.939	5.10E-01	2.92E-01		
	rs3149	chr5	76,764,192	578	296	48	597	305	30	0.902	2.20E-01	6.58E-01		

	rs335630	chr5	76,765,134	443	386	80	428	399	85	1.06	4.70E-01	3.28E-01		
	rs4704421	chr5	76,765,470	3	82	837	1	87	844	0.999	9.40E-01	2.69E-02		
	rs335624	chr5	76,766,692	233	473	216	251	449	232	0.996	9.80E-01	8.77E-03		
	rs919224	chr5	76,769,135	136	453	333	144	420	368	1.06	4.20E-01	3.77E-01		
	rs33204	chr5	76,769,840	188	477	256	206	441	284	1.02	7.90E-01	1.02E-01		
	rs2242158	chr5	76,770,300	895	27	0	908	23	1	0.915	8.60E-01	6.55E-02		
	rs441102	chr5	76,774,726	16	242	663	28	240	664	0.926	4.20E-01	3.77E-01		
	rs463592	chr5	76,790,456	800	117	5	794	135	3	1.11	4.60E-01	3.37E-01		
	rs17751165	chr5	76,792,067	17	207	688	15	203	698	1.04	6.90E-01	1.61E-01		
	rs690280	chr5	76,794,989	166	463	290	185	436	309	0.997	9.90E-01	4.36E-03		
	rs3797644	chr5	76,795,372	378	429	114	407	412	113	0.939	3.80E-01	4.20E-01		
	rs251470	chr5	76,814,241	179	463	280	195	438	299	1	9.80E-01	8.77E-03		
	rs163035	chr5	76,815,798	203	472	247	221	446	265	0.999	9.90E-01	4.36E-03		
	rs163030	chr5	76,817,227	202	473	247	221	446	265	0.997	9.90E-01	4.36E-03		
	rs33198	chr5	76,825,021	240	464	218	250	445	237	1.02	7.90E-01	1.02E-01		
	rs2359879	chr5	76,841,025	5	162	755	8	157	767	1.01	9.90E-01	4.36E-03		
	rs10474516	chr5	76,852,199	405	410	107	409	406	117	1.02	7.80E-01	1.08E-01		
	rs1460042	chr5	76,857,474	101	402	418	105	398	429	1.01	9.40E-01	2.69E-02		
	rs6872745	chr5	76,868,712	146	431	345	162	424	346	0.962	5.90E-01	2.29E-01		
	rs3846672	chr5	76,871,060	184	453	283	192	445	294	1	9.80E-01	8.77E-03		
	rs3846673	chr5	76,871,129	232	457	229	230	448	252	1.06	4.30E-01	3.67E-01		
	rs7729096	chr5	76,871,684	1	14	907	0	30	902	0.535	5.80E-02	1.24E+00		
	rs10070168	chr5	76,871,811	405	407	110	420	397	115	0.984	8.40E-01	7.57E-02		
	rs7729808	chr5	76,885,287	659	240	23	626	292	13	1.12	2.10E-01	6.78E-01		
	rs10052921	chr5	76,893,626	603	283	36	576	331	25	1.08	3.90E-01	4.09E-01		
	rs10063453	chr5	76,898,322	775	140	6	783	142	7	1.02	9.40E-01	2.69E-02		
	rs12655063	chr5	76,901,725	5	139	778	4	156	772	0.911	4.70E-01	3.28E-01		
	rs12697868	chr5	76,904,514	58	325	539	42	346	544	1.05	5.70E-01	2.44E-01		
	rs6453323	chr5	76,904,706	377	409	136	358	459	115	1	9.90E-01	4.36E-03		
	rs6453330	chr5	76,907,101	517	341	61	517	363	50	0.984	8.60E-01	6.55E-02		
	rs4704434	chr5	76,926,324	208	461	252	188	502	242	1.02	7.80E-01	1.08E-01		
	rs7703420	chr5	76,926,777	15	195	712	8	184	740	1.16	1.80E-01	7.45E-01		
	rs4532348	chr5	76,927,185	266	454	192	282	462	172	0.924	2.50E-01	6.02E-01		
	rs10474519	chr5	76,929,703	12	182	724	7	170	752	1.15	2.10E-01	6.78E-01		
	rs4374746	chr5	76,931,773	16	224	682	13	236	683	0.986	9.20E-01	3.62E-02		
	rs3933483	chr5	76,939,448	706	202	13	707	206	19	1.07	5.50E-01	2.60E-01		
	rs6868555	chr5	76,955,681	238	455	229	239	481	212	0.962	5.80E-01	2.37E-01		
	rs2241367	chr5	76,967,981	374	414	134	366	437	129	1.01	8.80E-01	5.55E-02		
	rs11746588	chr5	76,969,262	36	291	595	34	314	584	0.951	5.70E-01	2.44E-01		
	rs4266383	chr5	76,985,514	103	416	403	95	431	406	1.02	8.20E-01	8.62E-02		
	rs6453340	chr5	76,998,030	2	131	789	2	128	802	1.04	8.30E-01	8.09E-02		
	rs4579228	chr5	76,999,430	788	132	2	804	127	1	0.934	6.40E-01	1.94E-01		
	rs1363443	chr5	77,003,202	803	118	1	827	104	1	0.866	3.30E-01	4.81E-01		
	rs456910	chr5	77,007,645	211	444	253	187	484	248	1.04	5.60E-01	2.52E-01		
	rs441492	chr5	77,009,108	136	419	364	115	443	372	1.06	3.90E-01	4.09E-01		
	rs351871	chr5	77,011,795	308	436	173	262	488	175	1.11	1.10E-01	9.59E-01		
	rs2112549	chr5	77,012,598	800	121	1	827	104	1	0.844	2.40E-01	6.20E-01		
	rs13156814	chr5	77,015,150	27	267	627	19	265	648	1.09	3.60E-01	4.44E-01		
	rs2652217	chr5	77,016,000	155	435	332	128	444	360	1.13	8.10E-02	1.09E+00		
	rs16874482	chr5	77,017,565	29	272	621	21	264	647	1.11	2.50E-01	6.02E-01		
	rs3776921	chr5	77,023,468	2	148	770	1	125	805	1.23	1.10E-01	9.59E-01		
	rs2662352	chr5	77,039,051	334	439	149	362	445	125	0.893	1.00E-01	1.00E+00		
	rs2652210	chr5	77,044,009	175	430	317	194	477	261	0.847	1.30E-02	1.89E+00		
	rs384109	chr5	77,055,665	431	393	94	447	397	81	0.935	3.70E-01	4.32E-01		
	rs11954532	chr5	77,061,922	21	197	704	9	191	732	1.18	1.10E-01	9.59E-01		
	rs164814	chr5	77,075,851	336	437	149	364	444	124	0.891	9.30E-02	1.03E+00		
	rs352580	chr5	77,076,628	128	440	354	132	425	375	1.03	6.50E-01	1.87E-01		
	rs153660	chr5	77,114,267	2	114	804	3	90	838	1.26	1.20E-01	9.21E-01		
	rs254412	chr5	77,115,208	429	393	99	449	400	83	0.923	2.70E-01	5.69E-01		
	rs254409	chr5	77,116,026	247	447	228	263	478	191	0.893	9.00E-02	1.05E+00		
	rs1542891	chr5	77,119,407	765	154	3	792	139	1	0.861	2.40E-01	6.20E-01		
	rs6453351	chr5	77,130,159	74	370	478	76	408	448	0.91	2.00E-01	6.99E-01		
	rs353951	chr5	77,134,325	215	472	232	208	483	239	1.03	6.80E-01	1.67E-01		
	rs353958	chr5	77,149,373	351	446	125	347	456	129	1.02	7.50E-01	1.25E-01		
	rs16874730	chr5	77,152,147	2	117	801	0	83	849	1.51	5.80E-03	2.24E+00		
	rs13170774	chr5	77,153,515	180	477	263	196	470	266	0.97	6.70E-01	1.74E-01		
	rs353964	chr5	77,153,970	6	162	754	4	125	803	1.36	1.30E-02	1.89E+00		
	rs16874736	chr5	77,154,664	0	50	871	1	46	884	1.05	8.80E-01	5.55E-02		
	rs353966	chr5	77,155,123	920	2	0	932	0	0	0	2.50E-01	6.02E-01		
	rs353967	chr5	77,155,814	1	43	878	1	57	874	0.765	2.20E-01	6.58E-01		

	rs10942832	chr5	77,156,405	641	260	20	678	228	26	0.909	3.10E-01	5.09E-01		
	rs354616	chr5	77,157,996	2	95	825	6	99	827	0.896	4.80E-01	3.19E-01		
	rs6884279	chr5	77,161,966	539	325	55	578	305	46	0.876	1.00E-01	1.00E+00		
	rs10514122	chr5	77,164,410	69	365	488	72	385	475	0.947	4.80E-01	3.19E-01		
	rs354599	chr5	77,170,153	650	251	21	655	249	28	1.04	7.30E-01	1.37E-01		
	rs16874792	chr5	77,176,956	921	1	0	931	0	0	0	5.00E-01	3.01E-01		
	rs6881736	chr5	77,179,822	215	445	262	224	468	240	0.935	3.20E-01	4.95E-01		
	rs4262083	chr5	77,187,680	279	448	195	271	470	191	1.01	9.00E-01	4.58E-02		
	rs1443096	chr5	77,192,490	590	301	31	575	316	38	1.09	3.10E-01	5.09E-01		
	rs10514124	chr5	77,195,152	107	427	387	92	431	406	1.08	2.90E-01	5.38E-01		
	rs7730738	chr5	77,195,712	9	213	700	12	213	707	0.983	9.00E-01	4.58E-02		
	rs4490567	chr5	77,203,846	163	480	279	164	471	297	1.04	6.30E-01	2.01E-01		
	rs9687161	chr5	77,206,176	169	442	311	150	476	306	1.03	7.00E-01	1.55E-01		
	rs11741162	chr5	77,206,186	155	475	292	150	466	316	1.06	3.80E-01	4.20E-01		
	rs2028595	chr5	77,206,340	123	452	347	135	450	347	0.968	6.50E-01	1.87E-01		
	rs7734027	chr5	77,209,278	85	408	428	98	407	427	0.956	5.50E-01	2.60E-01		
	rs12656623	chr5	77,216,618	11	204	707	19	227	686	0.843	8.70E-02	1.06E+00		
	rs10514128	chr5	77,218,411	650	255	17	650	257	25	1.06	5.40E-01	2.68E-01		
	rs10440685	chr5	77,218,843	751	166	5	737	188	7	1.15	2.10E-01	6.78E-01		
	rs10060355	chr5	77,223,232	473	395	54	480	372	80	1.06	4.10E-01	3.87E-01		
	rs16874849	chr5	77,237,118	11	208	703	17	238	677	0.834	6.60E-02	1.18E+00		
	rs2885240	chr5	77,237,767	9	196	716	13	193	726	0.987	9.40E-01	2.69E-02		
	rs10069818	chr5	77,238,316	283	465	174	306	443	183	0.972	7.00E-01	1.55E-01		
	rs6879124	chr5	77,243,578	0	56	866	0	50	882	1.14	5.80E-01	2.37E-01		
	rs2289599	chr5	77,243,905	82	395	445	92	379	461	1.01	9.70E-01	1.32E-02		
	rs5014235	chr5	77,245,417	81	396	445	93	379	460	0.998	1.00E+00	0.00E+00		
	rs4704458	chr5	77,245,924	386	422	114	409	401	122	0.972	7.10E-01	1.49E-01		
	rs2120101	chr5	77,246,636	209	490	220	226	473	227	0.978	7.70E-01	1.14E-01		
	rs355425	chr5	77,247,426	22	275	625	29	264	639	1	9.80E-01	8.77E-03		
	rs902523	chr5	77,251,993	667	237	18	684	231	15	0.939	5.30E-01	2.76E-01		
	rs4704462	chr5	77,254,887	203	488	231	226	483	223	0.935	3.20E-01	4.95E-01		
	rs10942835	chr5	77,258,559	228	480	214	231	492	209	0.983	8.20E-01	8.62E-02		
	rs4704464	chr5	77,258,895	716	195	11	743	177	12	0.906	3.70E-01	4.32E-01		
	rs7712357	chr5	77,260,422	243	460	218	241	472	219	1.01	9.40E-01	2.69E-02		
	rs16874901	chr5	77,266,238	0	59	863	0	62	870	0.961	9.00E-01	4.58E-02		
	rs2044163	chr5	77,267,160	0	57	865	0	60	872	0.959	9.00E-01	4.58E-02		
	rs837036	chr5	77,275,676	412	402	107	392	435	105	1.05	4.80E-01	3.19E-01		
	rs7725100	chr5	77,280,552	388	413	116	380	436	116	1.03	7.00E-01	1.55E-01		
	rs4484406	chr5	77,280,768	55	371	496	53	378	501	1.01	9.60E-01	1.77E-02		
	rs10044946	chr5	77,283,634	86	387	449	76	415	441	0.995	9.70E-01	1.32E-02		
	rs4704470	chr5	77,283,862	37	348	536	41	342	549	1.01	9.40E-01	2.69E-02		
	rs12521066	chr5	77,285,180	415	399	108	406	426	99	1.01	9.40E-01	2.69E-02		
	rs7730112	chr5	77,287,544	37	349	536	40	343	549	1.01	8.90E-01	5.06E-02		
	rs1948326	chr5	77,297,718	861	60	1	871	60	0	0.957	8.80E-01	5.55E-02		
	rs355421	chr5	77,298,038	4	151	766	4	131	797	1.17	2.10E-01	6.78E-01		
	rs13154492	chr5	77,298,338	1	97	815	3	99	822	0.952	7.90E-01	1.02E-01		
	rs6864833	chr5	77,302,045	805	114	3	815	114	3	0.989	9.80E-01	8.77E-03		
	rs355419	chr5	77,302,374	434	394	94	429	404	99	1.03	6.60E-01	1.80E-01		
	rs355418	chr5	77,302,448	248	432	242	228	456	248	1.06	4.10E-01	3.87E-01		
	rs355416	chr5	77,308,682	122	408	392	114	401	417	1.07	3.20E-01	4.95E-01		
	rs355415	chr5	77,309,010	715	198	9	751	172	9	0.855	1.50E-01	8.24E-01		
	rs6884769	chr5	77,310,268	744	168	10	731	193	8	1.11	3.40E-01	4.69E-01		
	rs6874954	chr5	77,312,857	297	449	176	279	441	212	1.13	7.50E-02	1.12E+00		
	rs10064609	chr5	77,314,628	101	436	377	116	414	398	1	9.80E-01	8.77E-03		
	rs355412	chr5	77,315,891	162	458	301	172	456	302	0.978	7.60E-01	1.19E-01		
	rs837019	chr5	77,317,459	42	316	564	32	341	559	0.998	9.90E-01	4.36E-03		
	rs2029526	chr5	77,320,898	474	359	73	488	349	77	0.983	8.40E-01	7.57E-02		
	rs1873425	chr5	77,325,504	134	453	335	176	392	364	0.966	6.40E-01	1.94E-01		
	rs10078715	chr5	77,326,658	2	113	807	4	101	827	1.09	5.70E-01	2.44E-01		
	rs6887042	chr5	77,328,470	455	388	79	472	361	99	1.02	8.30E-01	8.09E-02		
	rs3776924	chr5	77,342,146	58	348	516	69	342	521	0.969	7.10E-01	1.49E-01		
	rs3822645	chr5	77,349,006	895	27	0	907	25	0	0.915	8.60E-01	6.55E-02		
	rs7718254	chr5	77,349,876	922	0	0	929	3	0	0	2.50E-01	6.02E-01		
	rs4704474	chr5	77,350,342	52	323	529	62	313	542	0.988	9.10E-01	4.10E-02		
	rs380913	chr5	77,372,332	45	322	555	56	297	578	1.02	8.10E-01	9.15E-02		
	rs252792	chr5	77,386,080	556	321	45	579	300	53	0.971	7.40E-01	1.31E-01		
	rs252783	chr5	77,388,082	45	321	556	53	298	581	1.04	6.80E-01	1.67E-01		
	rs34436	chr5	77,394,844	734	180	8	767	159	6	0.849	1.50E-01	8.24E-01		
	rs252761	chr5	77,416,479	299	445	146	328	399	166	0.981	8.00E-01	9.69E-02		
	rs252751	chr5	77,424,969	4	157	752	4	129	797	1.25	7.40E-02	1.13E+00		

	rs252749	chr5	77,425,729	54	343	525	50	334	548	1.07	4.20E-01	3.77E-01		
	rs3776928	chr5	77,434,007	0	56	865	1	54	877	1.01	9.70E-01	1.32E-02		
	rs17191796	chr5	77,444,232	710	201	11	702	214	15	1.1	3.80E-01	4.20E-01		
	rs42360	chr5	77,447,767	525	343	54	551	334	47	0.921	3.00E-01	5.23E-01		
	rs2052477	chr5	77,473,098	52	330	538	46	319	566	1.09	2.90E-01	5.38E-01		
	rs10755299	chr5	77,477,055	49	332	541	44	322	566	1.08	3.60E-01	4.44E-01		
	rs4388193	chr5	77,500,300	0	47	875	1	50	881	0.911	7.20E-01	1.43E-01		
	rs10428660	chr5	77,509,175	504	354	64	527	345	60	0.939	4.30E-01	3.67E-01		
	rs6453374	chr5	77,543,915	89	409	423	110	406	413	0.921	2.50E-01	6.02E-01		
	rs10039931	chr5	77,549,929	285	454	183	299	426	207	1.02	7.40E-01	1.31E-01		
	rs10043750	chr5	77,560,400	92	414	416	103	410	419	0.972	7.10E-01	1.49E-01		
	rs6872407	chr5	77,561,551	450	393	79	457	385	90	1.02	8.00E-01	9.69E-02		
	rs6865361	chr5	77,573,127	416	414	92	417	412	103	1.03	6.60E-01	1.80E-01		
	rs7726585	chr5	77,582,552	158	464	292	180	415	322	1.02	8.30E-01	8.09E-02		
	rs10075003	chr5	77,596,133	85	390	446	93	394	443	0.964	6.30E-01	2.01E-01		
	rs10059285	chr5	77,608,195	73	366	482	65	406	460	0.952	5.30E-01	2.76E-01		
	rs13163558	chr5	77,613,462	73	367	482	65	405	462	0.957	5.80E-01	2.37E-01		
	rs10805919	chr5	77,628,680	489	351	56	500	360	60	1.01	8.90E-01	5.06E-02		
	rs9790855	chr5	77,634,610	101	393	428	97	438	397	0.929	3.10E-01	5.09E-01		
	rs4572979	chr5	77,641,971	171	459	292	156	463	313	1.08	2.70E-01	5.69E-01		
	rs4348192	chr5	77,652,915	256	460	206	249	485	198	0.999	9.90E-01	4.36E-03		
	rs10474543	chr5	77,653,935	62	367	493	66	368	498	0.99	9.20E-01	3.62E-02		
	rs7356739	chr5	77,658,624	511	348	60	496	362	70	1.09	2.90E-01	5.38E-01		
	rs4704495	chr5	77,660,666	358	425	135	392	413	125	0.909	1.70E-01	7.70E-01		
	rs4704496	chr5	77,660,704	41	286	595	51	287	594	0.945	5.20E-01	2.84E-01		
	rs6881634	chr5	77,666,610	341	436	145	348	453	131	0.958	5.50E-01	2.60E-01		
	rs4588572	chr5	77,667,390	568	304	50	579	296	57	1.01	9.80E-01	8.77E-03		
	rs4348193	chr5	77,671,476	3	63	856	1	91	840	0.74	7.50E-02	1.12E+00		
	rs13154903	chr5	77,672,488	747	167	8	714	206	12	1.28	2.20E-02	1.66E+00		
	rs9293744	chr5	77,672,779	450	383	89	420	413	99	1.12	1.30E-01	8.86E-01		
	rs11749979	chr5	77,675,946	307	432	183	354	434	144	0.829	5.50E-03	2.26E+00		
	rs7722126	chr5	77,681,540	59	348	515	65	363	503	0.939	4.20E-01	3.77E-01		
	rs4267850	chr5	77,683,520	190	453	278	206	466	260	0.927	2.60E-01	5.85E-01		
	rs4704506	chr5	77,690,583	863	57	2	849	82	1	1.38	7.20E-02	1.14E+00		
	rs10065260	chr5	77,699,192	232	447	243	223	476	233	0.998	1.00E+00	0.00E+00		
	rs12110158	chr5	77,710,302	231	441	245	218	477	234	1	9.80E-01	8.77E-03		
	rs4360024	chr5	77,719,962	872	48	2	867	64	1	1.26	2.50E-01	6.02E-01		
	rs1346566	chr5	77,731,814	717	193	12	741	179	7	0.871	2.10E-01	6.78E-01		
	rs3822476	chr5	77,749,577	850	69	3	850	80	2	1.11	5.60E-01	2.52E-01		
	rs16875382	chr5	77,754,242	921	1	0	929	3	0	2.97	6.20E-01	2.08E-01		
	rs1968382	chr5	77,757,160	449	383	90	446	407	79	0.99	9.10E-01	4.10E-02		
	rs6860842	chr5	77,775,106	70	367	467	61	373	482	1.05	5.10E-01	2.92E-01		
	rs6873144	chr5	77,777,540	549	327	46	546	337	49	1.04	6.90E-01	1.61E-01		
	rs10063742	chr5	77,795,118	38	291	593	40	290	602	1	1.00E+00	0.00E+00		
	rs1159930	chr5	77,804,699	46	333	543	52	336	544	0.969	7.20E-01	1.43E-01		
	rs1428864	chr5	77,816,019	545	335	42	543	338	51	1.05	5.50E-01	2.60E-01		
	rs10514138	chr5	77,822,520	245	447	230	230	478	224	1.02	7.90E-01	1.02E-01		
	rs11952677	chr5	77,823,690	482	363	77	493	356	83	0.998	9.90E-01	4.36E-03		
	rs16875506	chr5	77,824,547	443	385	94	442	409	81	0.98	8.00E-01	9.69E-02		
	rs7700390	chr5	77,825,288	34	299	589	44	288	600	0.983	8.70E-01	6.05E-02		
	rs11737914	chr5	77,832,467	472	373	71	495	376	57	0.917	2.60E-01	5.85E-01		
	rs876606	chr5	77,836,839	75	366	481	64	376	492	1.05	5.40E-01	2.68E-01		
	rs876605	chr5	77,837,115	47	356	519	65	349	518	0.933	3.80E-01	4.20E-01		
	rs751659	chr5	77,837,944	11	124	787	14	150	768	0.814	8.90E-02	1.05E+00		
	rs6874671	chr5	77,840,571	12	192	718	11	180	741	1.09	4.30E-01	3.67E-01		
	rs10462558	chr5	77,842,744	878	44	0	878	52	2	1.27	2.90E-01	5.38E-01		
	rs11749358	chr5	77,845,745	470	378	74	481	389	62	0.951	5.20E-01	2.84E-01		
	rs7711188	chr5	77,847,064	131	405	386	134	423	375	0.962	5.90E-01	2.29E-01		
	rs7713690	chr5	77,850,557	370	408	144	362	423	147	1.03	6.80E-01	1.67E-01		
	rs4704514	chr5	77,855,837	74	339	509	84	367	481	0.891	1.30E-01	8.86E-01		
	rs6453401	chr5	77,865,196	268	455	199	265	443	223	1.06	3.80E-01	4.20E-01		
	rs7730720	chr5	77,871,110	266	444	211	263	432	235	1.06	3.80E-01	4.20E-01		
	rs965829	chr5	77,885,683	260	457	205	263	436	233	1.06	4.20E-01	3.77E-01		
	rs344651	chr5	77,888,754	613	280	29	676	235	21	0.778	5.20E-03	2.28E+00		
	rs13183553	chr5	77,923,494	206	456	260	239	431	262	0.934	3.20E-01	4.95E-01		
	rs344656	chr5	77,927,783	736	180	6	758	159	15	0.971	8.30E-01	8.09E-02		
	rs10060136	chr5	77,943,384	273	451	197	274	432	226	1.06	3.60E-01	4.44E-01		
	rs1372319	chr5	77,945,695	430	397	94	491	365	76	0.825	8.20E-03	2.09E+00		
	rs1441711	chr5	77,946,687	303	441	178	292	440	200	1.08	2.70E-01	5.69E-01		
	rs1035856	chr5	77,952,104	0	38	884	1	42	889	0.87	6.10E-01	2.15E-01		

	rs9293756	chr5	77,953,892	291	434	197	276	448	208	1.06	3.90E-01	4.09E-01		
	rs12651937	chr5	77,958,295	100	402	420	130	419	383	0.846	1.70E-02	1.77E+00		
	rs9293757	chr5	77,959,776	99	403	419	129	421	382	0.845	1.70E-02	1.77E+00		
	rs7722214	chr5	77,962,037	83	397	442	122	404	406	0.825	6.70E-03	2.17E+00		
	rs17391168	chr5	77,962,789	439	396	87	400	412	120	1.2	9.30E-03	2.03E+00		
	rs341903	chr5	77,968,847	529	340	53	580	308	44	0.846	3.60E-02	1.44E+00		
	rs341930	chr5	77,973,209	173	453	291	157	471	301	1.06	4.40E-01	3.57E-01		
	rs341917	chr5	77,985,321	225	449	226	217	466	211	0.984	8.40E-01	7.57E-02		
	rs184652	chr5	77,997,230	425	395	102	468	376	88	0.874	6.40E-02	1.19E+00		
	rs6868351	chr5	77,998,195	505	345	72	461	386	85	1.18	2.80E-02	1.55E+00		
	rs341908	chr5	78,000,473	510	341	71	462	386	84	1.19	1.80E-02	1.74E+00		
	rs341911	chr5	78,002,381	637	256	29	611	288	33	1.14	1.30E-01	8.86E-01		
	rs1961557	chr5	78,008,926	37	294	586	38	295	592	1	9.70E-01	1.32E-02		
	rs341900	chr5	78,012,796	510	338	66	459	381	80	1.2	1.40E-02	1.85E+00		
	rs413921	chr5	78,020,121	644	249	29	619	281	32	1.14	1.50E-01	8.24E-01		
	rs784420	chr5	78,023,280	458	374	88	486	371	75	0.91	2.10E-01	6.78E-01		
	rs340085	chr5	78,034,542	209	462	251	222	466	244	0.957	5.20E-01	2.84E-01		
	rs920581	chr5	78,035,032	738	172	12	722	202	8	1.11	3.30E-01	4.81E-01		
	rs920584	chr5	78,038,407	676	228	18	674	239	19	1.04	6.70E-01	1.74E-01		
	rs6893444	chr5	78,047,949	22	243	657	24	243	665	0.996	1.00E+00	0.00E+00		
	rs11750463	chr5	78,052,472	2	102	818	5	106	821	0.919	5.90E-01	2.29E-01		
	rs745981	chr5	78,052,616	506	357	59	520	350	62	0.983	8.50E-01	7.06E-02		
	rs539990	chr5	78,055,922	332	421	167	342	450	138	0.92	2.30E-01	6.38E-01		
	rs569523	chr5	78,060,122	85	359	478	81	400	451	0.932	3.50E-01	4.56E-01		
	rs252241	chr5	78,064,724	537	329	56	579	310	43	0.858	5.70E-02	1.24E+00		
	rs6897874	chr5	78,070,277	238	470	214	247	462	223	1	9.80E-01	8.77E-03		
	rs6878810	chr5	78,070,561	12	166	744	8	158	766	1.12	3.50E-01	4.56E-01		
	rs6866961	chr5	78,071,900	9	190	723	5	202	725	0.991	9.70E-01	1.32E-02		
	rs2453766	chr5	78,072,894	31	250	641	29	297	606	0.866	1.00E-01	1.00E+00		
	rs13164947	chr5	78,077,732	298	443	180	282	472	178	1.03	6.40E-01	1.94E-01		
	rs13164005	chr5	78,077,895	103	432	387	109	401	422	1.06	3.90E-01	4.09E-01		
	rs16875859	chr5	78,077,971	819	98	5	829	102	1	0.95	7.70E-01	1.14E-01		
	rs2173013	chr5	78,086,979	758	153	11	755	170	7	1.04	7.40E-01	1.31E-01		
	rs546261	chr5	78,087,129	758	152	11	755	170	7	1.05	7.00E-01	1.55E-01		
	rs252235	chr5	78,096,896	725	184	13	719	195	18	1.1	3.70E-01	4.32E-01		
	rs2029905	chr5	78,098,166	51	356	515	47	338	547	1.1	2.50E-01	6.02E-01		
	rs6863289	chr5	78,101,389	258	449	215	248	489	195	0.98	7.80E-01	1.08E-01		
	rs2132537	chr5	78,103,740	352	435	135	360	460	112	0.937	3.50E-01	4.56E-01		
	rs17218031	chr5	78,105,411	92	366	463	68	423	441	0.994	9.60E-01	1.77E-02		
	rs7732938	chr5	78,108,516	51	350	520	42	347	543	1.08	3.30E-01	4.81E-01		
	rs16875887	chr5	78,108,781	815	104	3	826	102	4	0.989	9.90E-01	4.36E-03		
	rs7704939	chr5	78,109,954	322	463	137	344	437	151	0.987	8.70E-01	6.05E-02		
	rs754566	chr5	78,110,674	262	465	195	268	464	199	0.997	9.90E-01	4.36E-03		
	rs754567	chr5	78,110,719	388	432	102	385	433	114	1.04	5.60E-01	2.52E-01		
	rs2173012	chr5	78,111,916	7	98	817	2	111	819	0.983	9.60E-01	1.77E-02		
	rs6453415	chr5	78,115,386	21	223	674	22	211	698	1.06	5.50E-01	2.60E-01		
	rs16875913	chr5	78,119,807	748	163	11	767	151	14	0.953	7.00E-01	1.55E-01		
	rs3846677	chr5	78,125,189	616	264	42	630	273	29	0.928	4.00E-01	3.98E-01		
	rs16875919	chr5	78,130,139	15	215	692	24	192	716	1.04	7.50E-01	1.25E-01		
	rs7727110	chr5	78,131,146	69	373	480	59	385	488	1.04	6.50E-01	1.87E-01		
	rs11960862	chr5	78,132,976	567	320	35	574	305	53	1.05	5.30E-01	2.76E-01		
	rs17818103	chr5	78,133,045	124	414	384	127	412	393	1.01	9.40E-01	2.69E-02		
	rs16875940	chr5	78,135,472	803	116	3	793	134	5	1.18	2.10E-01	6.78E-01		
	rs16875951	chr5	78,136,947	14	195	713	18	179	735	1.06	6.30E-01	2.01E-01		
	rs10514108	chr5	78,141,103	5	157	758	9	177	746	0.854	1.70E-01	7.70E-01		
	rs12054837	chr5	78,142,127	796	120	5	792	136	4	1.1	4.80E-01	3.19E-01		
	rs4704524	chr5	78,144,211	770	147	5	754	169	9	1.2	1.20E-01	9.21E-01		
	rs6453416	chr5	78,145,474	732	178	11	722	197	13	1.12	3.10E-01	5.09E-01		
	rs13152961	chr5	78,145,873	38	238	643	38	244	648	0.992	9.60E-01	1.77E-02		
	rs6453417	chr5	78,146,288	262	403	257	283	431	218	0.879	5.40E-02	1.27E+00		
	rs16875961	chr5	78,151,267	688	224	10	712	206	14	0.941	5.70E-01	2.44E-01		
	rs3857404	chr5	78,152,550	124	440	357	115	425	391	1.1	1.80E-01	7.45E-01		
	rs13172853	chr5	78,155,021	5	108	809	3	113	816	1	9.60E-01	1.77E-02		
	rs4704525	chr5	78,159,240	504	361	57	493	386	53	1.03	6.90E-01	1.61E-01		
	rs12655291	chr5	78,159,785	651	245	26	646	261	25	1.04	6.70E-01	1.74E-01		
	rs10058261	chr5	78,162,131	4	90	828	3	95	834	0.98	9.50E-01	2.23E-02		
	rs10454897	chr5	78,167,092	167	462	292	172	453	307	1.02	8.00E-01	9.69E-02		
	rs25413	chr5	78,170,957	203	442	270	238	427	255	0.896	1.00E-01	1.00E+00		
	rs13356637	chr5	78,185,903	592	295	32	599	290	40	1.02	8.00E-01	9.69E-02		
	rs3098681	chr5	78,187,948	112	436	361	134	414	367	0.959	5.70E-01	2.44E-01		

	rs13184327	chr5	78,195,391	458	392	69	452	403	75	1.04	5.70E-01	2.44E-01		
	rs4515284	chr5	78,205,757	29	242	646	19	252	657	1.06	5.70E-01	2.44E-01		
	rs4704531	chr5	78,215,360	32	262	624	23	274	633	1.04	6.90E-01	1.61E-01		
	rs6893857	chr5	78,220,159	595	288	39	598	303	31	0.983	8.70E-01	6.05E-02		
	rs11741352	chr5	78,222,314	167	462	293	175	457	299	0.993	9.40E-01	2.69E-02		
	rs163299	chr5	78,224,171	32	294	584	41	296	577	0.939	4.70E-01	3.28E-01		
	rs163215	chr5	78,226,124	785	132	5	789	138	5	1.03	8.30E-01	8.09E-02		
	rs337875	chr5	78,230,753	376	434	112	403	413	116	0.954	5.10E-01	2.92E-01		
	rs234688	chr5	78,235,210	371	430	116	397	412	117	0.948	4.60E-01	3.37E-01		
	rs337853	chr5	78,238,582	335	417	170	312	461	159	1.03	6.70E-01	1.74E-01		
	rs10052939	chr5	78,239,821	404	398	118	390	447	94	0.984	8.50E-01	7.06E-02		
	rs337849	chr5	78,242,725	76	388	458	70	402	460	1.01	9.20E-01	3.62E-02		
	rs12655738	chr5	78,243,298	111	388	423	82	436	413	1.04	6.00E-01	2.22E-01		
	rs11738153	chr5	78,249,607	424	387	110	408	439	85	0.987	8.80E-01	5.55E-02		
	rs338460	chr5	78,252,634	197	470	255	202	485	245	0.967	6.30E-01	2.01E-01		
	rs10053925	chr5	78,255,785	642	261	19	665	244	23	0.952	6.20E-01	2.08E-01		
	rs337895	chr5	78,257,702	587	305	29	595	301	34	1.01	9.50E-01	2.23E-02		
	rs337887	chr5	78,264,792	250	480	192	252	493	186	0.984	8.30E-01	8.09E-02		
	rs4421085	chr5	78,268,209	30	304	588	34	300	598	1	9.70E-01	1.32E-02		
	rs337844	chr5	78,269,310	30	292	589	34	296	594	0.976	8.00E-01	9.69E-02		
	rs447998	chr5	78,285,889	61	354	507	56	358	518	1.03	7.00E-01	1.55E-01		
	rs234910	chr5	78,293,007	285	456	181	281	473	178	1	9.70E-01	1.32E-02		
	rs7725928	chr5	78,295,401	610	269	32	603	290	29	1.04	6.70E-01	1.74E-01		
	rs337847	chr5	78,295,644	746	164	6	775	150	5	0.886	3.20E-01	4.95E-01		
	rs921945	chr5	78,297,713	613	276	33	605	295	32	1.05	6.10E-01	2.15E-01		
	rs3733895	chr5	78,300,797	391	395	136	369	456	107	0.99	9.10E-01	4.10E-02		
	rs13178105	chr5	78,303,884	35	247	640	23	271	638	1.01	9.20E-01	3.62E-02		
	rs6453423	chr5	78,304,645	70	350	502	61	370	501	1.01	9.30E-01	3.15E-02		
	rs7730428	chr5	78,305,346	70	350	502	61	370	501	1.01	9.30E-01	3.15E-02		
	rs6888024	chr5	78,307,038	588	293	40	608	291	33	0.933	4.20E-01	3.77E-01		
	rs2052550	chr5	78,308,698	51	359	510	58	344	530	1.02	8.20E-01	8.62E-02		
	rs672413	chr5	78,313,985	99	389	434	102	407	423	0.958	5.60E-01	2.52E-01		
	rs163129	chr5	78,315,576	16	200	706	17	187	728	1.07	5.30E-01	2.76E-01		
	rs163125	chr5	78,318,426	627	268	27	614	281	37	1.11	2.30E-01	6.38E-01		
	rs163132	chr5	78,321,677	533	338	50	524	342	65	1.09	2.80E-01	5.53E-01		
	rs336526	chr5	78,323,335	67	360	495	81	367	484	0.923	3.00E-01	5.23E-01		
	rs9293761	chr5	78,325,971	179	441	302	171	451	310	1.03	6.50E-01	1.87E-01		
	rs28326	chr5	78,331,610	604	278	40	612	279	41	0.997	1.00E+00	0.00E+00		
	rs7710824	chr5	78,333,027	48	338	536	60	363	509	0.88	1.00E-01	1.00E+00		
	rs3797535	chr5	78,336,153	3	129	790	5	143	784	0.883	3.40E-01	4.69E-01		
	rs250513	chr5	78,337,952	56	341	523	62	324	544	1.03	7.30E-01	1.37E-01		
	rs479405	chr5	78,338,615	290	466	166	298	448	186	1.03	6.80E-01	1.67E-01		
	rs10514151	chr5	78,339,243	3	123	796	7	116	808	1	9.60E-01	1.77E-02		
	rs570207	chr5	78,343,065	77	408	437	93	380	459	1.01	9.70E-01	1.32E-02		
	rs2445887	chr5	78,345,800	172	453	267	204	425	263	0.922	2.40E-01	6.20E-01		
	rs2034900	chr5	78,351,720	67	355	500	87	368	477	0.88	8.90E-02	1.05E+00		
	rs10514153	chr5	78,352,186	15	191	716	13	219	700	0.9	3.10E-01	5.09E-01		
	rs463614	chr5	78,355,378	263	459	200	290	444	198	0.941	3.70E-01	4.32E-01		
	rs2431332	chr5	78,356,865	507	355	60	537	321	74	0.969	7.00E-01	1.55E-01		
	rs10514154	chr5	78,359,190	642	249	31	625	271	36	1.11	2.40E-01	6.20E-01		
	rs933684	chr5	78,359,831	499	357	66	477	368	87	1.14	8.90E-02	1.05E+00		
	rs1805074	chr5	78,360,108	489	349	66	471	364	87	1.14	9.00E-02	1.05E+00		
	rs402701	chr5	78,366,278	286	459	177	279	447	206	1.08	2.40E-01	6.20E-01		
	rs248383	chr5	78,375,511	268	449	203	238	462	232	1.14	5.50E-02	1.26E+00		
	rs532964	chr5	78,376,042	203	451	268	232	462	238	0.88	5.50E-02	1.26E+00		
	rs6873326	chr5	78,387,268	752	161	9	743	175	14	1.14	2.60E-01	5.85E-01		
	rs7705204	chr5	78,389,703	0	35	887	0	27	905	1.32	3.50E-01	4.56E-01		
	rs1984685	chr5	78,392,192	886	36	0	905	27	0	0.738	2.90E-01	5.38E-01		
	rs642431	chr5	78,400,443	561	304	57	573	301	58	0.983	8.60E-01	6.55E-02		
	rs670220	chr5	78,402,082	57	303	562	57	301	574	1.02	8.30E-01	8.09E-02		
	rs626105	chr5	78,405,657	58	303	561	57	301	574	1.03	7.70E-01	1.14E-01		
	rs682985	chr5	78,409,187	349	428	145	326	452	154	1.08	2.70E-01	5.69E-01		
	rs625879	chr5	78,417,445	141	430	351	153	450	327	0.919	2.20E-01	6.58E-01		
	rs575425	chr5	78,425,668	115	414	392	127	437	364	0.906	1.60E-01	7.96E-01		
	rs586199	chr5	78,433,736	260	450	211	223	468	241	1.16	3.00E-02	1.52E+00		
	rs631305	chr5	78,439,134	38	250	634	40	252	640	0.991	9.50E-01	2.23E-02		
	rs492842	chr5	78,445,743	359	427	133	338	450	140	1.07	3.30E-01	4.81E-01		
	rs6894156	chr5	78,445,901	9	155	758	14	172	746	0.861	1.90E-01	7.21E-01		
	rs6875201	chr5	78,446,320	750	162	9	740	177	14	1.14	2.40E-01	6.20E-01		
	rs3797546	chr5	78,449,260	850	69	0	846	81	0	1.17	3.90E-01	4.09E-01		

	rs567754	chr5	78,452,172	91	430	401	94	414	424	1.04	5.90E-01	2.29E-01		
	rs3733890	chr5	78,457,715	96	357	469	80	402	448	0.979	8.00E-01	9.69E-02		
	rs558133	chr5	78,460,944	422	400	100	422	405	105	1.02	7.90E-01	1.02E-01		
	rs13158309	chr5	78,499,151	346	446	130	357	449	126	0.972	6.90E-01	1.61E-01		
	rs2121107	chr5	78,503,760	98	438	386	95	430	407	1.05	4.90E-01	3.10E-01		
	rs4704546	chr5	78,556,487	99	440	383	101	429	402	1.03	6.60E-01	1.80E-01		
	rs13354432	chr5	78,556,800	826	92	4	841	89	2	0.916	6.00E-01	2.22E-01		
	rs10474578	chr5	78,578,046	339	454	129	357	444	131	0.969	6.70E-01	1.74E-01		
	rs2591392	chr5	78,582,049	119	447	356	116	447	369	1.03	6.70E-01	1.74E-01		
	rs2607142	chr5	78,593,704	338	455	129	357	444	130	0.964	6.10E-01	2.15E-01		
	rs13178271	chr5	78,629,653	0	25	896	0	23	909	1.1	8.50E-01	7.06E-02		
	rs10514159	chr5	78,631,800	138	451	333	127	457	348	1.06	4.40E-01	3.57E-01		
	rs6864184	chr5	78,645,111	162	446	314	142	474	316	1.05	5.20E-01	2.84E-01		
	rs1062326	chr5	78,653,290	120	449	353	117	447	368	1.04	6.20E-01	2.08E-01		
	rs9293769	chr5	78,665,102	342	423	156	341	452	139	0.97	6.70E-01	1.74E-01		
	rs9885487	chr5	78,675,132	69	311	542	52	334	546	1.05	5.70E-01	2.44E-01		
	rs9293775	chr5	78,677,009	68	311	543	53	331	548	1.04	6.00E-01	2.22E-01		
	rs7719464	chr5	78,677,083	734	174	14	735	181	16	1.05	6.90E-01	1.61E-01		
	rs7732902	chr5	78,697,610	896	26	0	910	22	0	0.835	6.40E-01	1.94E-01		
	rs6879014	chr5	78,698,914	108	391	407	101	381	426	1.07	3.90E-01	4.09E-01		
	rs11743792	chr5	78,702,269	56	271	594	40	290	599	1.06	5.30E-01	2.76E-01		
	rs3822568	chr5	78,706,558	388	408	126	397	413	122	0.976	7.50E-01	1.25E-01		
	rs12187625	chr5	78,708,992	198	422	302	190	428	314	1.04	5.60E-01	2.52E-01		
	rs6892318	chr5	78,726,789	406	403	113	437	394	101	0.908	1.80E-01	7.45E-01		
	rs6868393	chr5	78,727,453	112	404	406	101	394	437	1.1	1.90E-01	7.21E-01		
	rs9293779	chr5	78,730,167	409	400	113	437	395	100	0.912	2.00E-01	6.99E-01		
	rs6883030	chr5	78,733,431	814	103	5	830	100	2	0.905	5.20E-01	2.84E-01		
	rs4455546	chr5	78,746,362	9	165	748	8	171	753	0.988	9.60E-01	1.77E-02		
	rs9293780	chr5	78,749,621	546	309	67	575	310	47	0.875	9.60E-02	1.02E+00		
	rs4571454	chr5	78,752,383	113	400	409	98	396	438	1.1	1.70E-01	7.70E-01		
	rs7719054	chr5	78,764,679	626	263	33	633	264	35	1.01	9.90E-01	4.36E-03		
	rs6453450	chr5	78,774,266	425	381	116	446	392	94	0.907	1.80E-01	7.45E-01		
	rs10039490	chr5	78,776,686	70	314	538	48	313	570	1.16	6.10E-02	1.21E+00		
	rs12514775	chr5	78,780,151	22	257	638	33	230	661	1.03	7.80E-01	1.08E-01		
	rs6859667	chr5	78,780,798	804	67	1	811	81	2	1.21	2.80E-01	5.53E-01		
	rs12522716	chr5	78,797,532	739	174	9	739	177	16	1.09	4.60E-01	3.37E-01		
	rs4323213	chr5	78,799,101	283	440	199	311	441	180	0.905	1.40E-01	8.54E-01		
	rs7710089	chr5	78,801,161	483	346	93	502	364	66	0.894	1.40E-01	8.54E-01		
	rs5026822	chr5	78,817,691	908	14	0	913	19	0	1.35	5.00E-01	3.01E-01		
	rs10942889	chr5	78,820,037	307	445	170	320	471	141	0.914	1.90E-01	7.21E-01		
	rs10077726	chr5	78,822,114	687	207	19	705	202	15	0.929	4.80E-01	3.19E-01		
	rs12515981	chr5	78,832,093	651	245	26	645	261	26	1.05	6.00E-01	2.22E-01		
	rs11948804	chr5	78,839,534	7	146	769	6	148	778	1.01	9.70E-01	1.32E-02		
	rs6884494	chr5	78,853,379	8	143	771	7	152	773	0.965	8.10E-01	9.15E-02		
	rs12520582	chr5	78,859,690	814	102	6	820	110	2	0.989	9.90E-01	4.36E-03		
	rs11746854	chr5	78,864,910	599	286	37	583	309	40	1.09	3.30E-01	4.81E-01		
	rs7713917	chr5	78,865,005	393	406	123	357	451	124	1.1	1.90E-01	7.21E-01		
	rs7704425	chr5	78,867,260	31	230	661	24	260	648	0.95	6.00E-01	2.22E-01		
	rs6899277	chr5	78,881,387	373	415	134	347	451	134	1.07	3.50E-01	4.56E-01		
	rs17237492	chr5	78,885,453	577	298	47	575	310	47	1.02	7.90E-01	1.02E-01		
	rs11954003	chr5	78,885,918	729	170	19	763	156	11	0.828	9.00E-02	1.05E+00		
	rs6894248	chr5	78,889,677	106	414	393	111	401	408	1.02	8.10E-01	9.15E-02		
	rs17237849	chr5	78,894,451	5	135	782	2	141	789	1.01	9.70E-01	1.32E-02		
	rs17834549	chr5	78,894,534	781	135	5	789	141	2	0.987	9.60E-01	1.77E-02		
	rs16876942	chr5	78,896,548	799	119	4	796	134	2	1.08	5.80E-01	2.37E-01		
	rs7704901	chr5	78,912,091	410	384	98	432	380	89	0.931	3.40E-01	4.69E-01		
	rs11741539	chr5	78,914,135	385	419	118	404	413	115	0.956	5.40E-01	2.68E-01		
	rs3829808	chr5	78,945,080	747	158	17	785	139	8	0.78	3.30E-02	1.48E+00		
	rs6453465	chr5	78,946,949	16	135	770	7	113	811	1.36	1.40E-02	1.85E+00		
	rs3846680	chr5	78,948,599	9	138	775	9	164	759	0.854	1.90E-01	7.21E-01		
	rs3828602	chr5	78,951,748	774	139	9	759	164	9	1.16	2.10E-01	6.78E-01		
	rs1948764	chr5	78,975,097	17	158	746	8	138	785	1.29	2.80E-02	1.55E+00		
	rs16876991	chr5	78,976,539	11	122	789	3	110	819	1.28	6.80E-02	1.17E+00		
	rs7712162	chr5	78,980,927	41	263	618	34	253	645	1.11	2.60E-01	5.85E-01		
	rs7732613	chr5	78,981,591	746	159	17	785	139	8	0.776	2.90E-02	1.54E+00		
	rs6894524	chr5	78,998,969	611	271	38	628	272	32	0.946	5.40E-01	2.68E-01		
	rs6877794	chr5	78,999,755	75	351	495	67	368	496	1.01	9.00E-01	4.58E-02		
	rs2052476	chr5	79,005,425	9	139	774	9	163	760	0.865	2.30E-01	6.38E-01		
	rs3087813	chr5	79,017,306	41	264	610	35	247	632	1.11	2.40E-01	6.20E-01		
	rs4704576	chr5	79,025,242	241	438	226	236	445	227	1.01	8.70E-01	6.05E-02		

	rs7714250	chr5	79,029,240	28	300	594	35	281	616	1.03	7.40E-01	1.31E-01		
	rs9784710	chr5	79,030,118	23	236	663	20	233	679	1.05	6.10E-01	2.15E-01		
	rs6453473	chr5	79,030,235	227	450	245	227	466	239	0.987	8.70E-01	6.05E-02		
	rs11948957	chr5	79,031,166	800	121	1	804	126	2	1.05	7.60E-01	1.19E-01		
	rs4441859	chr5	79,032,000	435	387	100	435	402	95	0.997	9.90E-01	4.36E-03		
	rs12651804	chr5	79,039,116	51	306	565	37	329	566	1.03	7.40E-01	1.31E-01		
	rs1501913	chr5	79,044,743	15	196	711	17	212	703	0.919	4.20E-01	3.77E-01		
	rs13170837	chr5	79,048,094	386	417	117	385	420	123	1.02	7.80E-01	1.08E-01		
	rs2162805	chr5	79,048,242	4	109	809	1	101	830	1.16	3.20E-01	4.95E-01		
	rs13171893	chr5	79,048,957	450	394	78	446	405	81	1.03	7.20E-01	1.43E-01		
	rs12518982	chr5	79,053,133	585	301	34	568	319	42	1.1	2.40E-01	6.20E-01		
	rs1366271	chr5	79,061,390	3	129	790	4	130	798	0.988	9.70E-01	1.32E-02		
	rs4704585	chr5	79,064,228	118	424	380	129	427	376	0.959	5.70E-01	2.44E-01		
	rs13158477	chr5	79,064,482	648	249	25	665	245	22	0.948	5.80E-01	2.37E-01		
	rs1428224	chr5	79,065,139	790	129	3	797	130	5	1.03	8.70E-01	6.05E-02		
	rs16877150	chr5	79,066,094	814	102	3	813	116	3	1.12	4.40E-01	3.57E-01		
	rs2278239	chr5	79,069,062	790	129	3	797	130	5	1.03	8.70E-01	6.05E-02		
	rs7734306	chr5	79,073,240	116	416	388	127	423	382	0.953	5.10E-01	2.92E-01		
	rs12514461	chr5	79,076,813	746	163	13	731	190	11	1.12	2.90E-01	5.38E-01		
	rs2115127	chr5	79,077,255	3	93	826	3	104	825	0.905	5.30E-01	2.76E-01		
	rs7709188	chr5	79,080,857	41	313	567	41	318	571	0.996	1.00E+00	0.00E+00		
	rs1428226	chr5	79,082,171	726	184	11	713	207	10	1.1	3.60E-01	4.44E-01		
	rs1366272	chr5	79,084,318	10	141	771	12	166	754	0.843	1.40E-01	8.54E-01		
	rs6453481	chr5	79,085,027	29	251	642	27	280	625	0.922	3.70E-01	4.32E-01		
	rs259124	chr5	79,089,290	74	374	441	86	401	403	0.875	7.30E-02	1.14E+00		
	rs259126	chr5	79,093,233	25	270	627	31	291	610	0.899	2.30E-01	6.38E-01		
	rs259128	chr5	79,094,573	12	194	716	12	217	703	0.903	3.30E-01	4.81E-01		
	rs259129	chr5	79,095,173	622	272	28	606	294	32	1.1	2.80E-01	5.53E-01		
	rs6453482	chr5	79,102,789	725	182	15	706	211	15	1.14	2.00E-01	6.99E-01		
	rs109894	chr5	79,103,845	93	393	435	105	427	400	0.883	8.20E-02	1.09E+00		
	rs259121	chr5	79,109,985	388	424	108	408	425	99	0.941	4.00E-01	3.98E-01		
	rs6453484	chr5	79,117,469	585	301	36	629	271	32	0.864	8.80E-02	1.06E+00		
	rs9283795	chr5	79,118,103	28	273	621	27	253	651	1.1	2.90E-01	5.38E-01		
	rs1981970	chr5	79,119,531	0	5	917	1	3	927	1.01	1.00E+00	0.00E+00		
	rs12657828	chr5	79,121,482	700	210	12	693	221	18	1.1	3.50E-01	4.56E-01		
	rs259095	chr5	79,121,753	3	96	823	2	87	843	1.14	4.10E-01	3.87E-01		
	rs1129770	chr5	79,122,639	28	288	606	24	278	630	1.08	3.80E-01	4.20E-01		
	rs2287672	chr5	79,125,284	75	378	469	75	412	445	0.93	3.30E-01	4.81E-01		
	rs259102	chr5	79,128,126	19	229	651	28	220	662	0.976	8.30E-01	8.09E-02		
	rs10491460	chr5	79,129,910	12	127	782	1	125	806	1.22	1.20E-01	9.21E-01		
	rs10043986	chr5	79,131,173	9	208	705	9	224	699	0.936	5.40E-01	2.68E-01		
	rs880179	chr5	79,131,809	3	137	782	5	141	786	0.954	7.40E-01	1.31E-01		
	rs259065	chr5	79,136,473	472	379	71	494	377	61	0.928	3.30E-01	4.81E-01		
	rs410079	chr5	79,140,777	687	213	22	715	203	14	0.874	1.80E-01	7.45E-01		
	rs17405909	chr5	79,141,263	19	214	689	15	228	689	0.985	9.10E-01	4.10E-02		
	rs11957025	chr5	79,142,558	510	338	74	523	349	59	0.935	4.00E-01	3.98E-01		
	rs13185886	chr5	79,144,232	500	354	66	507	355	69	1	9.90E-01	4.36E-03		
	rs259069	chr5	79,146,816	32	272	618	23	254	655	1.16	9.40E-02	1.03E+00		
	rs9293789	chr5	79,151,418	240	470	212	257	449	226	0.994	9.60E-01	1.77E-02		
	rs12514281	chr5	79,155,672	13	177	732	7	171	754	1.12	3.10E-01	5.09E-01		
	rs1864035	chr5	79,156,238	456	374	92	433	390	109	1.12	1.30E-01	8.86E-01		
	rs7707140	chr5	79,156,553	1	34	887	0	36	895	1.01	9.40E-01	2.69E-02		
	rs1529457	chr5	79,165,288	108	435	379	120	428	384	0.977	7.60E-01	1.19E-01		
	rs7380910	chr5	79,168,637	300	472	150	288	479	165	1.06	3.60E-01	4.44E-01		
	rs259074	chr5	79,168,663	159	439	324	147	450	335	1.05	5.00E-01	3.01E-01		
	rs1560130	chr5	79,169,526	755	164	2	787	140	4	0.86	2.20E-01	6.58E-01		
	rs2404666	chr5	79,170,973	76	401	444	67	397	468	1.08	3.20E-01	4.95E-01		
	rs265009	chr5	79,173,312	392	421	109	421	421	90	0.897	1.30E-01	8.86E-01		
	rs918416	chr5	79,177,077	46	301	575	48	319	565	0.946	5.10E-01	2.92E-01		
	rs10056632	chr5	79,177,947	407	417	98	405	412	115	1.06	4.60E-01	3.37E-01		
	rs149767	chr5	79,191,335	54	380	460	55	358	484	1.06	4.40E-01	3.57E-01		
	rs265002	chr5	79,194,908	271	463	187	280	479	173	0.953	4.90E-01	3.10E-01		
	rs9293792	chr5	79,195,244	586	282	36	567	300	44	1.11	2.10E-01	6.78E-01		
	rs264999	chr5	79,196,191	491	366	65	545	333	54	0.842	2.60E-02	1.59E+00		
	rs10942906	chr5	79,196,242	577	315	30	591	304	37	0.996	1.00E+00	0.00E+00		
	rs10942907	chr5	79,197,259	577	315	30	591	304	37	0.996	1.00E+00	0.00E+00		
	rs264989	chr5	79,202,743	376	413	106	354	425	120	1.09	2.10E-01	6.78E-01		
	rs264985	chr5	79,206,357	426	410	86	465	385	82	0.905	1.70E-01	7.70E-01		
	rs264984	chr5	79,206,450	433	409	80	471	382	79	0.914	2.20E-01	6.58E-01		
	rs265005	chr5	79,207,892	482	385	55	500	373	59	0.975	7.60E-01	1.19E-01		

	rs10474594	chr5	79,209,277	362	443	117	363	420	149	1.08	2.70E-01	5.69E-01		
	rs10491455	chr5	79,209,893	585	310	27	590	307	35	1.03	7.40E-01	1.31E-01		
	rs171941	chr5	79,215,882	114	443	365	110	437	385	1.05	4.90E-01	3.10E-01		
	rs265008	chr5	79,217,223	212	432	278	206	479	247	0.946	4.20E-01	3.77E-01		
	rs10514162	chr5	79,222,565	51	376	495	62	345	525	1.04	6.20E-01	2.08E-01		
	rs10514161	chr5	79,222,927	635	256	31	620	279	33	1.09	3.40E-01	4.69E-01		
	rs11745574	chr5	79,223,631	635	256	31	620	279	33	1.09	3.40E-01	4.69E-01		
	rs4704601	chr5	79,223,813	256	485	181	265	476	191	1	9.80E-01	8.77E-03		
	rs4704604	chr5	79,227,458	472	372	77	467	387	78	1.03	7.30E-01	1.37E-01		
	rs13170399	chr5	79,242,826	157	473	291	165	484	281	0.959	5.50E-01	2.60E-01		
	rs1565513	chr5	79,252,657	211	449	258	222	447	258	0.976	7.30E-01	1.37E-01		
	rs13171934	chr5	79,254,754	543	340	39	550	324	58	1.05	5.20E-01	2.84E-01		
	rs1490953	chr5	79,256,899	487	378	56	509	373	50	0.938	4.20E-01	3.77E-01		
	rs12521286	chr5	79,258,951	35	312	575	45	300	587	0.988	9.10E-01	4.10E-02		
	rs10755302	chr5	79,261,182	860	58	4	880	52	0	0.773	2.00E-01	6.99E-01		
	rs6865146	chr5	79,264,026	233	468	221	249	453	230	0.985	8.50E-01	7.06E-02		
	rs12658005	chr5	79,264,275	15	196	711	12	196	724	1.04	7.10E-01	1.49E-01		
	rs6453492	chr5	79,264,405	142	420	360	166	400	366	0.955	5.20E-01	2.84E-01		
	rs7707589	chr5	79,268,812	27	249	611	27	250	624	1.02	9.00E-01	4.58E-02		
	rs7712567	chr5	79,273,243	195	486	240	203	456	267	1.04	5.60E-01	2.52E-01		
	rs4535437	chr5	79,278,419	542	325	55	545	332	55	1.01	9.60E-01	1.77E-02		
	rs10805929	chr5	79,280,778	52	334	536	71	303	558	0.993	9.60E-01	1.77E-02		
	rs1032342	chr5	79,287,099	267	477	178	314	426	192	0.933	3.10E-01	5.09E-01		
	rs2404710	chr5	79,292,318	153	456	313	162	454	316	0.983	8.20E-01	8.62E-02		
	rs4576130	chr5	79,295,781	200	450	270	200	452	280	1.02	7.90E-01	1.02E-01		
	rs6453494	chr5	79,295,975	338	458	126	378	398	156	0.983	8.20E-01	8.62E-02		
	rs11749003	chr5	79,297,740	677	226	19	668	242	22	1.08	4.00E-01	3.98E-01		
	rs1017998	chr5	79,298,967	131	465	326	162	399	371	1.03	7.20E-01	1.43E-01		
	rs17196895	chr5	79,317,968	803	114	5	822	105	5	0.912	5.30E-01	2.76E-01		
	rs1438739	chr5	79,324,751	129	468	325	164	397	371	1.02	7.90E-01	1.02E-01		
	rs7707036	chr5	79,328,655	264	461	193	298	426	207	0.96	5.60E-01	2.52E-01		
	rs383122	chr5	79,334,610	17	204	700	19	184	728	1.1	3.80E-01	4.20E-01		
	rs12332358	chr5	79,341,737	580	295	46	548	346	38	1.1	2.50E-01	6.02E-01		
	rs2451933	chr5	79,342,355	31	298	593	35	257	640	1.14	1.30E-01	8.86E-01		
	rs6878264	chr5	79,343,508	17	251	653	24	231	675	1.04	7.20E-01	1.43E-01		
	rs2028269	chr5	79,344,071	140	466	316	152	446	334	1.01	9.20E-01	3.62E-02		
	rs2118731	chr5	79,353,345	208	498	215	237	448	247	1.01	9.50E-01	2.23E-02		
	rs385771	chr5	79,356,433	601	291	30	647	249	36	0.885	1.60E-01	7.96E-01		
	rs16877442	chr5	79,380,374	757	155	9	762	164	6	1.01	1.00E+00	0.00E+00		
	rs404375	chr5	79,384,515	214	506	202	240	459	233	1.01	8.90E-01	5.06E-02		
	rs401302	chr5	79,391,227	34	290	593	37	259	631	1.11	2.40E-01	6.20E-01		
	rs443095	chr5	79,398,306	1	51	870	0	30	902	1.81	1.30E-02	1.89E+00		
	rs4703797	chr5	79,400,765	427	403	92	416	416	100	1.06	4.50E-01	3.47E-01		
	rs1465853	chr5	79,400,841	912	10	0	917	15	0	1.49	4.40E-01	3.57E-01		
	rs256438	chr5	79,402,005	355	456	110	376	444	112	0.963	6.10E-01	2.15E-01		
	rs13167730	chr5	79,406,003	8	177	737	6	160	766	1.15	2.30E-01	6.38E-01		
	rs2288394	chr5	79,409,559	44	368	510	58	332	542	1.04	6.50E-01	1.87E-01		
	rs10514175	chr5	79,411,987	99	410	412	105	419	408	0.967	6.60E-01	1.80E-01		
	rs2247450	chr5	79,417,552	257	501	162	293	452	185	0.974	7.20E-01	1.43E-01		
	rs2118732	chr5	79,419,032	14	225	679	25	210	689	0.976	8.40E-01	7.57E-02		
	rs2438642	chr5	79,424,501	342	454	126	379	416	137	0.947	4.50E-01	3.47E-01		
	rs2438632	chr5	79,427,949	121	464	337	143	444	345	0.964	6.10E-01	2.15E-01		
	rs16877483	chr5	79,428,859	7	188	727	14	178	740	0.99	9.70E-01	1.32E-02		
	rs2438631	chr5	79,428,900	217	451	254	204	467	261	1.04	5.40E-01	2.68E-01		
	rs425491	chr5	79,431,585	454	374	94	460	383	89	0.982	8.30E-01	8.09E-02		
	rs1465852	chr5	79,432,499	372	413	137	369	421	142	1.02	7.50E-01	1.25E-01		
	rs2434292	chr5	79,434,150	256	467	199	257	463	212	1.03	7.00E-01	1.55E-01		
	rs6865426	chr5	79,434,465	18	258	646	14	247	671	1.1	3.40E-01	4.69E-01		
	rs2438625	chr5	79,434,480	91	362	469	82	373	477	1.03	6.70E-01	1.74E-01		
	rs2099651	chr5	79,434,667	192	471	259	207	464	261	0.971	6.80E-01	1.67E-01		
	rs2015459	chr5	79,436,534	70	385	466	67	365	500	1.09	2.50E-01	6.02E-01		
	rs726436	chr5	79,436,687	2	124	796	10	129	793	0.859	2.50E-01	6.02E-01		
	rs6862233	chr5	79,437,046	466	386	70	500	366	66	0.913	2.30E-01	6.38E-01		
	rs421837	chr5	79,437,173	57	332	533	63	346	523	0.941	4.50E-01	3.47E-01		
	rs6863009	chr5	79,437,447	769	143	10	801	120	11	0.85	2.00E-01	6.99E-01		
	rs4704608	chr5	79,437,992	2	121	799	7	129	796	0.875	3.20E-01	4.95E-01		
	rs4704609	chr5	79,438,199	19	248	655	15	239	678	1.09	3.80E-01	4.20E-01		
	rs4704610	chr5	79,447,004	33	294	595	32	288	612	1.04	6.50E-01	1.87E-01		
	rs10474010	chr5	79,447,199	26	287	609	34	283	615	0.971	7.60E-01	1.19E-01		
	rs4703798	chr5	79,448,084	352	433	137	374	439	119	0.917	2.20E-01	6.58E-01		

	rs13328157	chr5	79,453,334	841	79	1	844	82	6	1.15	4.00E-01	3.98E-01		
	rs439274	chr5	79,454,132	27	248	647	26	256	650	0.989	9.40E-01	2.69E-02		
	rs10474608	chr5	79,457,936	632	261	29	648	262	22	0.939	5.00E-01	3.01E-01		
	rs1470025	chr5	79,458,185	426	392	104	437	409	86	0.939	3.90E-01	4.09E-01		
	rs11950944	chr5	79,458,749	367	420	130	338	446	141	1.1	1.70E-01	7.70E-01		
	rs10057542	chr5	79,459,103	11	142	767	7	147	777	1.03	8.20E-01	8.62E-02		
	rs10072008	chr5	79,471,439	12	178	732	11	194	727	0.939	5.80E-01	2.37E-01		
	rs4704618	chr5	79,473,085	304	445	173	296	460	176	1.03	7.10E-01	1.49E-01		
	rs4704619	chr5	79,473,195	104	394	424	90	403	439	1.07	3.90E-01	4.09E-01		
	rs7707754	chr5	79,474,682	46	326	550	45	341	546	0.975	7.70E-01	1.14E-01		
	rs7712447	chr5	79,478,925	27	264	631	24	288	620	0.948	5.60E-01	2.52E-01		
	rs1132801	chr5	79,496,866	658	235	29	654	256	22	1.02	9.00E-01	4.58E-02		
	rs6893475	chr5	79,496,911	539	331	52	537	346	49	1.01	9.00E-01	4.58E-02		
	rs13176625	chr5	79,498,278	530	315	77	525	342	65	0.994	9.70E-01	1.32E-02		
	rs7721370	chr5	79,501,959	55	286	581	45	335	551	0.925	3.40E-01	4.69E-01		
	rs11959429	chr5	79,505,285	612	266	44	594	297	39	1.06	4.90E-01	3.10E-01		
	rs13186313	chr5	79,505,787	530	321	69	506	357	66	1.07	3.60E-01	4.44E-01		
	rs10050980	chr5	79,507,139	106	416	400	92	412	428	1.1	1.90E-01	7.21E-01		
	rs6890275	chr5	79,509,042	33	215	673	24	248	659	0.952	6.20E-01	2.08E-01		
	rs4704624	chr5	79,516,376	182	463	274	163	468	299	1.1	1.70E-01	7.70E-01		
	rs4704627	chr5	79,523,947	97	405	420	104	411	417	0.968	6.60E-01	1.80E-01		
	rs4704629	chr5	79,527,619	392	405	125	375	438	119	1.03	6.60E-01	1.80E-01		
	rs6876176	chr5	79,531,212	73	356	492	73	385	474	0.94	4.20E-01	3.77E-01		
	rs4704633	chr5	79,532,695	13	197	712	12	201	719	1	9.80E-01	8.77E-03		
	rs4704635	chr5	79,533,089	179	452	284	177	472	283	0.998	1.00E+00	0.00E+00		
	rs12519047	chr5	79,533,936	13	198	711	11	203	718	1.01	9.80E-01	8.77E-03		
	rs6868932	chr5	79,535,251	15	172	735	8	174	750	1.08	4.80E-01	3.19E-01		
	rs6880905	chr5	79,537,571	14	154	754	7	161	764	1.06	6.60E-01	1.80E-01		
	rs13188069	chr5	79,540,445	27	205	690	17	223	692	1.02	8.60E-01	6.55E-02		
	rs17259420	chr5	79,541,813	37	247	638	22	265	644	1.06	5.40E-01	2.68E-01		
	rs6872447	chr5	79,542,950	76	347	499	61	364	507	1.05	5.20E-01	2.84E-01		
	rs10068511	chr5	79,545,051	41	234	647	29	269	634	0.972	7.80E-01	1.08E-01		
	rs10036776	chr5	79,545,730	105	371	442	83	426	419	0.988	9.00E-01	4.58E-02		
	rs6863905	chr5	79,547,228	707	195	20	715	201	15	0.97	7.90E-01	1.02E-01		
	rs6895353	chr5	79,550,431	367	407	148	344	458	130	1.02	8.30E-01	8.09E-02		
	rs7711197	chr5	79,552,682	14	165	743	7	170	755	1.07	5.90E-01	2.29E-01		
	rs13355369	chr5	79,554,034	190	414	318	175	458	299	0.988	8.90E-01	5.06E-02		
	rs4704641	chr5	79,557,262	190	414	318	175	459	298	0.986	8.60E-01	6.55E-02		
	rs7726099	chr5	79,557,460	320	411	190	314	445	173	0.979	7.80E-01	1.08E-01		
	rs13355153	chr5	79,558,361	45	232	645	31	275	626	0.959	6.50E-01	1.87E-01		
	rs12522787	chr5	79,562,102	19	216	686	20	216	696	1	1.00E+00	0.00E+00		
	rs16877647	chr5	79,566,062	17	228	676	15	221	696	1.07	5.30E-01	2.76E-01		
	rs17200856	chr5	79,568,000	34	234	654	24	264	644	0.974	8.00E-01	9.69E-02		
	rs12189352	chr5	79,570,857	318	439	165	297	450	184	1.1	1.80E-01	7.45E-01		
	rs2279772	chr5	79,571,190	11	196	715	5	178	749	1.2	1.00E-01	1.00E+00		
	rs1010809	chr5	79,578,880	23	236	661	23	237	672	1.01	9.40E-01	2.69E-02		
	rs881708	chr5	79,585,440	118	411	393	105	425	402	1.05	5.40E-01	2.68E-01		
	rs1126176	chr5	79,587,940	113	431	378	122	450	360	0.933	3.30E-01	4.81E-01		
	rs6872359	chr5	79,600,641	72	364	448	61	349	486	1.13	1.10E-01	9.59E-01		
	rs13190128	chr5	79,600,702	731	176	10	731	189	9	1.05	7.00E-01	1.55E-01		
	rs9293809	chr5	79,600,827	361	424	134	356	427	147	1.05	5.10E-01	2.92E-01		
	rs10053040	chr5	79,603,167	674	227	21	660	255	17	1.07	4.60E-01	3.37E-01		
	rs950788	chr5	79,613,516	340	413	169	330	440	162	1.01	9.00E-01	4.58E-02		
	rs2173676	chr5	79,614,553	522	342	54	490	376	63	1.14	8.80E-02	1.06E+00		
	rs4704655	chr5	79,616,574	703	161	21	720	170	16	0.968	8.00E-01	9.69E-02		
	rs9293811	chr5	79,621,841	735	173	14	723	201	8	1.08	5.10E-01	2.92E-01		
	rs6886552	chr5	79,626,430	19	251	652	20	212	700	1.19	7.00E-02	1.15E+00		
	rs11738136	chr5	79,634,345	100	422	400	86	402	444	1.14	6.00E-02	1.22E+00		
	rs10074004	chr5	79,634,402	465	358	98	425	423	84	1.08	3.00E-01	5.23E-01		
	rs10054927	chr5	79,636,264	28	215	678	23	268	641	0.852	8.30E-02	1.08E+00		
	rs4499817	chr5	79,636,281	115	434	373	128	443	361	0.938	3.60E-01	4.44E-01		
	rs4998063	chr5	79,636,449	183	473	261	165	444	319	1.18	1.50E-02	1.82E+00		
	rs10805934	chr5	79,639,464	212	441	268	183	490	256	1.04	6.10E-01	2.15E-01		
	rs10056190	chr5	79,646,769	284	453	185	272	478	181	1.02	7.90E-01	1.02E-01		
	rs7720696	chr5	79,647,944	421	386	111	391	441	98	1.05	4.90E-01	3.10E-01		
	rs1500138	chr5	79,649,697	113	387	422	98	440	394	0.961	6.00E-01	2.22E-01		
	rs11744364	chr5	79,651,345	51	318	553	37	337	558	1.04	6.20E-01	2.08E-01		
	rs16876315	chr5	79,653,113	670	232	20	665	253	14	1.03	8.20E-01	8.62E-02		
	rs10060273	chr5	79,654,405	20	219	683	16	196	720	1.17	1.10E-01	9.59E-01		
	rs2405378	chr5	79,658,228	40	322	560	40	295	597	1.11	2.20E-01	6.58E-01		

	rs6862946	chr5	79,672,928	663	235	24	659	256	17	1.02	8.90E-01	5.06E-02		
	rs6863361	chr5	79,673,140	516	338	68	510	370	52	0.986	8.80E-01	5.55E-02		
	rs13175654	chr5	79,673,555	73	343	506	53	379	499	1.02	7.70E-01	1.14E-01		
	rs13177823	chr5	79,679,214	73	344	502	52	378	496	1.03	6.90E-01	1.61E-01		
	rs11960365	chr5	79,686,480	27	255	640	22	275	635	0.975	8.10E-01	9.15E-02		
	rs1392392	chr5	79,699,756	186	439	297	175	495	262	0.947	4.30E-01	3.67E-01		
	rs924447	chr5	79,700,929	476	375	71	454	402	76	1.09	2.70E-01	5.69E-01		
	rs10062770	chr5	79,701,391	282	452	188	288	471	173	0.957	5.30E-01	2.76E-01		
	rs7715187	chr5	79,701,787	11	176	734	9	179	744	1.02	9.00E-01	4.58E-02		
	rs12515731	chr5	79,702,245	1	98	823	1	81	850	1.23	2.00E-01	6.99E-01		
	rs9293817	chr5	79,715,230	29	252	641	33	250	649	0.99	9.40E-01	2.69E-02		
	rs4704666	chr5	79,716,474	278	438	205	267	476	189	0.991	9.20E-01	3.62E-02		
	rs6861026	chr5	79,717,483	72	378	472	75	375	482	1.01	9.50E-01	2.23E-02		
	rs259035	chr5	79,731,126	771	143	8	787	139	6	0.934	6.10E-01	2.15E-01		
	rs259047	chr5	79,736,160	820	100	2	847	83	2	0.819	2.10E-01	6.78E-01		
	rs13188088	chr5	79,737,595	637	258	27	634	273	25	1.03	7.70E-01	1.14E-01		
	rs192313	chr5	79,757,476	777	137	8	792	134	6	0.939	6.50E-01	1.87E-01		
	rs2544600	chr5	79,768,835	15	275	632	29	266	636	0.941	5.10E-01	2.92E-01		
	rs259028	chr5	79,770,053	2	100	820	2	82	848	1.24	1.80E-01	7.45E-01		
	rs194539	chr5	79,773,425	516	343	63	523	346	63	0.994	9.70E-01	1.32E-02		
	rs259033	chr5	79,775,441	733	137	5	761	131	6	0.943	6.80E-01	1.67E-01		
	rs249038	chr5	79,781,225	2	100	820	2	81	846	1.25	1.60E-01	7.96E-01		
	rs11958178	chr5	79,784,138	0	1	921	0	0	932	0	5.00E-01	3.01E-01		
	rs249008	chr5	79,841,521	83	405	434	79	386	466	1.09	2.60E-01	5.85E-01		
	rs171598	chr5	79,856,480	44	317	560	41	307	582	1.07	4.50E-01	3.47E-01		
	rs2443549	chr5	79,869,577	790	129	3	797	129	6	1.04	8.30E-01	8.09E-02		
	rs9687094	chr5	79,877,439	590	287	45	562	326	44	1.11	2.00E-01	6.99E-01		
	rs2014233	chr5	79,883,132	270	452	200	254	482	196	1.03	7.00E-01	1.55E-01		
	rs32857	chr5	79,891,128	18	233	671	19	232	681	1.01	9.70E-01	1.32E-02		
	rs249209	chr5	79,902,965	324	445	153	311	460	161	1.05	4.70E-01	3.28E-01		
	rs13182894	chr5	79,906,192	529	331	61	511	370	51	1.04	6.30E-01	2.01E-01		
	rs185105	chr5	79,906,483	266	452	204	253	474	205	1.03	6.50E-01	1.87E-01		
	rs6872791	chr5	79,909,730	829	87	3	838	89	4	1.03	9.00E-01	4.58E-02		
	rs383833	chr5	79,919,304	19	240	663	20	240	672	1	1.00E+00	0.00E+00		
	rs2431222	chr5	79,923,247	743	172	7	756	170	6	0.965	7.80E-01	1.08E-01		
	rs2405867	chr5	79,924,206	26	259	637	30	268	634	0.95	5.90E-01	2.29E-01		
	rs4327585	chr5	79,924,251	825	96	1	821	107	4	1.17	2.90E-01	5.38E-01		
	rs13164793	chr5	79,924,873	14	202	705	16	221	695	0.909	3.50E-01	4.56E-01		
	rs865635	chr5	79,926,421	7	149	766	5	155	772	0.998	9.60E-01	1.77E-02		
	rs6883095	chr5	79,926,803	266	452	204	253	480	199	1.02	8.00E-01	9.69E-02		
	rs1072841	chr5	79,928,959	485	343	94	454	404	74	1.04	6.10E-01	2.15E-01		
	rs4703814	chr5	79,929,471	490	357	75	488	368	76	1.02	8.10E-01	9.15E-02		
	rs836788	chr5	79,947,800	110	434	378	119	407	406	1.04	6.10E-01	2.15E-01		
	rs1232027	chr5	79,950,776	96	412	414	102	410	420	0.992	9.30E-01	3.15E-02		
	rs12517451	chr5	79,955,829	59	357	506	42	343	547	1.17	4.70E-02	1.33E+00		
	rs1643659	chr5	79,970,592	488	370	64	494	356	82	1.05	5.70E-01	2.44E-01		
	rs1677693	chr5	79,972,074	64	370	487	82	356	494	0.958	5.80E-01	2.37E-01		
	rs844370	chr5	79,978,758	488	370	64	494	356	82	1.05	5.70E-01	2.44E-01		
	rs13161245	chr5	79,980,489	488	370	64	494	356	82	1.05	5.70E-01	2.44E-01		
	rs10072026	chr5	79,980,896	749	162	11	730	188	14	1.18	1.30E-01	8.86E-01		
	rs380691	chr5	79,987,790	437	381	103	430	395	106	1.03	6.60E-01	1.80E-01		
	rs6151616	chr5	79,997,226	626	271	25	634	268	30	1.01	9.10E-01	4.10E-02		
